# Supplementary figures and images for: Ultrasound classification of non-mass breast lesions following BI-RADS presents high positive predictive value (part 2 of 2)
Source: PLoS One. 2022 Nov 30;17(11):e0278299. doi: 10.1371/journal.pone.0278299 (PMC9710769; doi:10.1371/journal.pone.0278299)

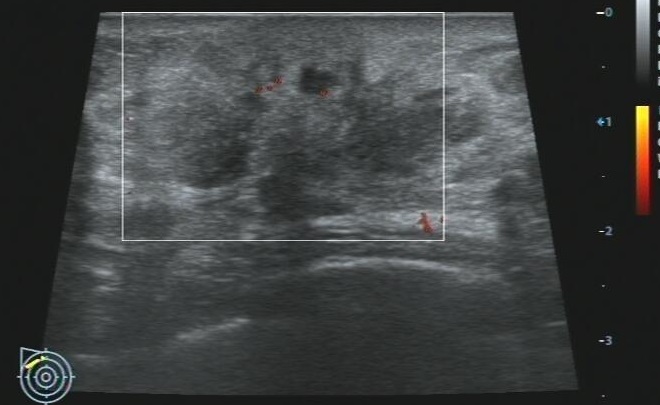

Supplement: S2 Data — Representative sonographic images of breast non-mass lesions. (ZIP) [file pone.0278299.s002.zip › Supplementary data 2/33/a.jpg]

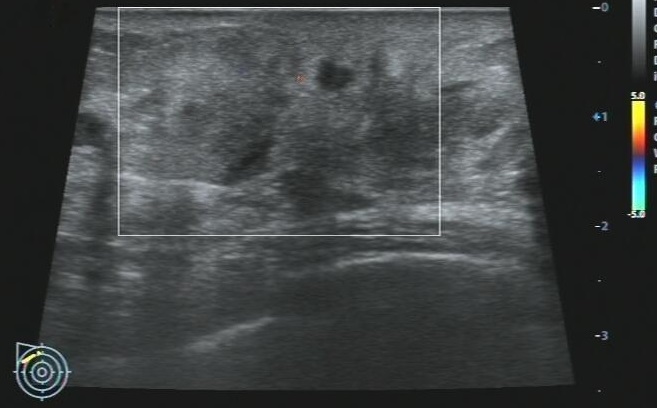

Supplement: S2 Data — Representative sonographic images of breast non-mass lesions. (ZIP) [file pone.0278299.s002.zip › Supplementary data 2/33/b.jpg]

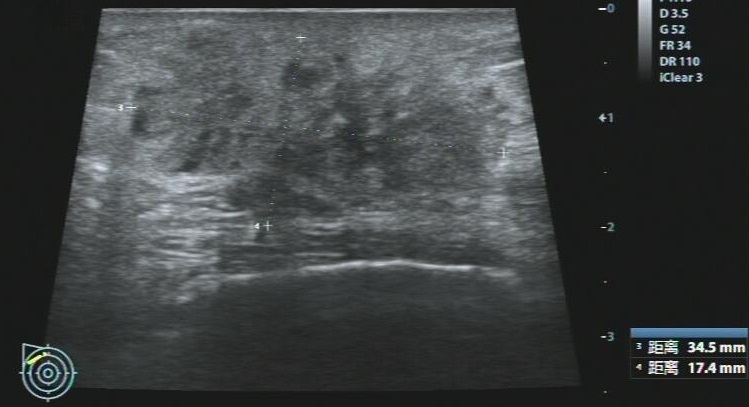

Supplement: S2 Data — Representative sonographic images of breast non-mass lesions. (ZIP) [file pone.0278299.s002.zip › Supplementary data 2/33/c.jpg]

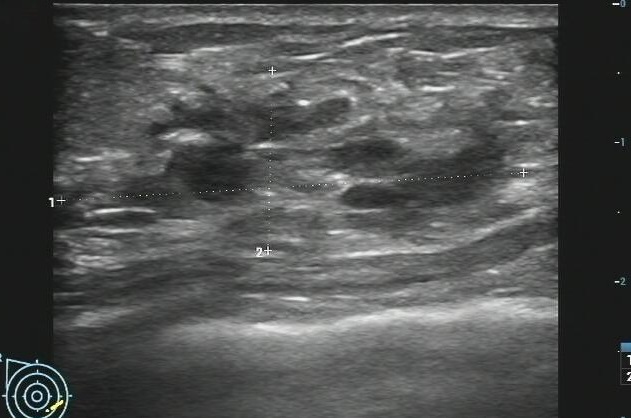

Supplement: S2 Data — Representative sonographic images of breast non-mass lesions. (ZIP) [file pone.0278299.s002.zip › Supplementary data 2/34/a.jpg]

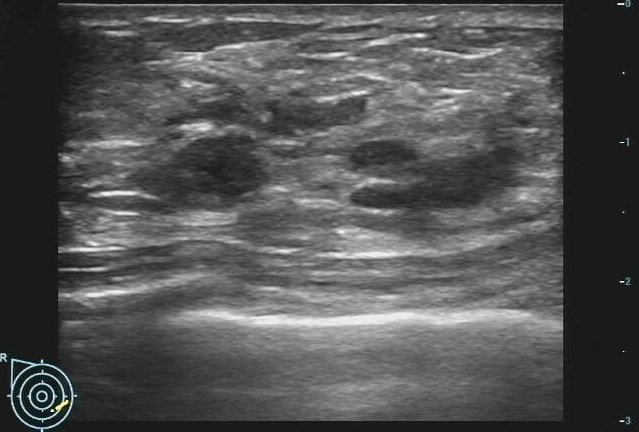

Supplement: S2 Data — Representative sonographic images of breast non-mass lesions. (ZIP) [file pone.0278299.s002.zip › Supplementary data 2/34/b.jpg]

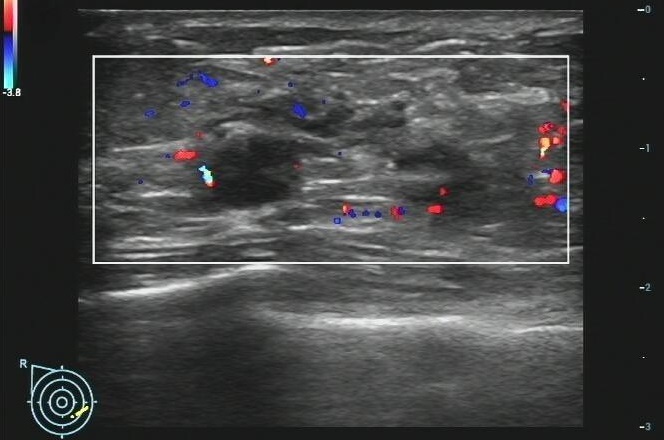

Supplement: S2 Data — Representative sonographic images of breast non-mass lesions. (ZIP) [file pone.0278299.s002.zip › Supplementary data 2/34/c.jpg]

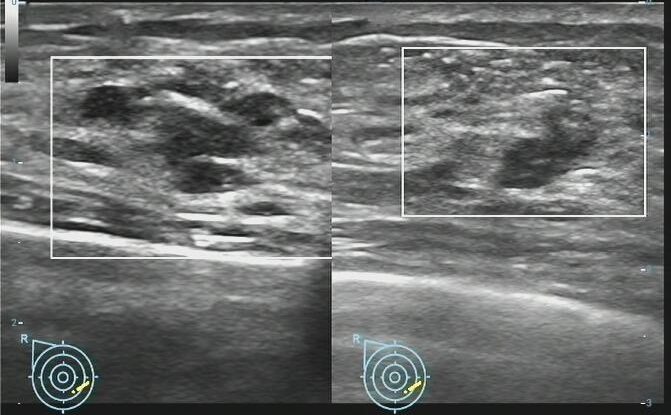

Supplement: S2 Data — Representative sonographic images of breast non-mass lesions. (ZIP) [file pone.0278299.s002.zip › Supplementary data 2/34/d.jpg]

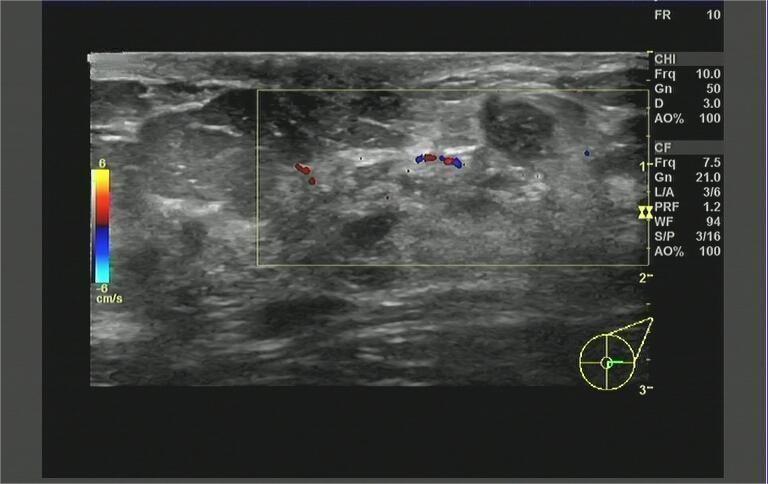

Supplement: S2 Data — Representative sonographic images of breast non-mass lesions. (ZIP) [file pone.0278299.s002.zip › Supplementary data 2/35/a.jpg]

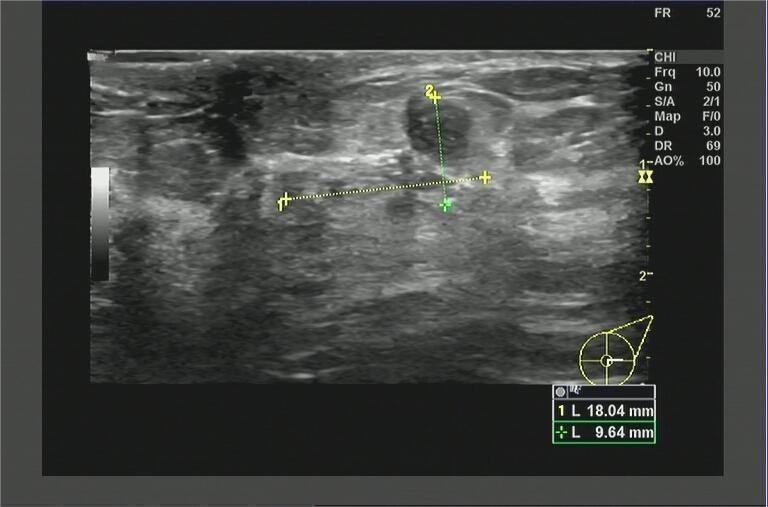

Supplement: S2 Data — Representative sonographic images of breast non-mass lesions. (ZIP) [file pone.0278299.s002.zip › Supplementary data 2/35/b.jpg]

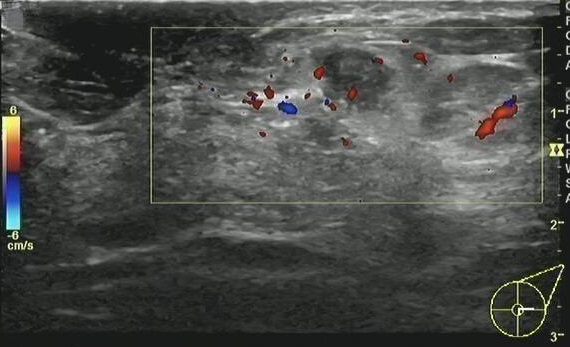

Supplement: S2 Data — Representative sonographic images of breast non-mass lesions. (ZIP) [file pone.0278299.s002.zip › Supplementary data 2/35/c.jpg]

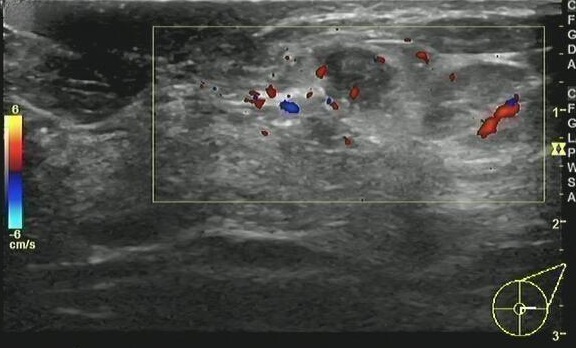

Supplement: S2 Data — Representative sonographic images of breast non-mass lesions. (ZIP) [file pone.0278299.s002.zip › Supplementary data 2/35/d.jpg]

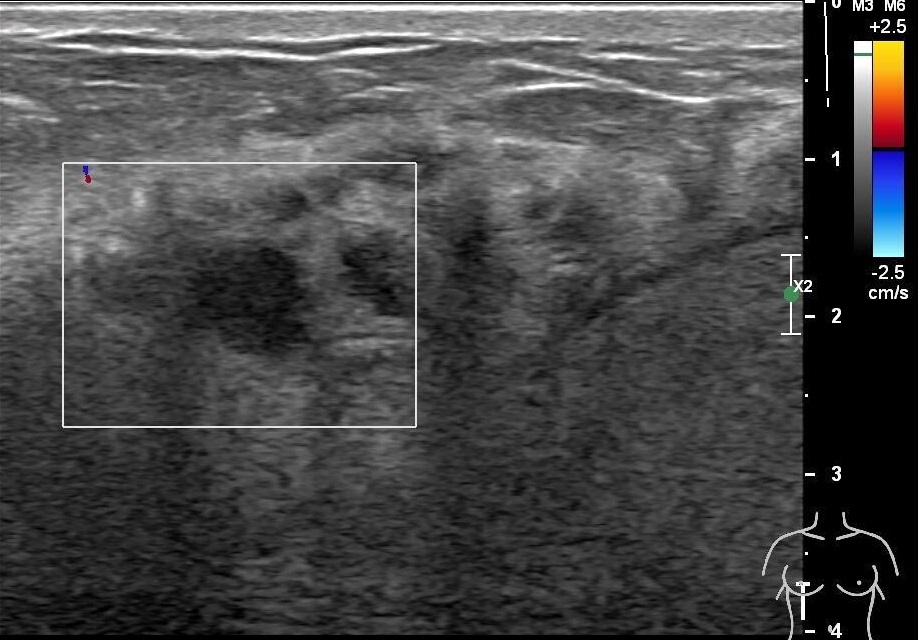

Supplement: S2 Data — Representative sonographic images of breast non-mass lesions. (ZIP) [file pone.0278299.s002.zip › Supplementary data 2/36/a.jpg]

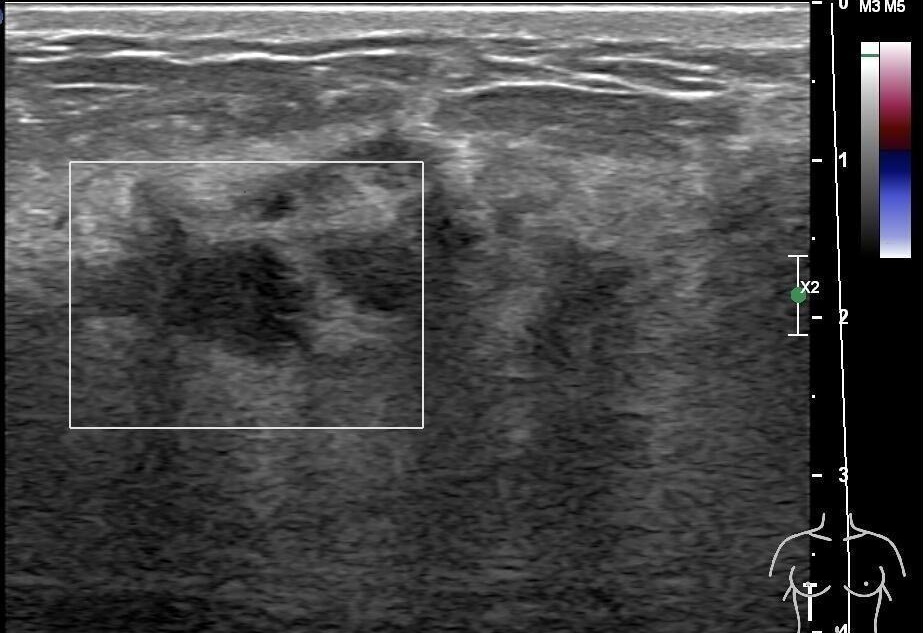

Supplement: S2 Data — Representative sonographic images of breast non-mass lesions. (ZIP) [file pone.0278299.s002.zip › Supplementary data 2/36/b.jpg]

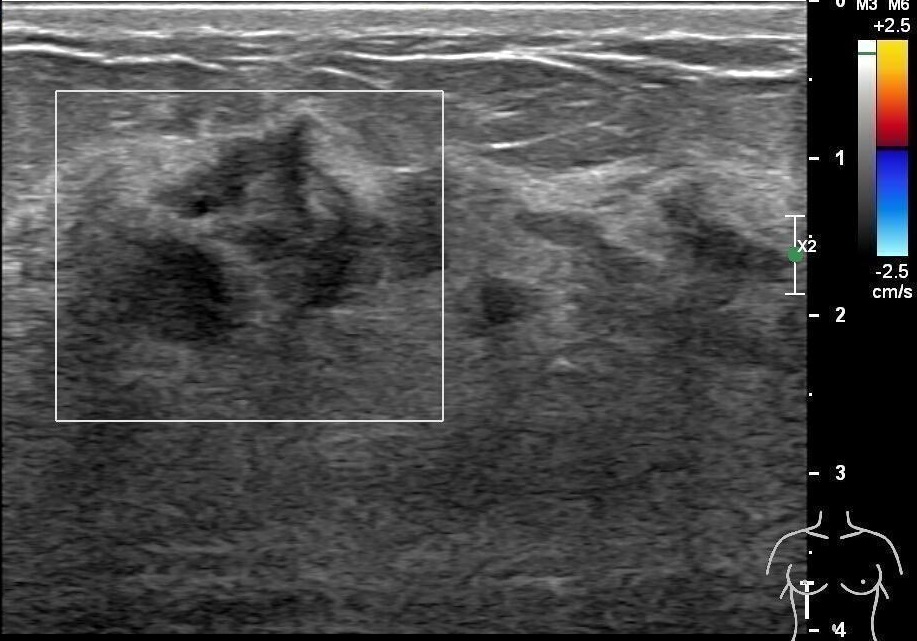

Supplement: S2 Data — Representative sonographic images of breast non-mass lesions. (ZIP) [file pone.0278299.s002.zip › Supplementary data 2/36/c.jpg]

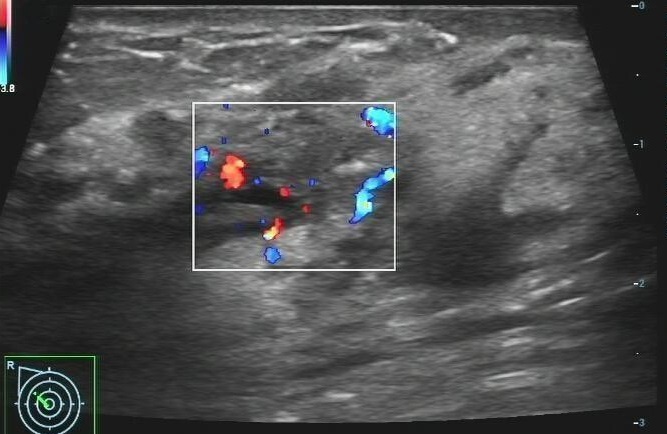

Supplement: S2 Data — Representative sonographic images of breast non-mass lesions. (ZIP) [file pone.0278299.s002.zip › Supplementary data 2/37/a.jpg]

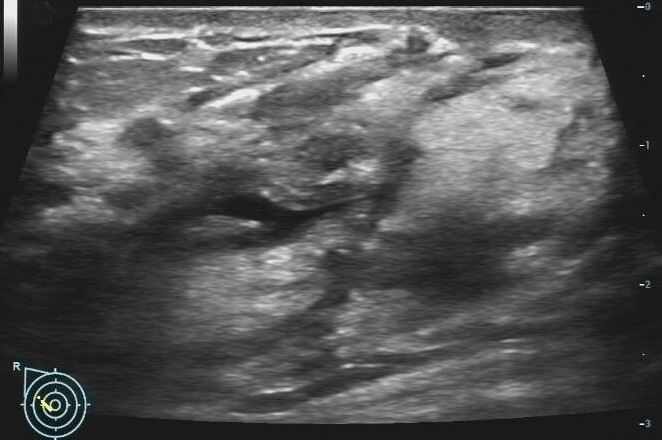

Supplement: S2 Data — Representative sonographic images of breast non-mass lesions. (ZIP) [file pone.0278299.s002.zip › Supplementary data 2/37/b.jpg]

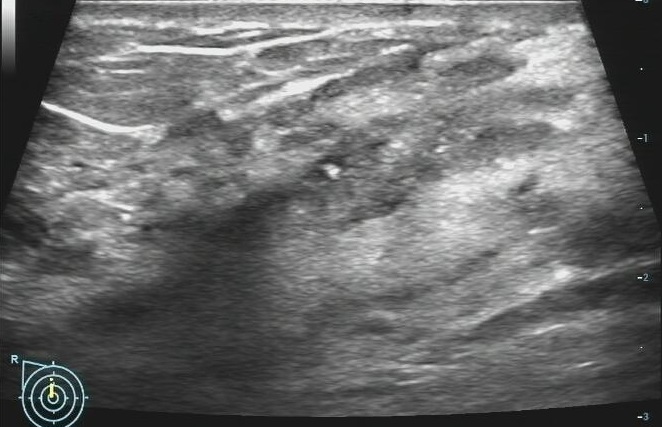

Supplement: S2 Data — Representative sonographic images of breast non-mass lesions. (ZIP) [file pone.0278299.s002.zip › Supplementary data 2/37/c.jpg]

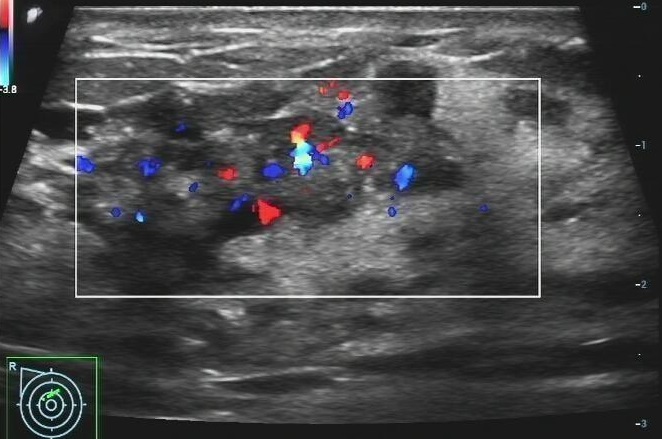

Supplement: S2 Data — Representative sonographic images of breast non-mass lesions. (ZIP) [file pone.0278299.s002.zip › Supplementary data 2/37/d.jpg]

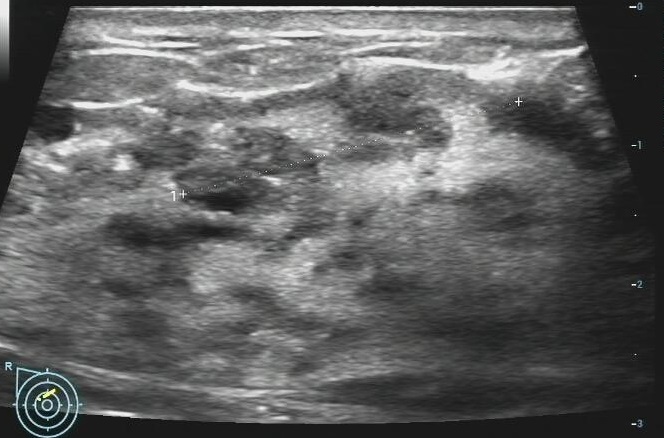

Supplement: S2 Data — Representative sonographic images of breast non-mass lesions. (ZIP) [file pone.0278299.s002.zip › Supplementary data 2/37/e.jpg]

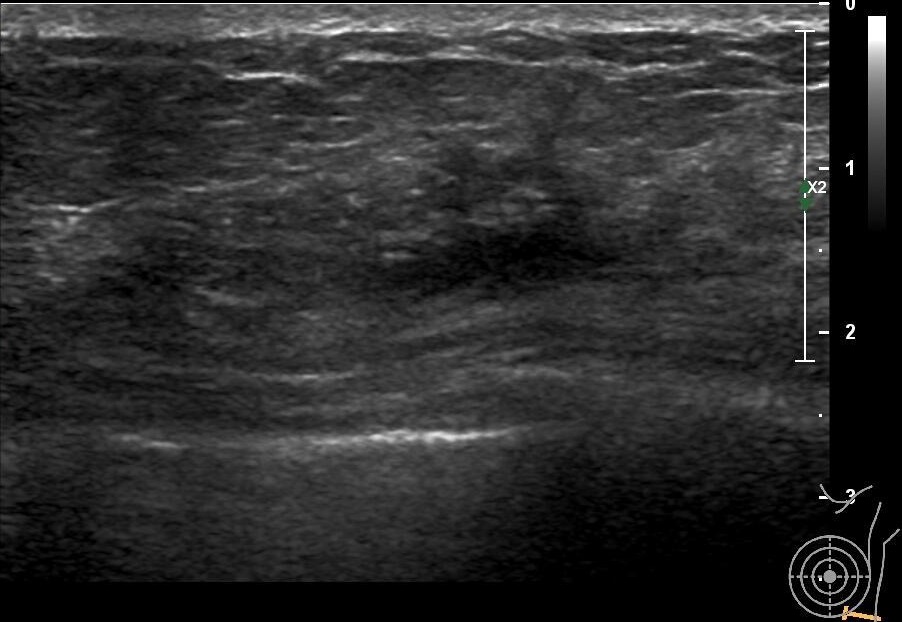

Supplement: S2 Data — Representative sonographic images of breast non-mass lesions. (ZIP) [file pone.0278299.s002.zip › Supplementary data 2/38/a.jpg]

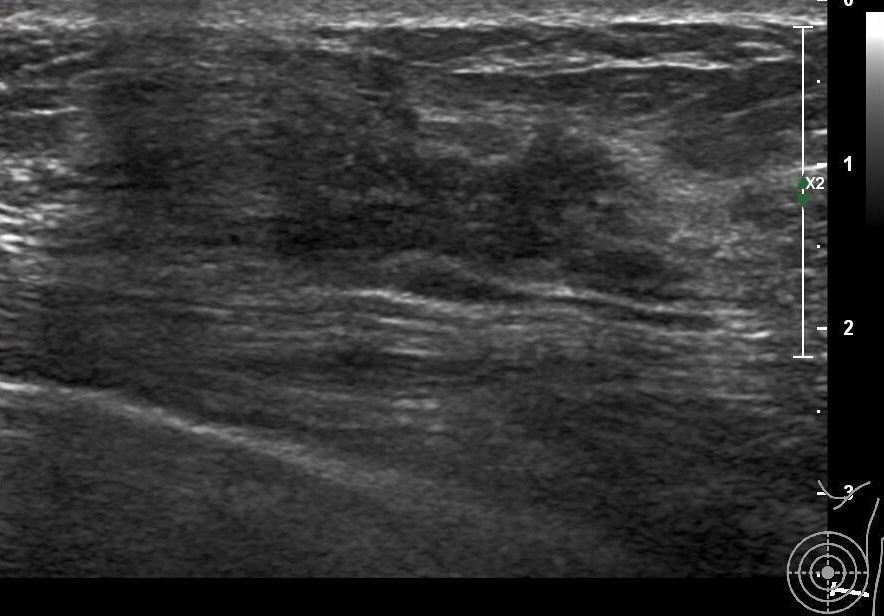

Supplement: S2 Data — Representative sonographic images of breast non-mass lesions. (ZIP) [file pone.0278299.s002.zip › Supplementary data 2/38/b.jpg]

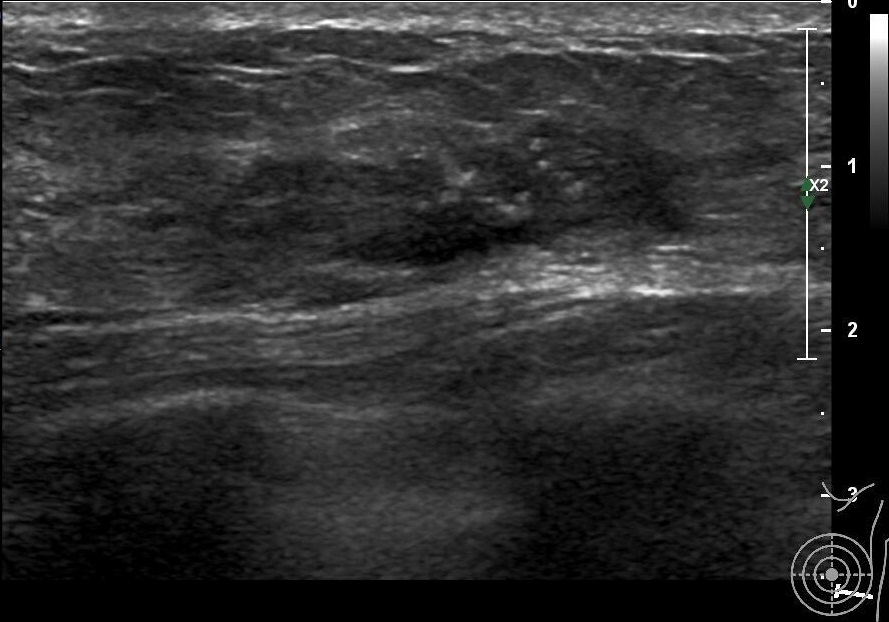

Supplement: S2 Data — Representative sonographic images of breast non-mass lesions. (ZIP) [file pone.0278299.s002.zip › Supplementary data 2/38/c.jpg]

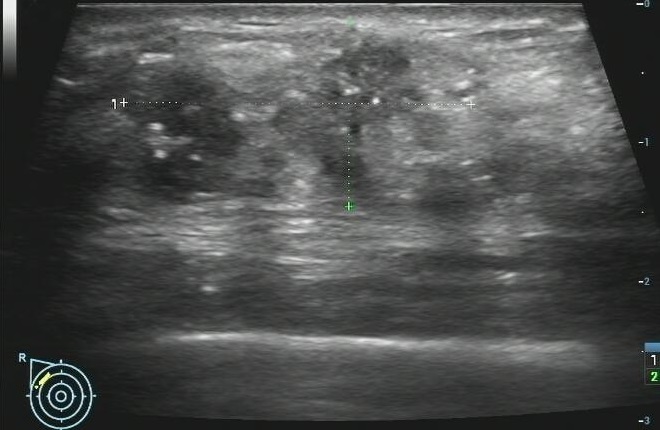

Supplement: S2 Data — Representative sonographic images of breast non-mass lesions. (ZIP) [file pone.0278299.s002.zip › Supplementary data 2/39/a.jpg]

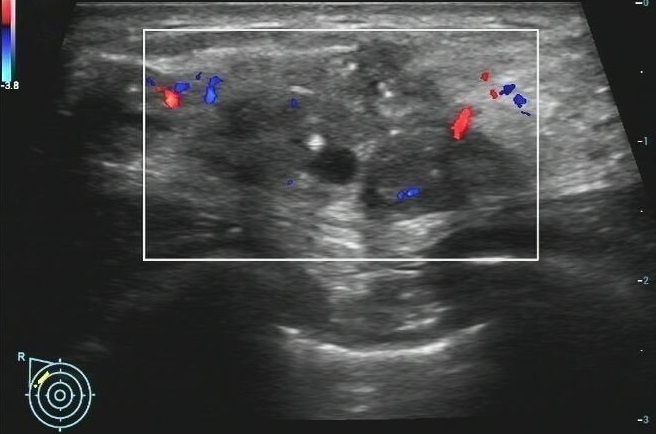

Supplement: S2 Data — Representative sonographic images of breast non-mass lesions. (ZIP) [file pone.0278299.s002.zip › Supplementary data 2/39/b.jpg]

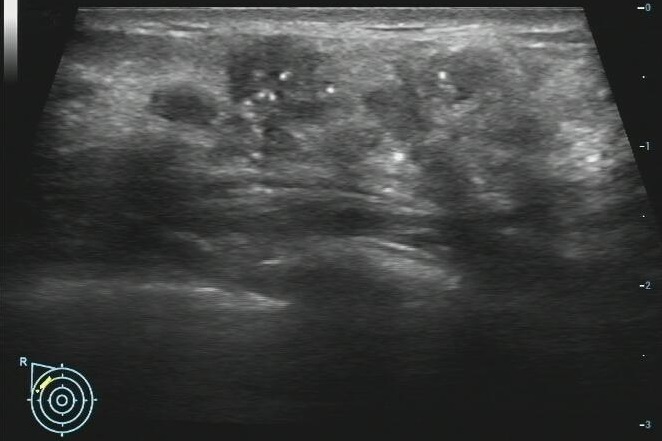

Supplement: S2 Data — Representative sonographic images of breast non-mass lesions. (ZIP) [file pone.0278299.s002.zip › Supplementary data 2/39/c.jpg]

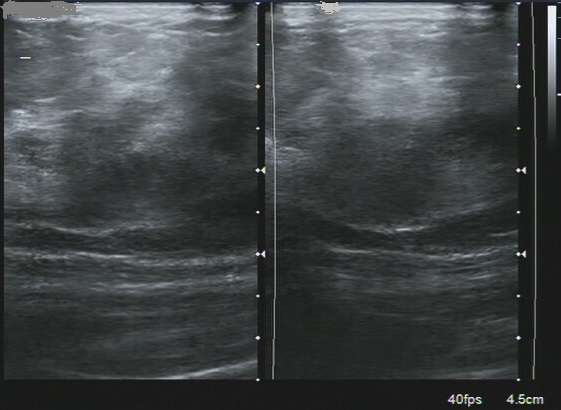

Supplement: S2 Data — Representative sonographic images of breast non-mass lesions. (ZIP) [file pone.0278299.s002.zip › Supplementary data 2/4/a.jpg]

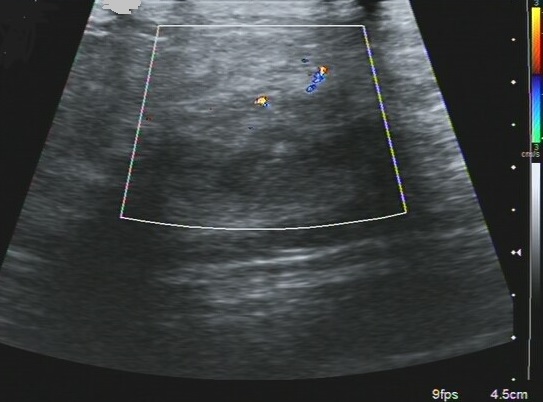

Supplement: S2 Data — Representative sonographic images of breast non-mass lesions. (ZIP) [file pone.0278299.s002.zip › Supplementary data 2/4/b.jpg]

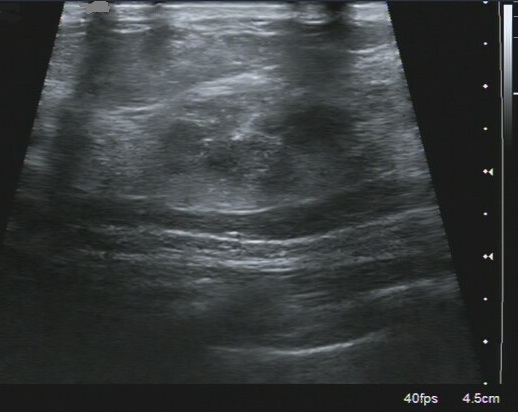

Supplement: S2 Data — Representative sonographic images of breast non-mass lesions. (ZIP) [file pone.0278299.s002.zip › Supplementary data 2/4/c.jpg]

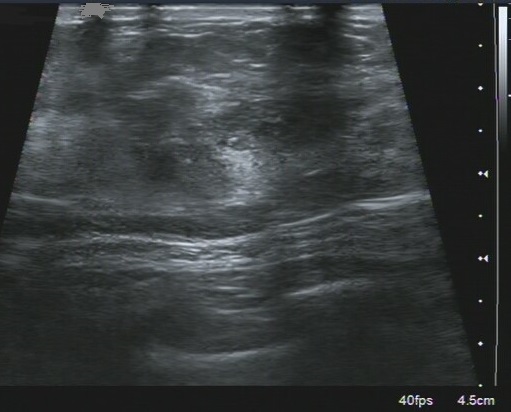

Supplement: S2 Data — Representative sonographic images of breast non-mass lesions. (ZIP) [file pone.0278299.s002.zip › Supplementary data 2/4/d.jpg]

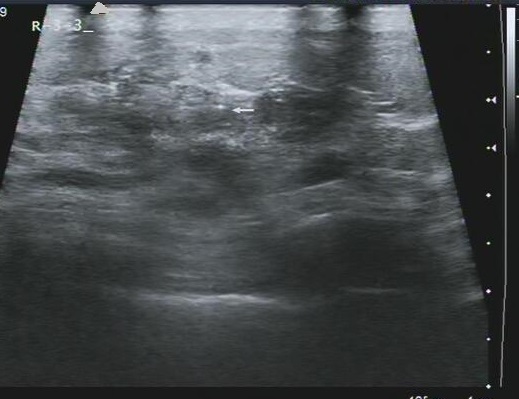

Supplement: S2 Data — Representative sonographic images of breast non-mass lesions. (ZIP) [file pone.0278299.s002.zip › Supplementary data 2/40/a.jpg]

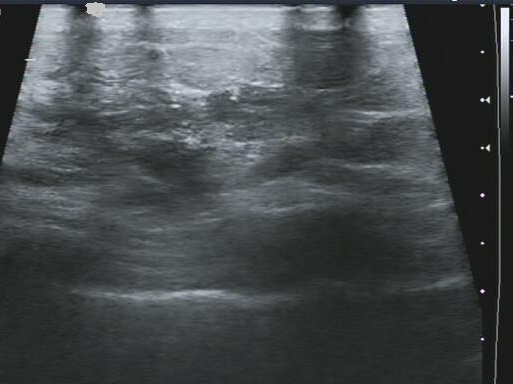

Supplement: S2 Data — Representative sonographic images of breast non-mass lesions. (ZIP) [file pone.0278299.s002.zip › Supplementary data 2/40/b.jpg]

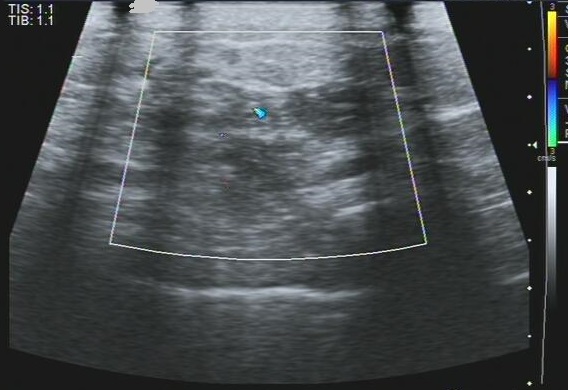

Supplement: S2 Data — Representative sonographic images of breast non-mass lesions. (ZIP) [file pone.0278299.s002.zip › Supplementary data 2/40/c.jpg]

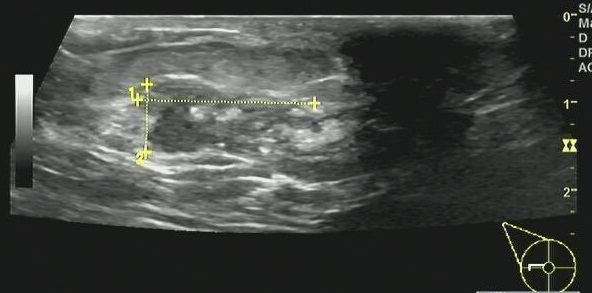

Supplement: S2 Data — Representative sonographic images of breast non-mass lesions. (ZIP) [file pone.0278299.s002.zip › Supplementary data 2/41/a.jpg]

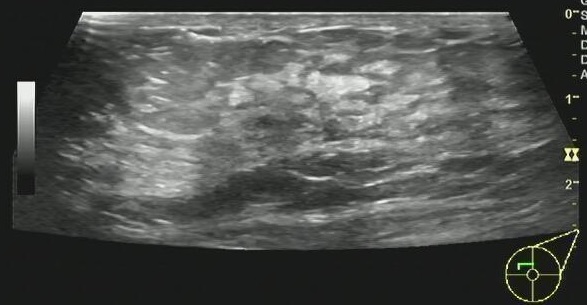

Supplement: S2 Data — Representative sonographic images of breast non-mass lesions. (ZIP) [file pone.0278299.s002.zip › Supplementary data 2/41/b.jpg]

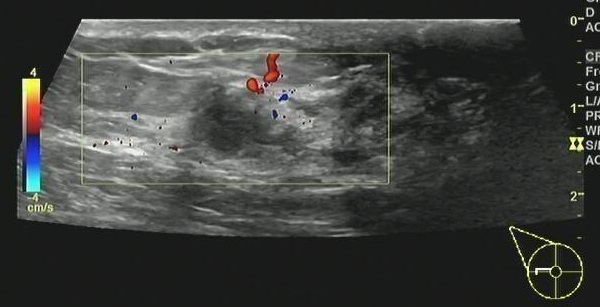

Supplement: S2 Data — Representative sonographic images of breast non-mass lesions. (ZIP) [file pone.0278299.s002.zip › Supplementary data 2/41/c.jpg]

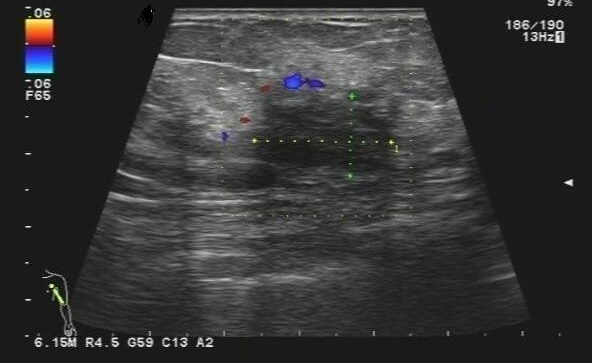

Supplement: S2 Data — Representative sonographic images of breast non-mass lesions. (ZIP) [file pone.0278299.s002.zip › Supplementary data 2/42/a.jpg]

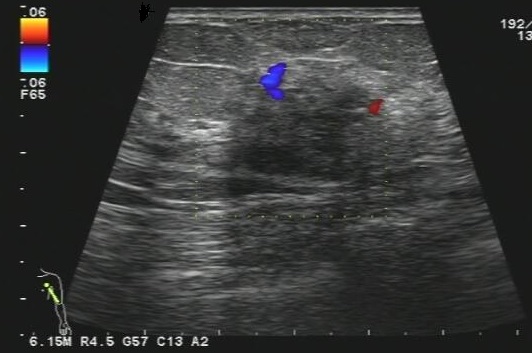

Supplement: S2 Data — Representative sonographic images of breast non-mass lesions. (ZIP) [file pone.0278299.s002.zip › Supplementary data 2/42/b.jpg]

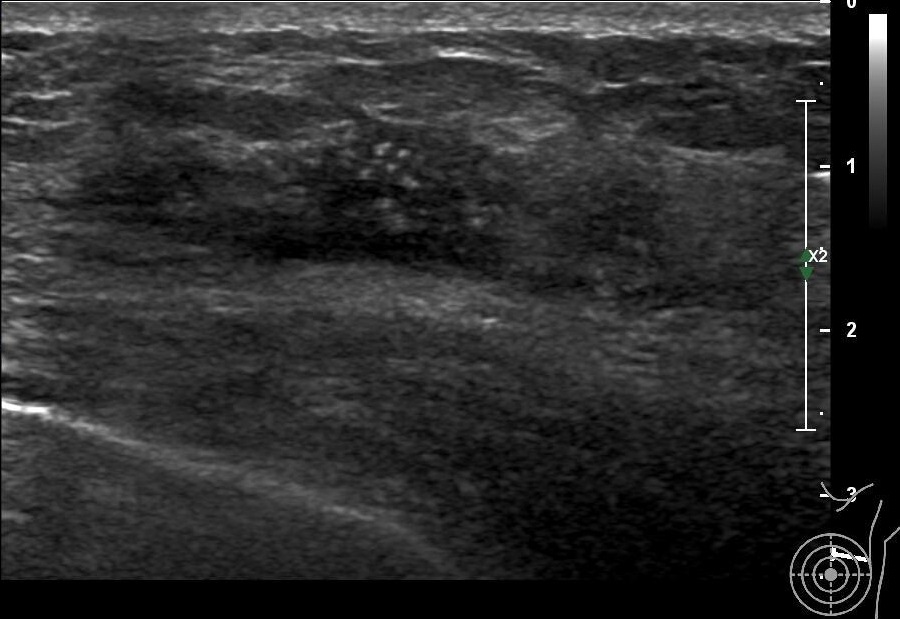

Supplement: S2 Data — Representative sonographic images of breast non-mass lesions. (ZIP) [file pone.0278299.s002.zip › Supplementary data 2/43/a.jpg]

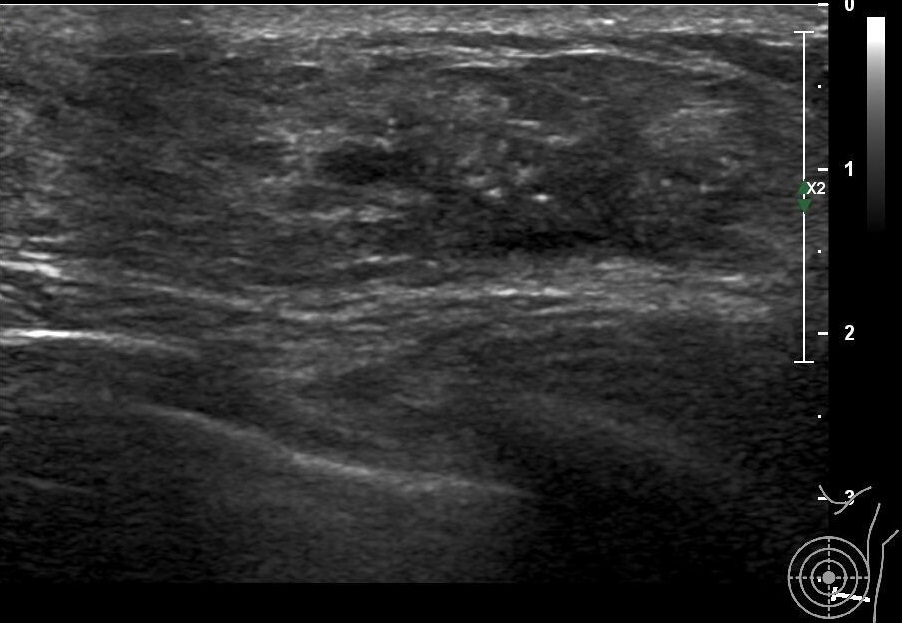

Supplement: S2 Data — Representative sonographic images of breast non-mass lesions. (ZIP) [file pone.0278299.s002.zip › Supplementary data 2/43/b.jpg]

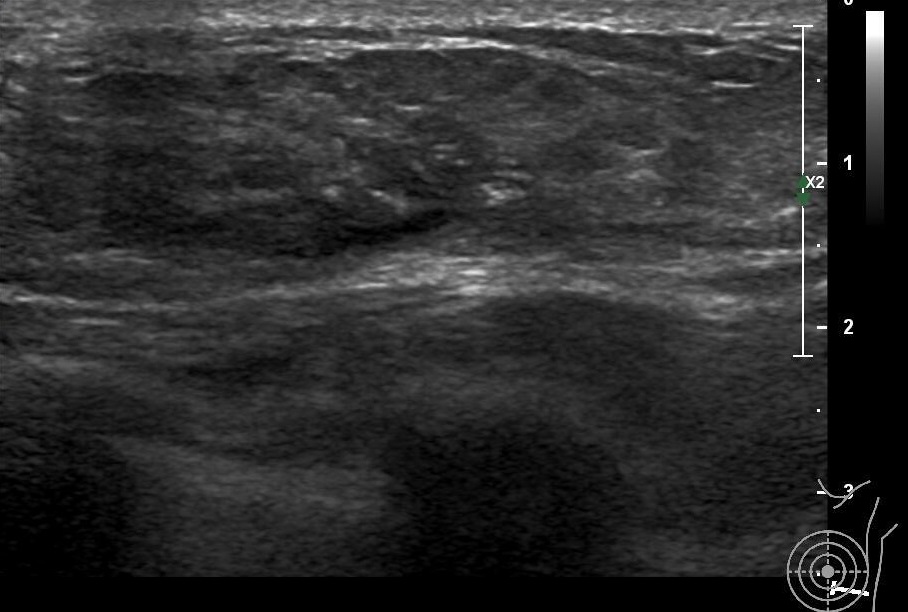

Supplement: S2 Data — Representative sonographic images of breast non-mass lesions. (ZIP) [file pone.0278299.s002.zip › Supplementary data 2/43/c.jpg]

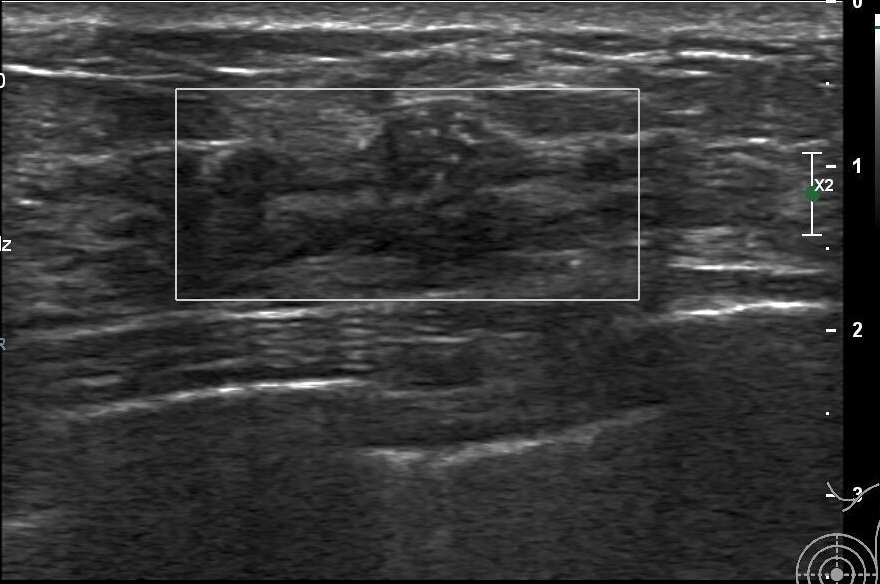

Supplement: S2 Data — Representative sonographic images of breast non-mass lesions. (ZIP) [file pone.0278299.s002.zip › Supplementary data 2/43/d.jpg]

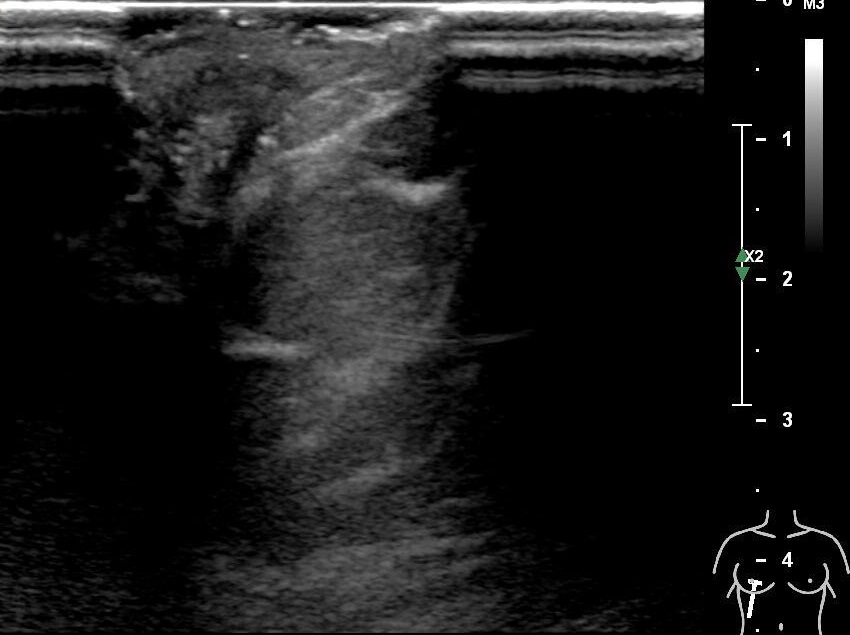

Supplement: S2 Data — Representative sonographic images of breast non-mass lesions. (ZIP) [file pone.0278299.s002.zip › Supplementary data 2/44/a.jpg]

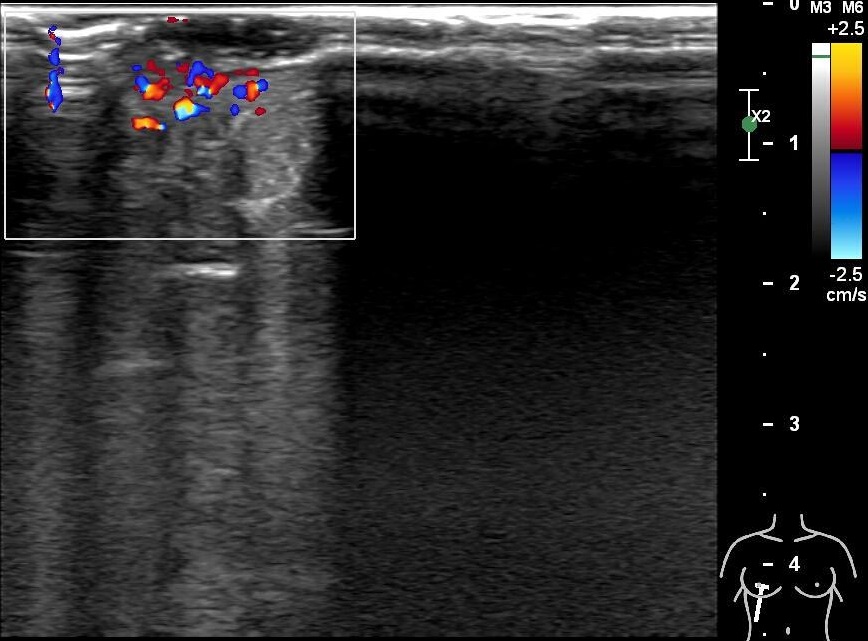

Supplement: S2 Data — Representative sonographic images of breast non-mass lesions. (ZIP) [file pone.0278299.s002.zip › Supplementary data 2/44/b.jpg]

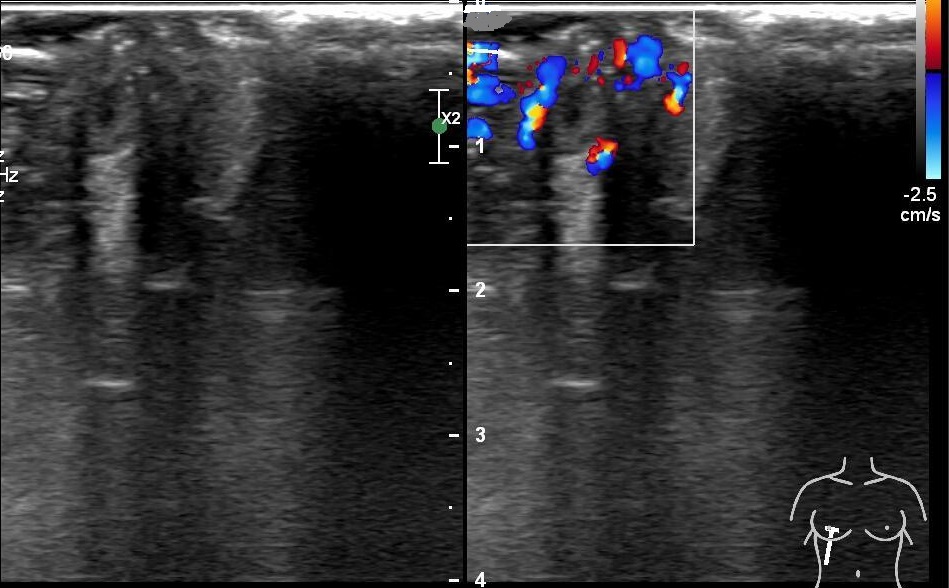

Supplement: S2 Data — Representative sonographic images of breast non-mass lesions. (ZIP) [file pone.0278299.s002.zip › Supplementary data 2/44/c.jpg]

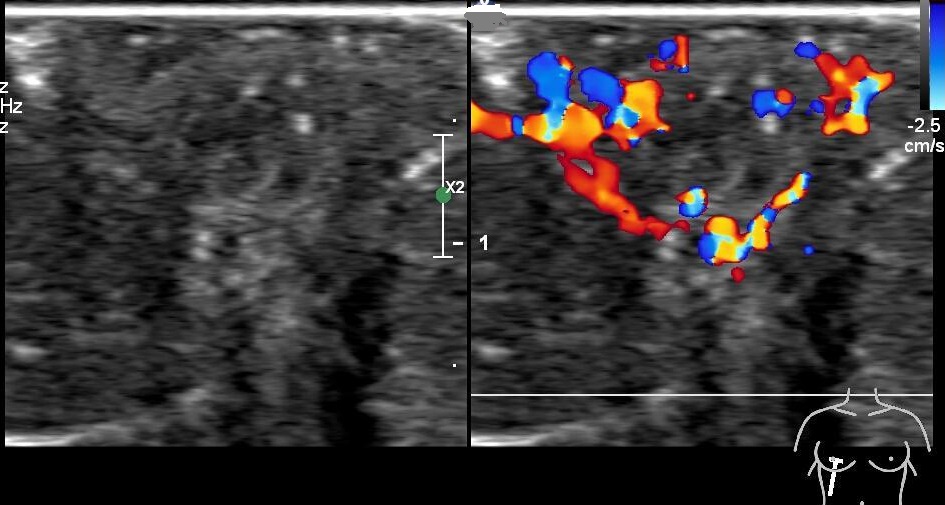

Supplement: S2 Data — Representative sonographic images of breast non-mass lesions. (ZIP) [file pone.0278299.s002.zip › Supplementary data 2/44/d.jpg]

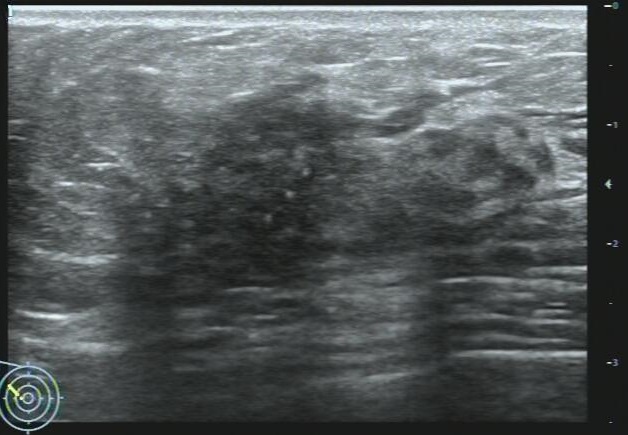

Supplement: S2 Data — Representative sonographic images of breast non-mass lesions. (ZIP) [file pone.0278299.s002.zip › Supplementary data 2/45/a.jpg]

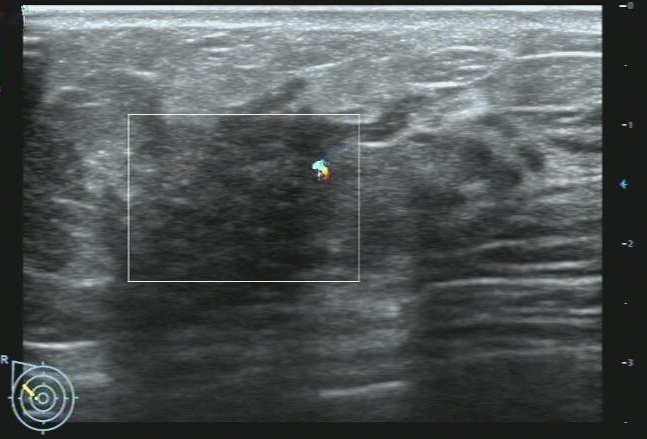

Supplement: S2 Data — Representative sonographic images of breast non-mass lesions. (ZIP) [file pone.0278299.s002.zip › Supplementary data 2/45/b.jpg]

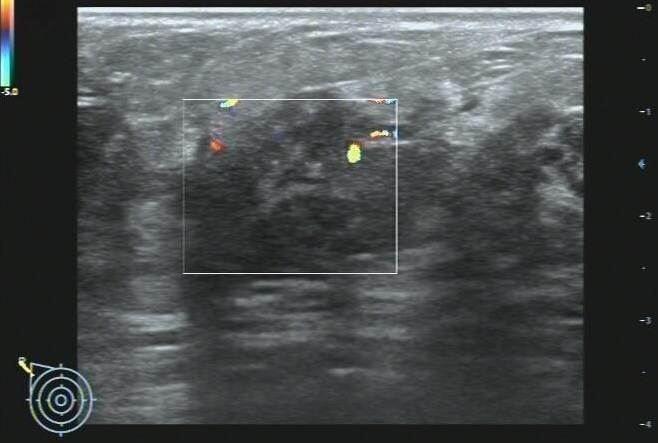

Supplement: S2 Data — Representative sonographic images of breast non-mass lesions. (ZIP) [file pone.0278299.s002.zip › Supplementary data 2/45/c.jpg]

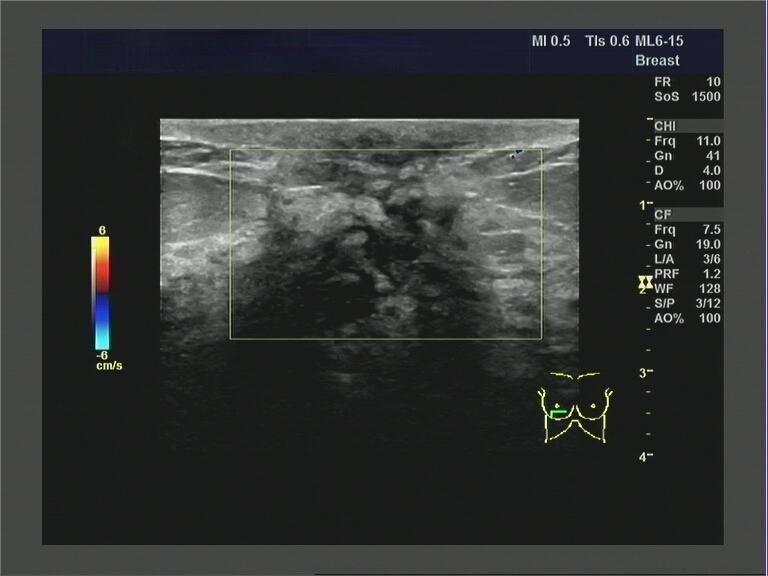

Supplement: S2 Data — Representative sonographic images of breast non-mass lesions. (ZIP) [file pone.0278299.s002.zip › Supplementary data 2/46/a.jpg]

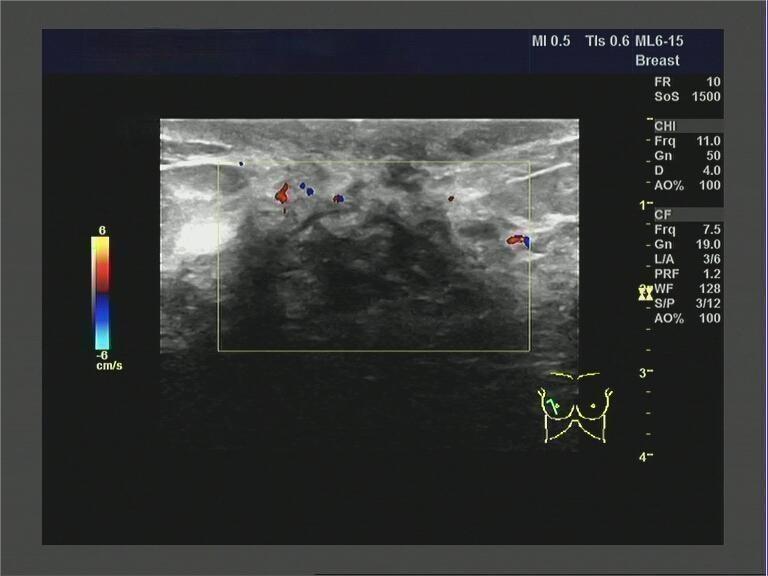

Supplement: S2 Data — Representative sonographic images of breast non-mass lesions. (ZIP) [file pone.0278299.s002.zip › Supplementary data 2/46/b.jpg]

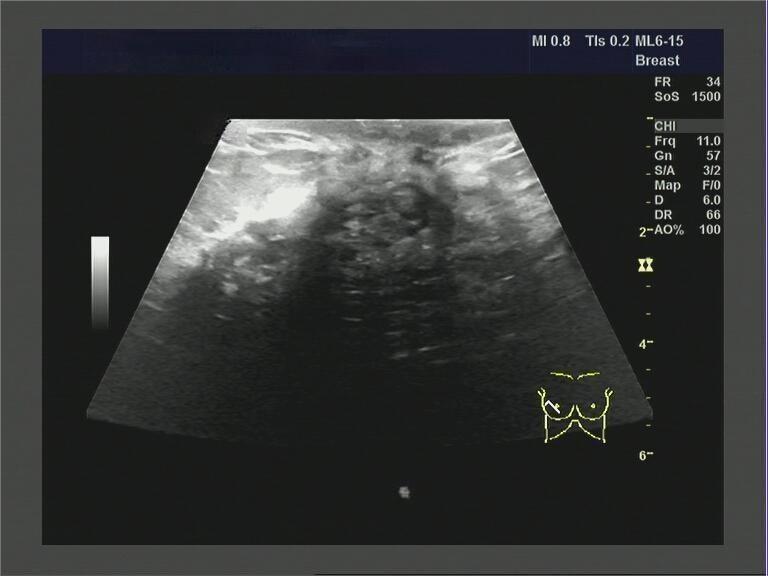

Supplement: S2 Data — Representative sonographic images of breast non-mass lesions. (ZIP) [file pone.0278299.s002.zip › Supplementary data 2/46/c.jpg]

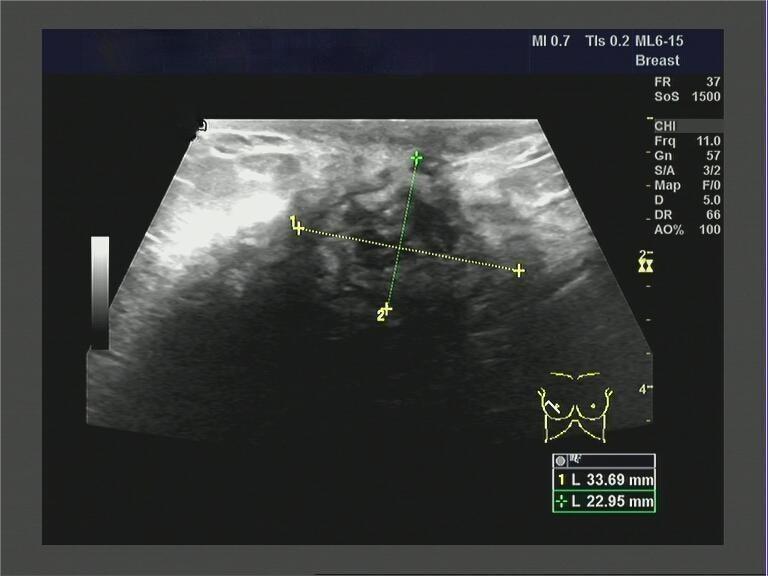

Supplement: S2 Data — Representative sonographic images of breast non-mass lesions. (ZIP) [file pone.0278299.s002.zip › Supplementary data 2/46/d.jpg]

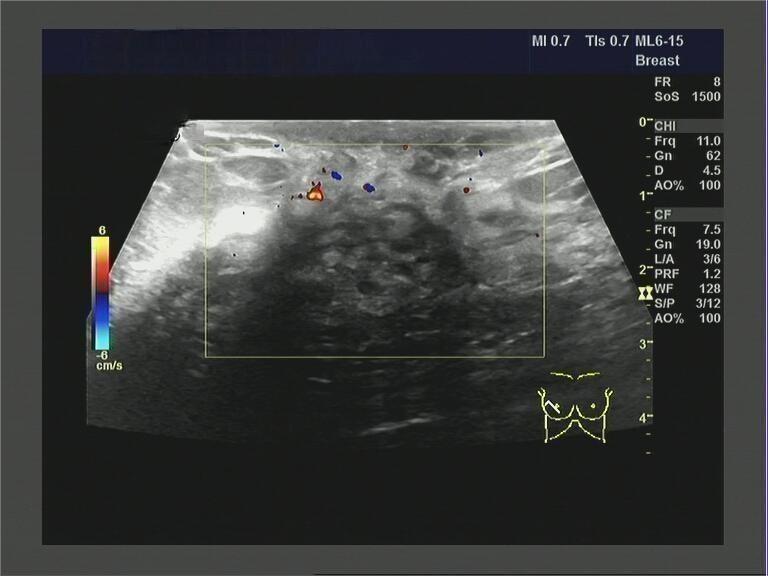

Supplement: S2 Data — Representative sonographic images of breast non-mass lesions. (ZIP) [file pone.0278299.s002.zip › Supplementary data 2/46/e.jpg]

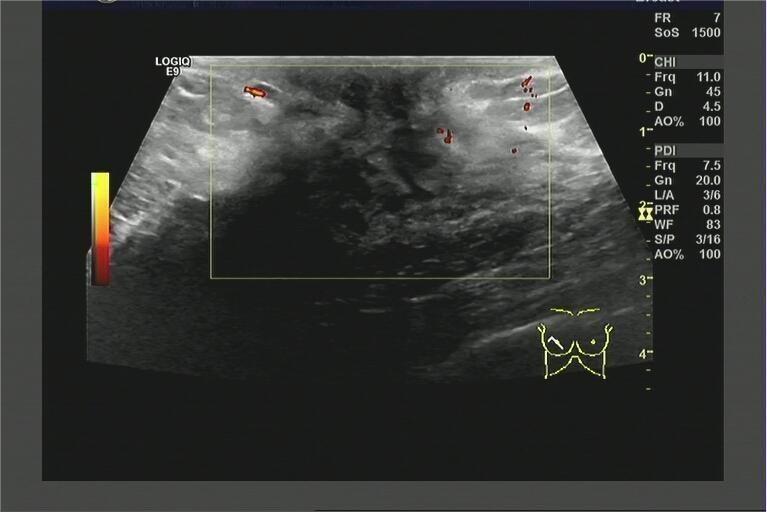

Supplement: S2 Data — Representative sonographic images of breast non-mass lesions. (ZIP) [file pone.0278299.s002.zip › Supplementary data 2/46/f.jpg]

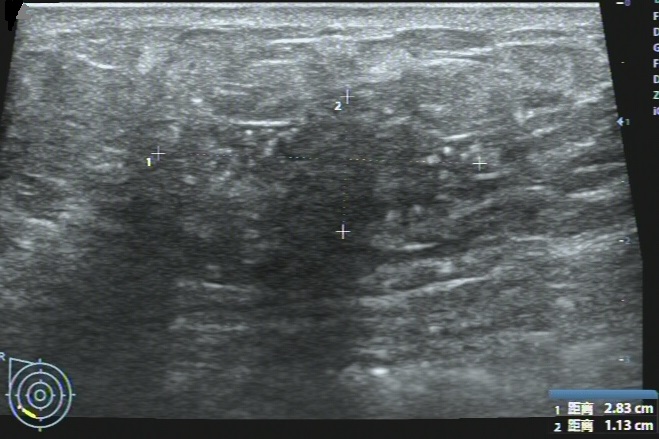

Supplement: S2 Data — Representative sonographic images of breast non-mass lesions. (ZIP) [file pone.0278299.s002.zip › Supplementary data 2/47/a.jpg]

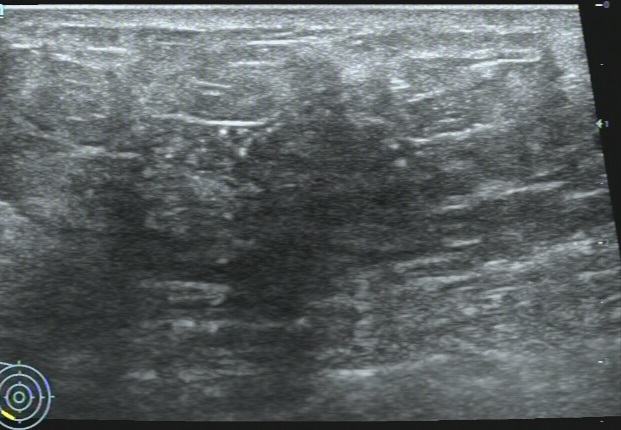

Supplement: S2 Data — Representative sonographic images of breast non-mass lesions. (ZIP) [file pone.0278299.s002.zip › Supplementary data 2/47/b.jpg]

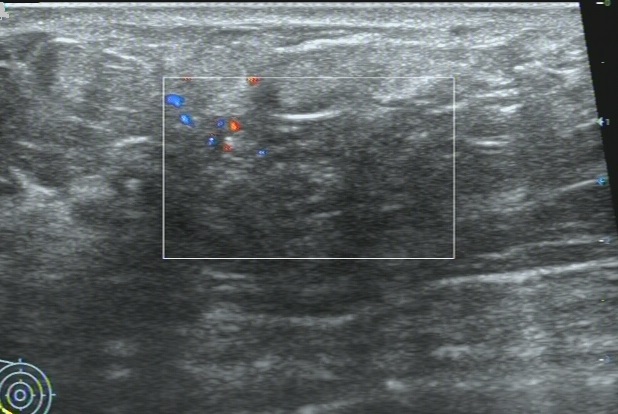

Supplement: S2 Data — Representative sonographic images of breast non-mass lesions. (ZIP) [file pone.0278299.s002.zip › Supplementary data 2/47/c.jpg]

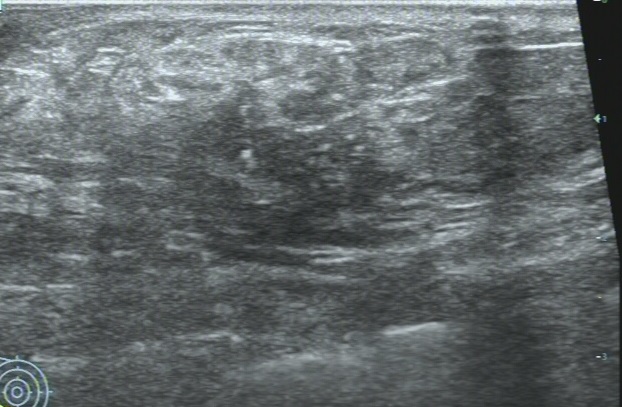

Supplement: S2 Data — Representative sonographic images of breast non-mass lesions. (ZIP) [file pone.0278299.s002.zip › Supplementary data 2/47/d.jpg]

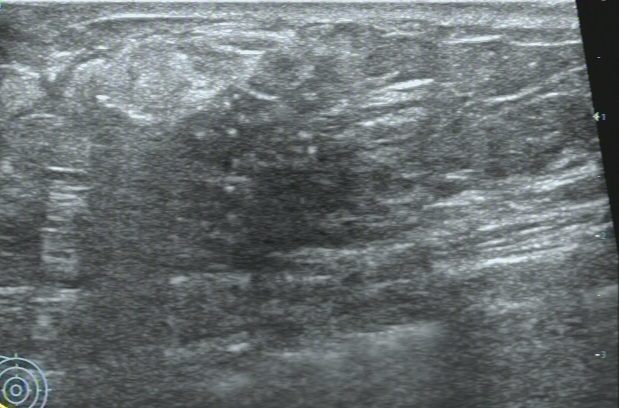

Supplement: S2 Data — Representative sonographic images of breast non-mass lesions. (ZIP) [file pone.0278299.s002.zip › Supplementary data 2/47/e.jpg]

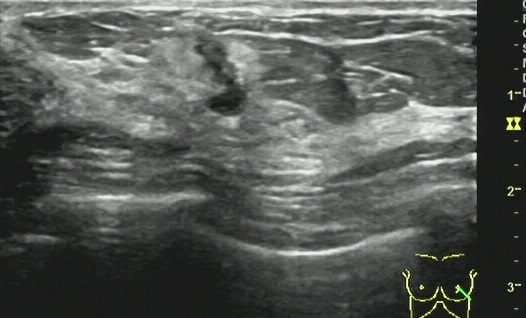

Supplement: S2 Data — Representative sonographic images of breast non-mass lesions. (ZIP) [file pone.0278299.s002.zip › Supplementary data 2/48/a.jpg]

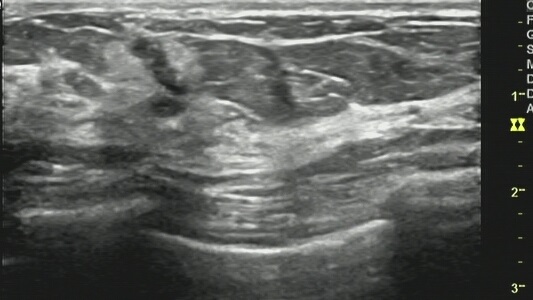

Supplement: S2 Data — Representative sonographic images of breast non-mass lesions. (ZIP) [file pone.0278299.s002.zip › Supplementary data 2/48/b.jpg]

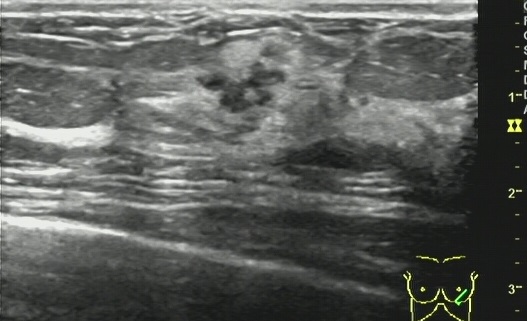

Supplement: S2 Data — Representative sonographic images of breast non-mass lesions. (ZIP) [file pone.0278299.s002.zip › Supplementary data 2/48/c.jpg]

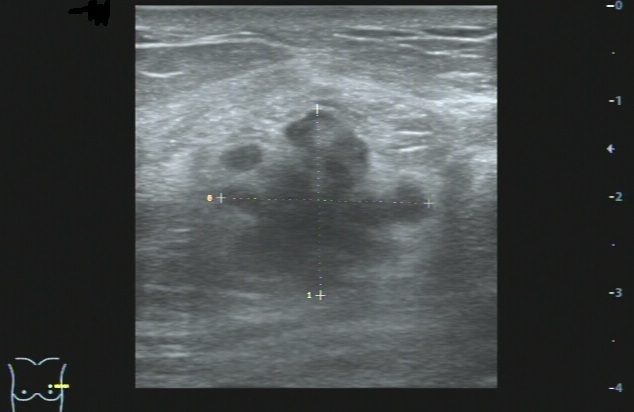

Supplement: S2 Data — Representative sonographic images of breast non-mass lesions. (ZIP) [file pone.0278299.s002.zip › Supplementary data 2/49/a.jpg]

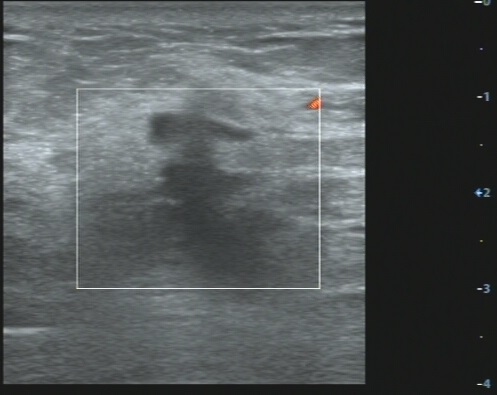

Supplement: S2 Data — Representative sonographic images of breast non-mass lesions. (ZIP) [file pone.0278299.s002.zip › Supplementary data 2/49/b.jpg]

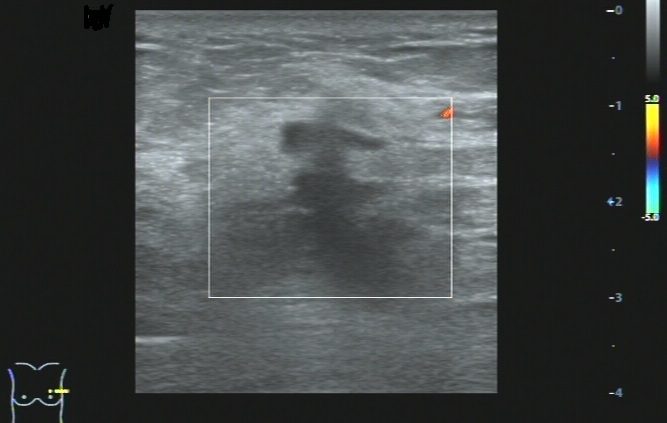

Supplement: S2 Data — Representative sonographic images of breast non-mass lesions. (ZIP) [file pone.0278299.s002.zip › Supplementary data 2/49/c.jpg]

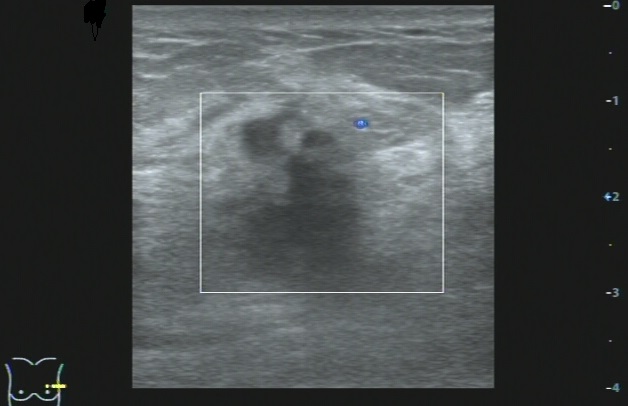

Supplement: S2 Data — Representative sonographic images of breast non-mass lesions. (ZIP) [file pone.0278299.s002.zip › Supplementary data 2/49/d.jpg]

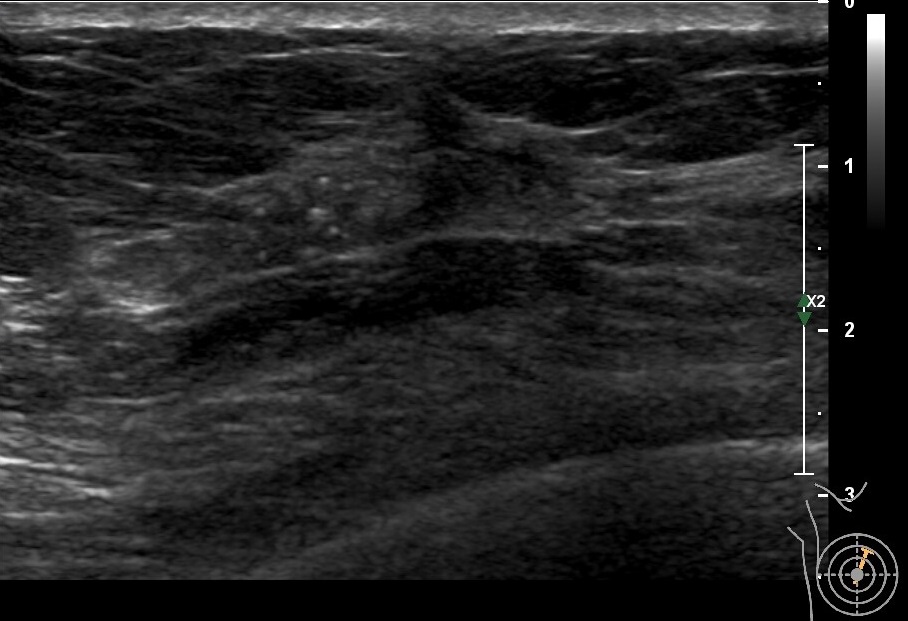

Supplement: S2 Data — Representative sonographic images of breast non-mass lesions. (ZIP) [file pone.0278299.s002.zip › Supplementary data 2/5/a.jpg]

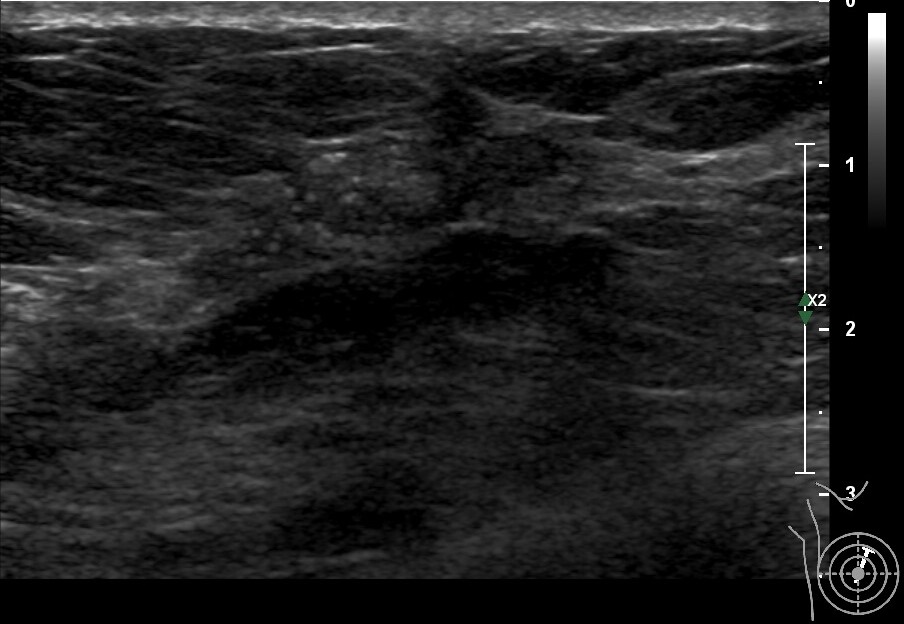

Supplement: S2 Data — Representative sonographic images of breast non-mass lesions. (ZIP) [file pone.0278299.s002.zip › Supplementary data 2/5/b.jpg]

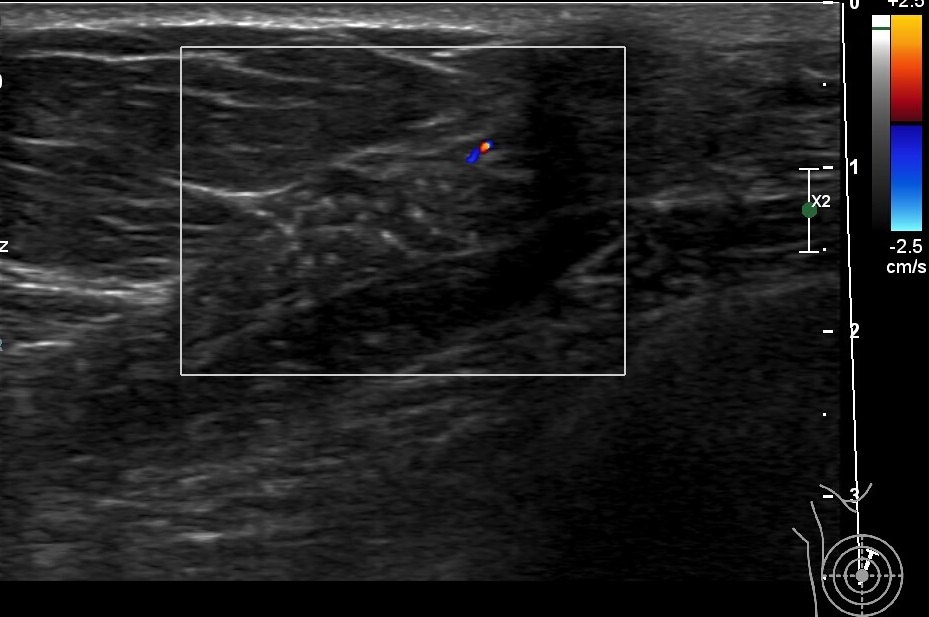

Supplement: S2 Data — Representative sonographic images of breast non-mass lesions. (ZIP) [file pone.0278299.s002.zip › Supplementary data 2/5/c.jpg]

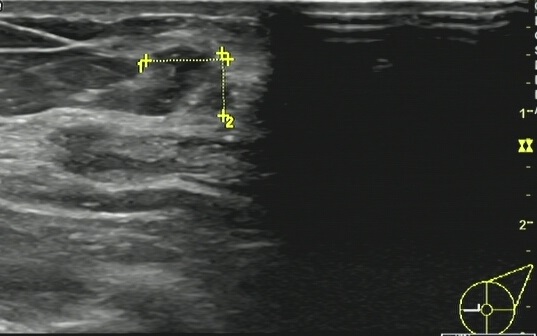

Supplement: S2 Data — Representative sonographic images of breast non-mass lesions. (ZIP) [file pone.0278299.s002.zip › Supplementary data 2/50/a.jpg]

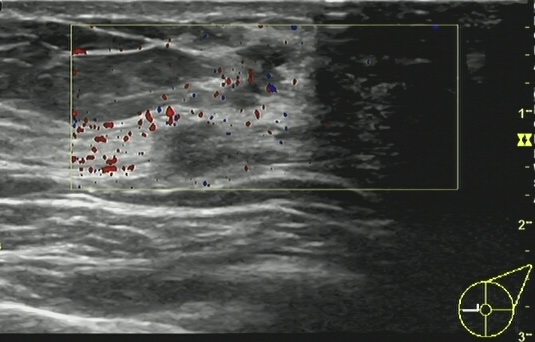

Supplement: S2 Data — Representative sonographic images of breast non-mass lesions. (ZIP) [file pone.0278299.s002.zip › Supplementary data 2/50/b.jpg]

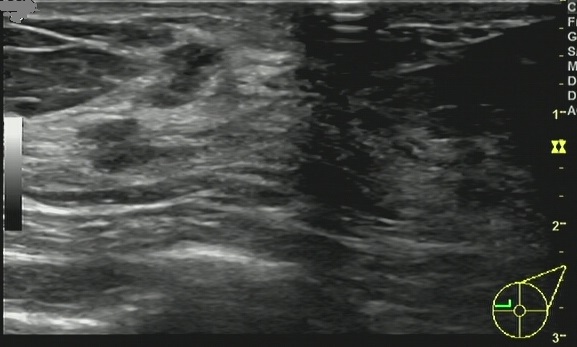

Supplement: S2 Data — Representative sonographic images of breast non-mass lesions. (ZIP) [file pone.0278299.s002.zip › Supplementary data 2/50/c.jpg]

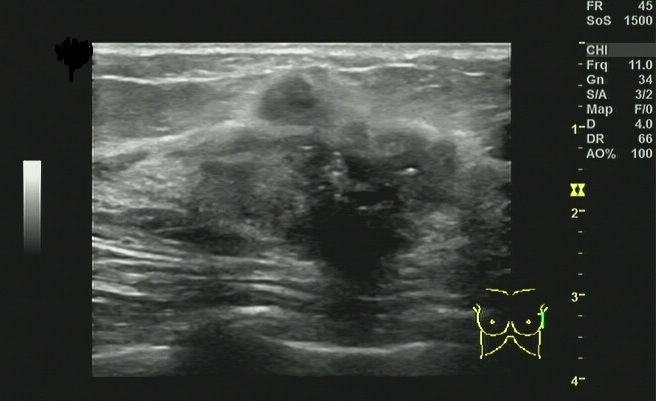

Supplement: S2 Data — Representative sonographic images of breast non-mass lesions. (ZIP) [file pone.0278299.s002.zip › Supplementary data 2/51/a.jpg]

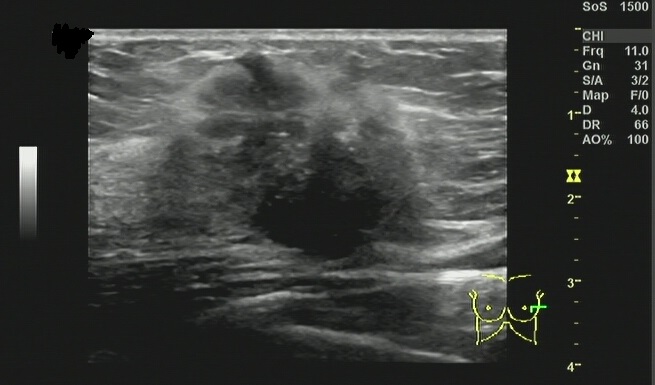

Supplement: S2 Data — Representative sonographic images of breast non-mass lesions. (ZIP) [file pone.0278299.s002.zip › Supplementary data 2/51/b.jpg]

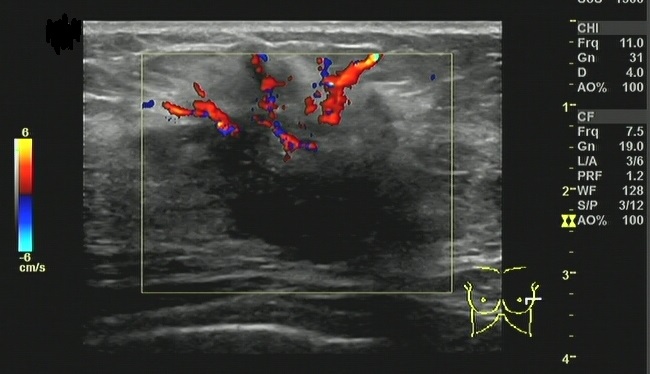

Supplement: S2 Data — Representative sonographic images of breast non-mass lesions. (ZIP) [file pone.0278299.s002.zip › Supplementary data 2/51/c.jpg]

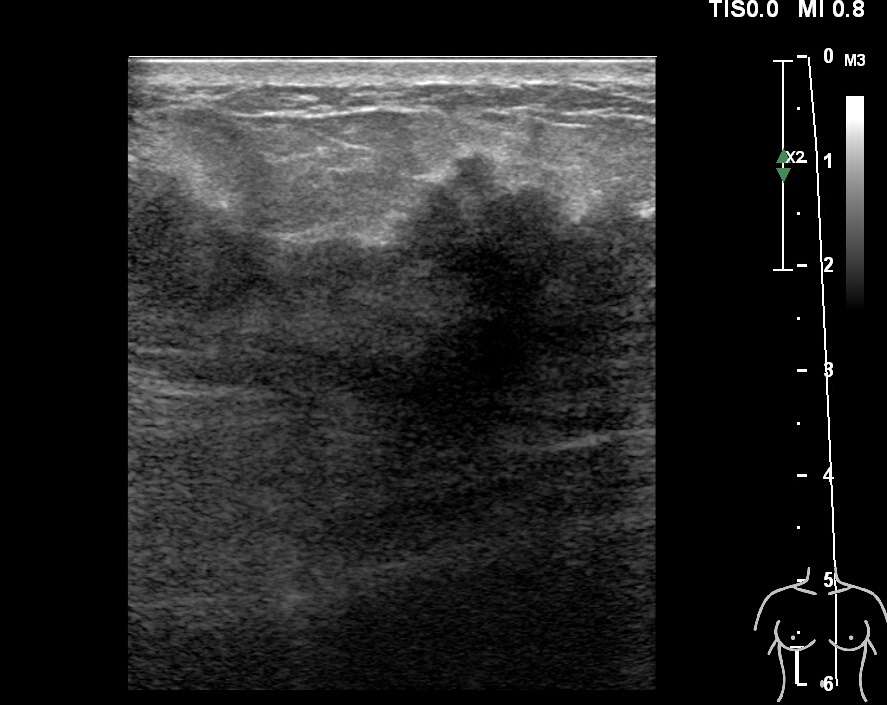

Supplement: S2 Data — Representative sonographic images of breast non-mass lesions. (ZIP) [file pone.0278299.s002.zip › Supplementary data 2/52/a.jpg]

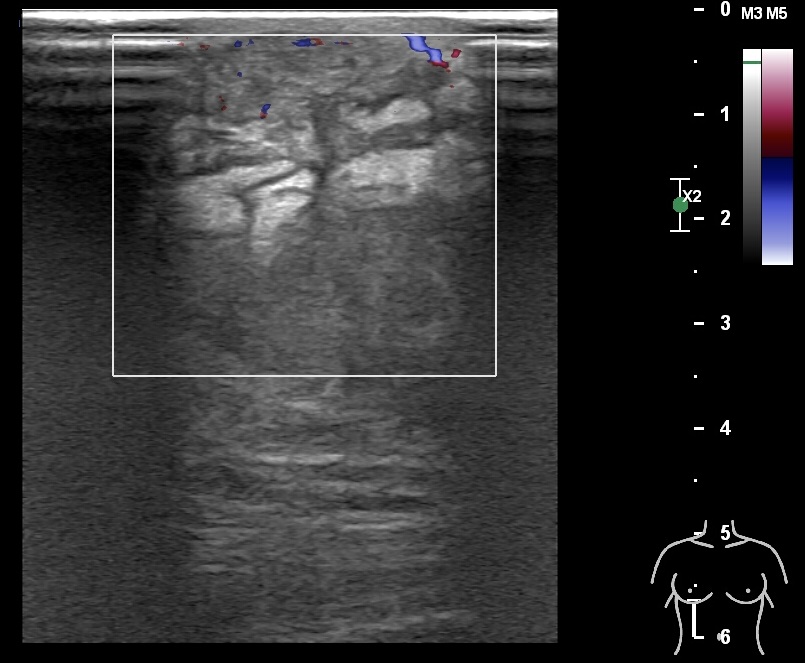

Supplement: S2 Data — Representative sonographic images of breast non-mass lesions. (ZIP) [file pone.0278299.s002.zip › Supplementary data 2/52/b.jpg]

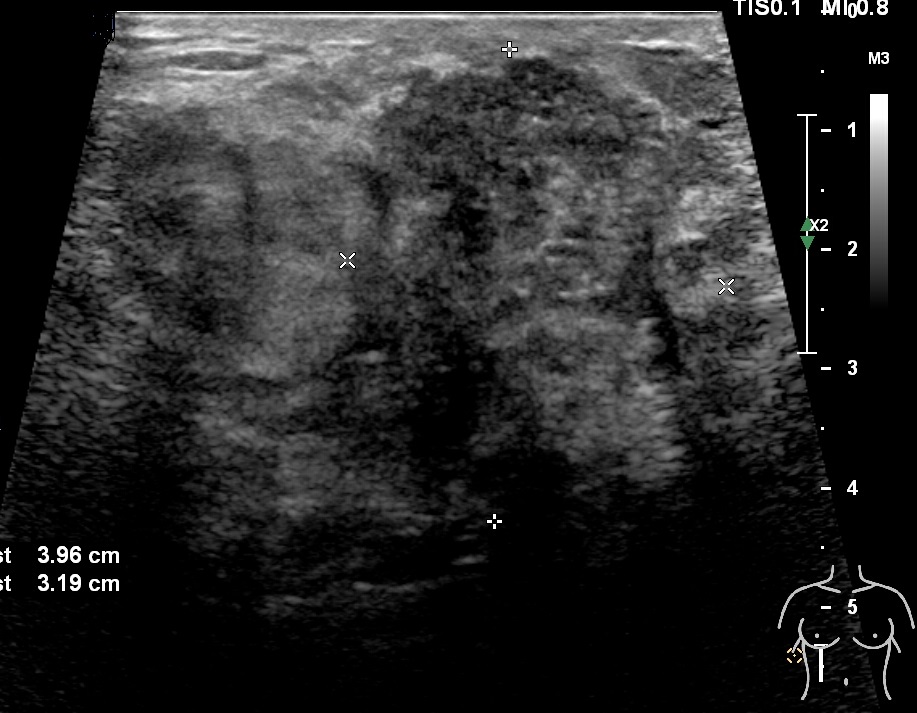

Supplement: S2 Data — Representative sonographic images of breast non-mass lesions. (ZIP) [file pone.0278299.s002.zip › Supplementary data 2/52/c.jpg]

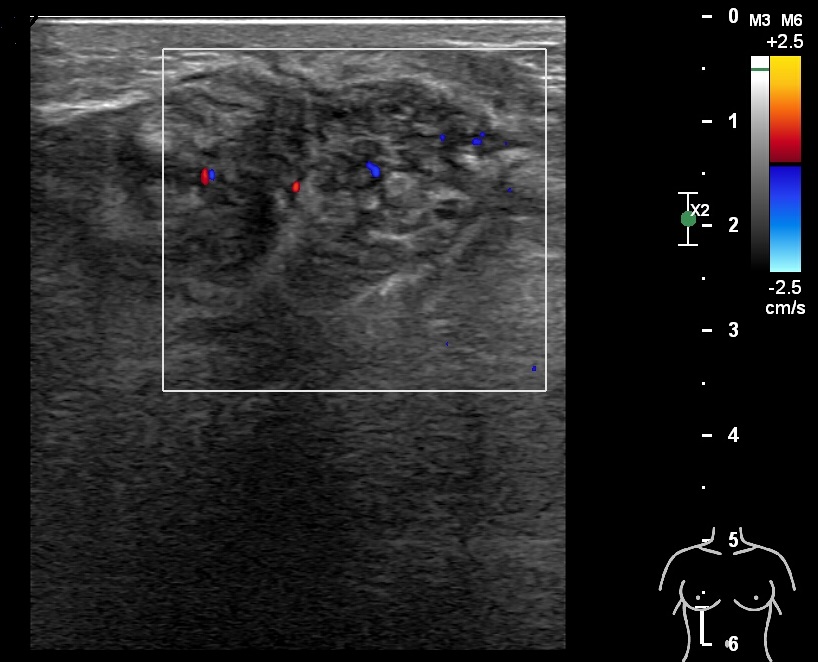

Supplement: S2 Data — Representative sonographic images of breast non-mass lesions. (ZIP) [file pone.0278299.s002.zip › Supplementary data 2/52/d.jpg]

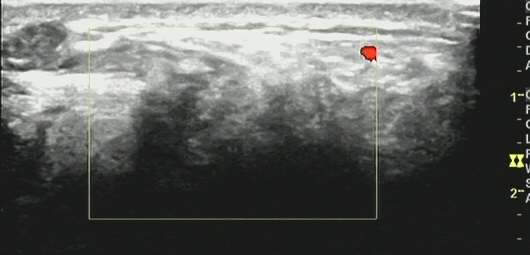

Supplement: S2 Data — Representative sonographic images of breast non-mass lesions. (ZIP) [file pone.0278299.s002.zip › Supplementary data 2/53/a.jpg]

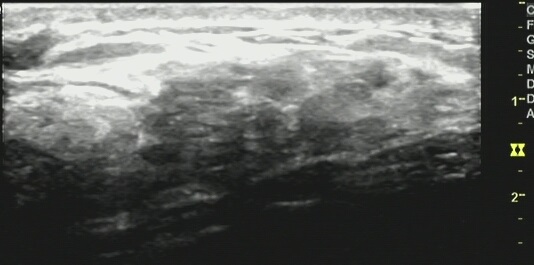

Supplement: S2 Data — Representative sonographic images of breast non-mass lesions. (ZIP) [file pone.0278299.s002.zip › Supplementary data 2/53/b.jpg]

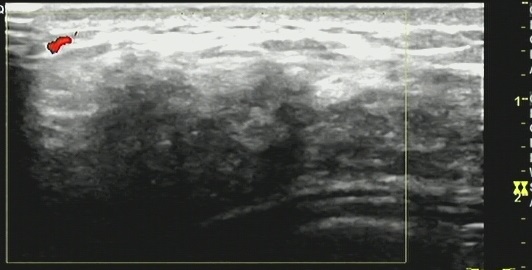

Supplement: S2 Data — Representative sonographic images of breast non-mass lesions. (ZIP) [file pone.0278299.s002.zip › Supplementary data 2/53/c.jpg]

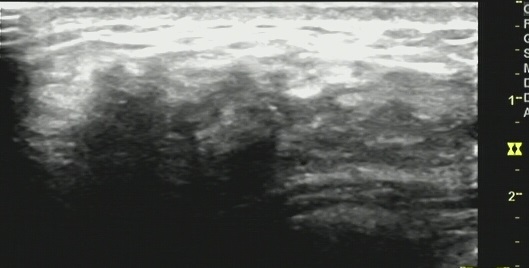

Supplement: S2 Data — Representative sonographic images of breast non-mass lesions. (ZIP) [file pone.0278299.s002.zip › Supplementary data 2/53/d.jpg]

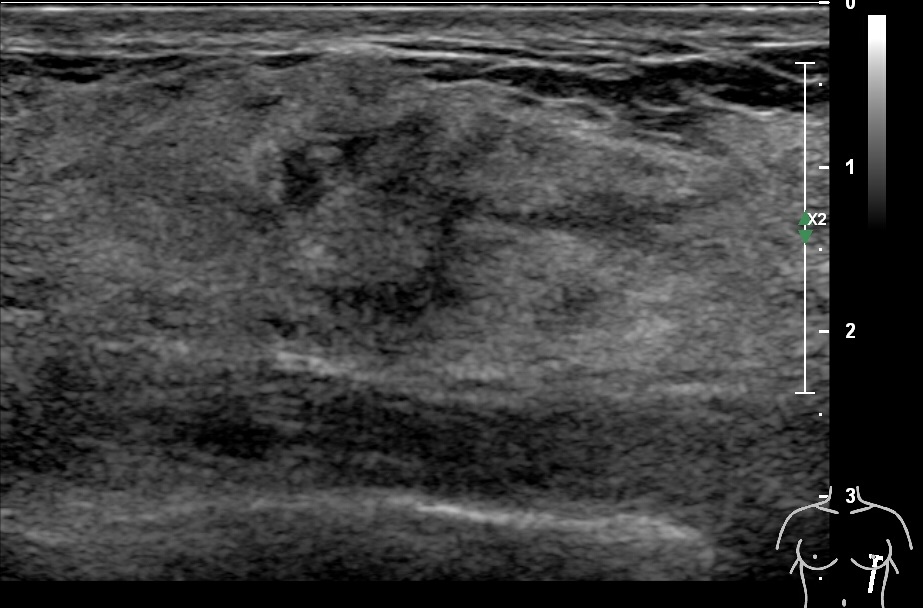

Supplement: S2 Data — Representative sonographic images of breast non-mass lesions. (ZIP) [file pone.0278299.s002.zip › Supplementary data 2/54/a.jpg]

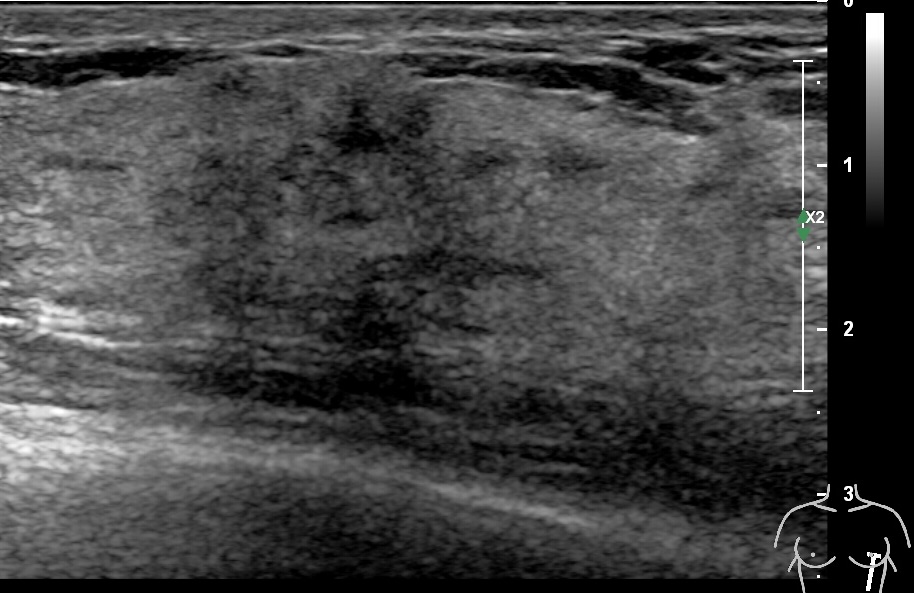

Supplement: S2 Data — Representative sonographic images of breast non-mass lesions. (ZIP) [file pone.0278299.s002.zip › Supplementary data 2/54/b.jpg]

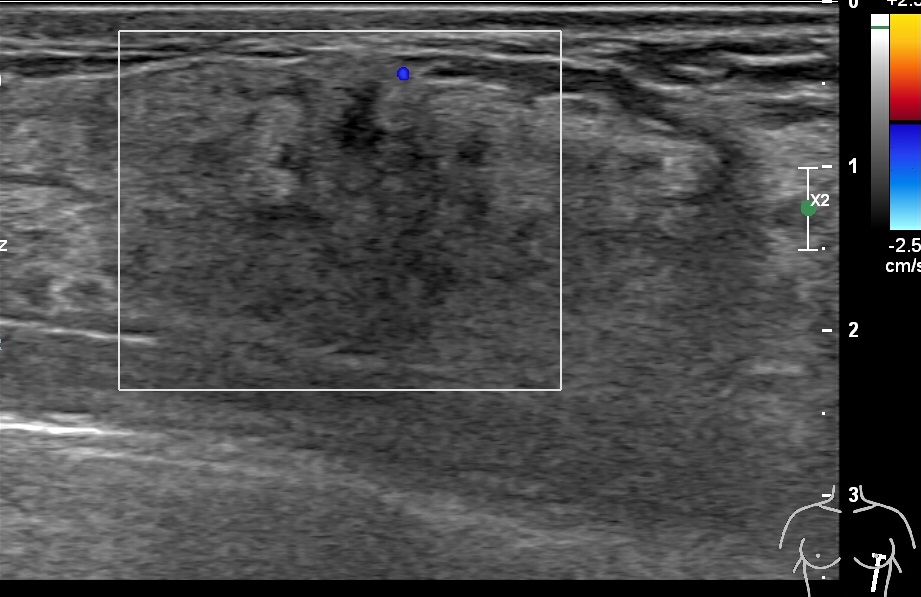

Supplement: S2 Data — Representative sonographic images of breast non-mass lesions. (ZIP) [file pone.0278299.s002.zip › Supplementary data 2/54/c.jpg]

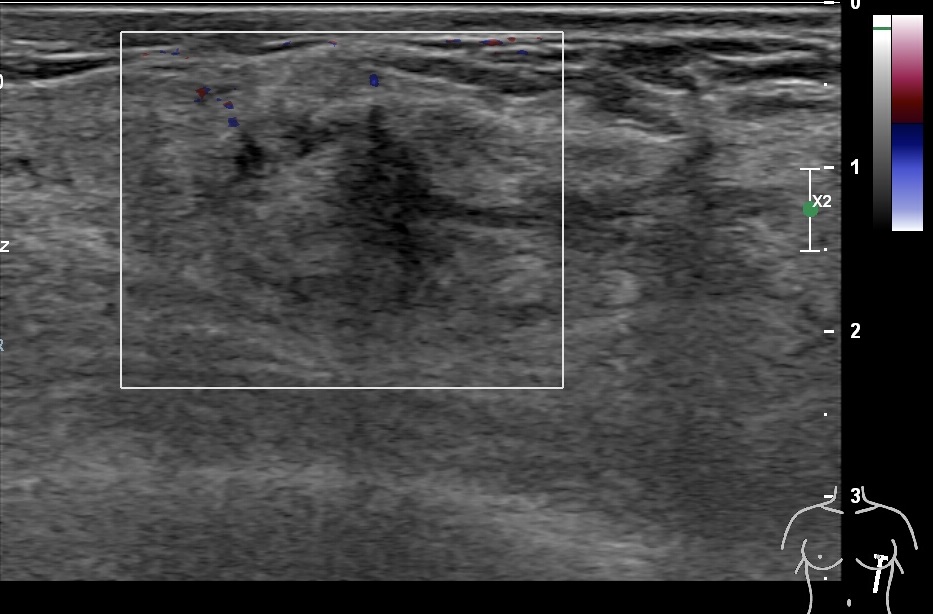

Supplement: S2 Data — Representative sonographic images of breast non-mass lesions. (ZIP) [file pone.0278299.s002.zip › Supplementary data 2/54/d.jpg]

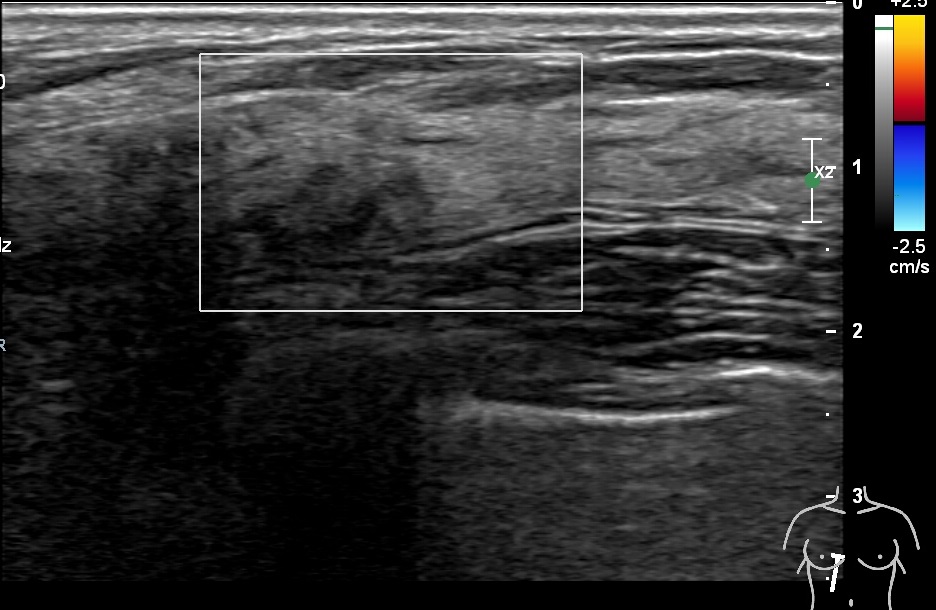

Supplement: S2 Data — Representative sonographic images of breast non-mass lesions. (ZIP) [file pone.0278299.s002.zip › Supplementary data 2/54/e.jpg]

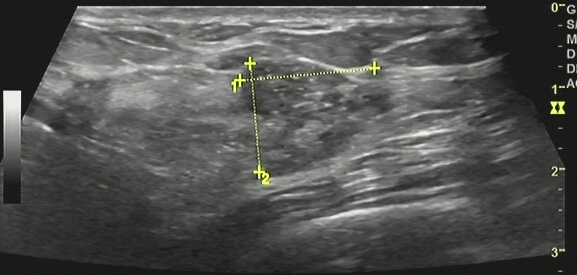

Supplement: S2 Data — Representative sonographic images of breast non-mass lesions. (ZIP) [file pone.0278299.s002.zip › Supplementary data 2/55/a.jpg]

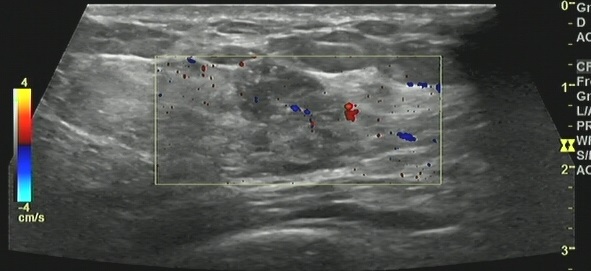

Supplement: S2 Data — Representative sonographic images of breast non-mass lesions. (ZIP) [file pone.0278299.s002.zip › Supplementary data 2/55/b.jpg]

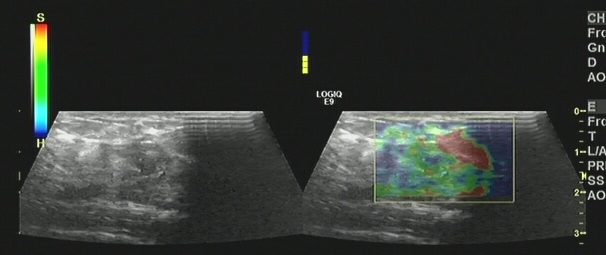

Supplement: S2 Data — Representative sonographic images of breast non-mass lesions. (ZIP) [file pone.0278299.s002.zip › Supplementary data 2/55/c.jpg]

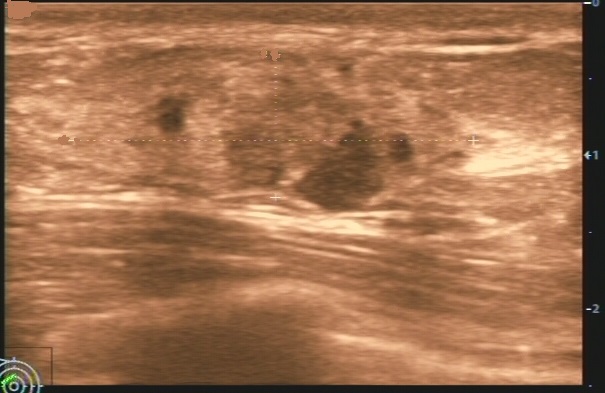

Supplement: S2 Data — Representative sonographic images of breast non-mass lesions. (ZIP) [file pone.0278299.s002.zip › Supplementary data 2/56/a.jpg]

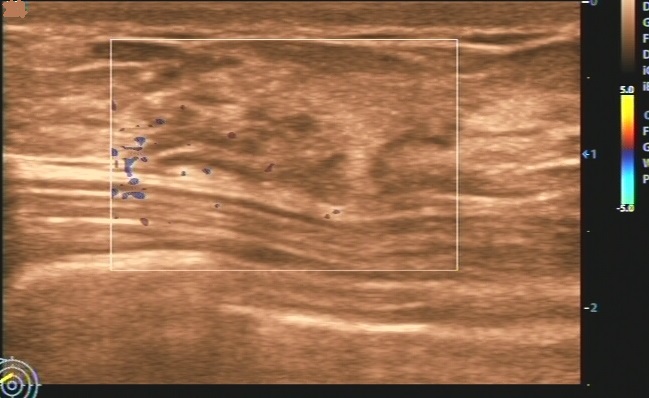

Supplement: S2 Data — Representative sonographic images of breast non-mass lesions. (ZIP) [file pone.0278299.s002.zip › Supplementary data 2/56/b.jpg]

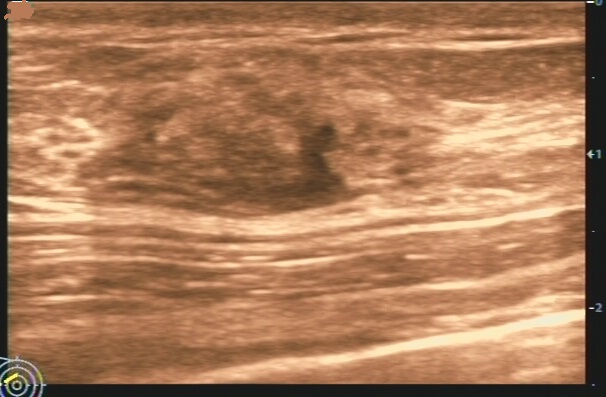

Supplement: S2 Data — Representative sonographic images of breast non-mass lesions. (ZIP) [file pone.0278299.s002.zip › Supplementary data 2/56/c.jpg]

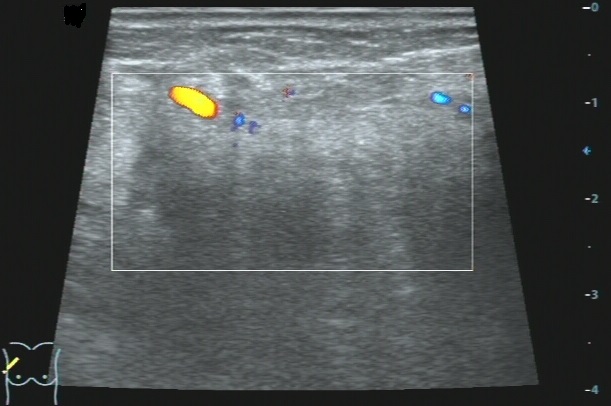

Supplement: S2 Data — Representative sonographic images of breast non-mass lesions. (ZIP) [file pone.0278299.s002.zip › Supplementary data 2/57/a.jpg]

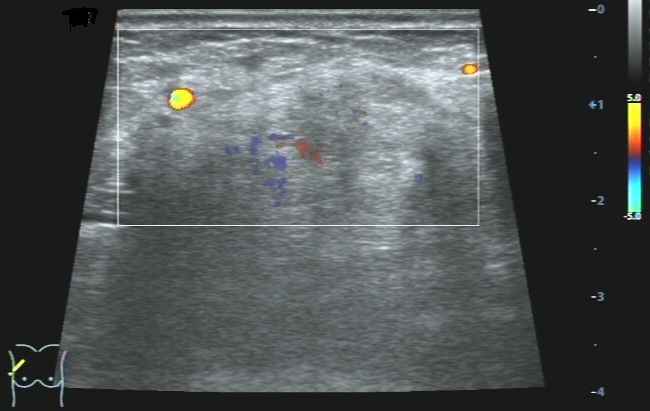

Supplement: S2 Data — Representative sonographic images of breast non-mass lesions. (ZIP) [file pone.0278299.s002.zip › Supplementary data 2/57/b.jpg]

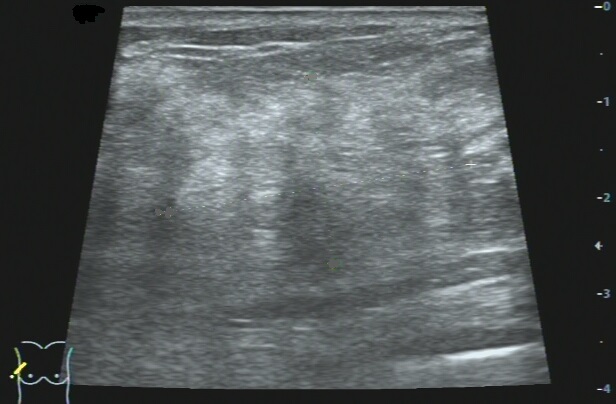

Supplement: S2 Data — Representative sonographic images of breast non-mass lesions. (ZIP) [file pone.0278299.s002.zip › Supplementary data 2/57/c.jpg]

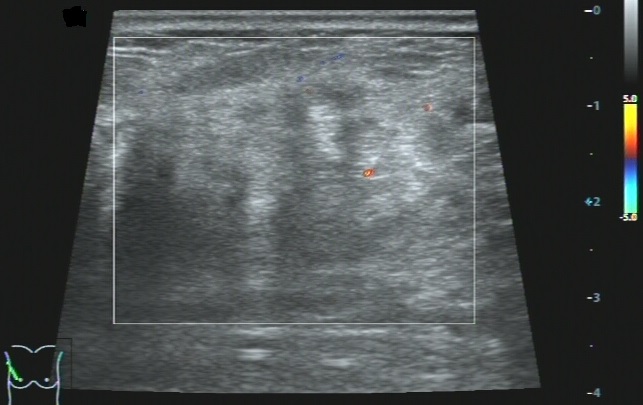

Supplement: S2 Data — Representative sonographic images of breast non-mass lesions. (ZIP) [file pone.0278299.s002.zip › Supplementary data 2/57/d.jpg]

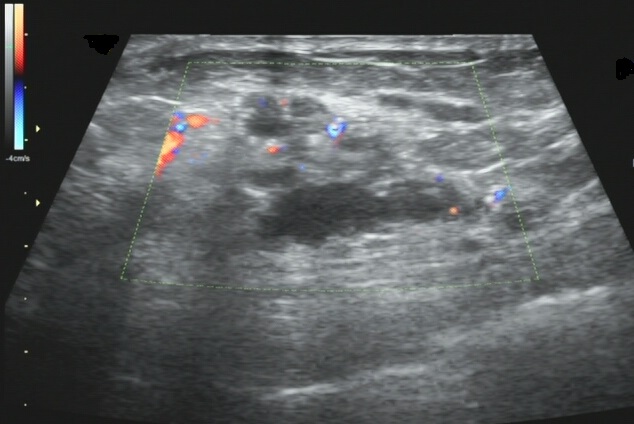

Supplement: S2 Data — Representative sonographic images of breast non-mass lesions. (ZIP) [file pone.0278299.s002.zip › Supplementary data 2/58/a.jpg]

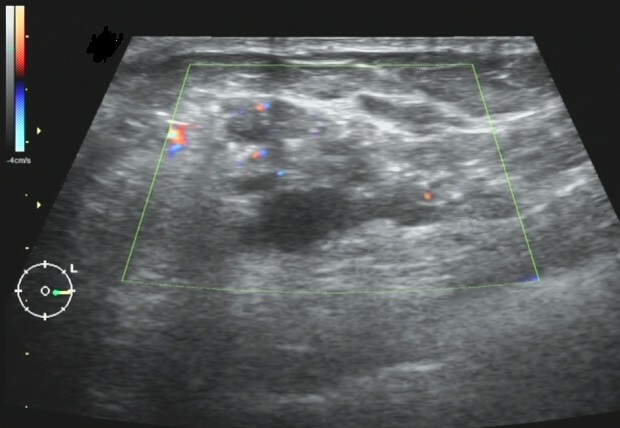

Supplement: S2 Data — Representative sonographic images of breast non-mass lesions. (ZIP) [file pone.0278299.s002.zip › Supplementary data 2/58/b.jpg]
