# Supplementary figures and images for: Ultrasound classification of non-mass breast lesions following BI-RADS presents high positive predictive value (part 1 of 2)
Source: PLoS One. 2022 Nov 30;17(11):e0278299. doi: 10.1371/journal.pone.0278299 (PMC9710769; doi:10.1371/journal.pone.0278299)

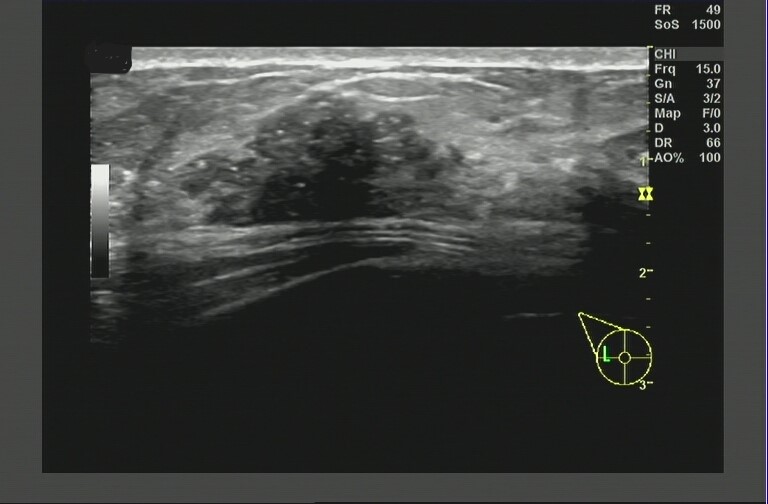

Supplement: S2 Data — Representative sonographic images of breast non-mass lesions. (ZIP) [file pone.0278299.s002.zip › Supplementary data 2/1/a.jpg]

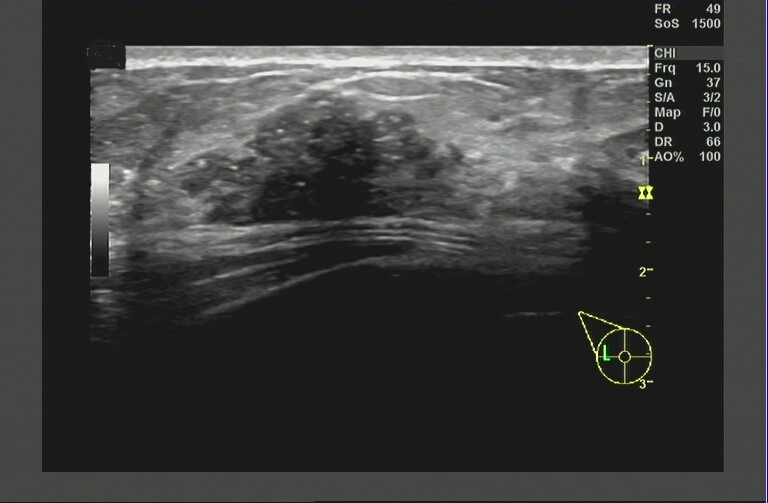

Supplement: S2 Data — Representative sonographic images of breast non-mass lesions. (ZIP) [file pone.0278299.s002.zip › Supplementary data 2/1/b.jpg]

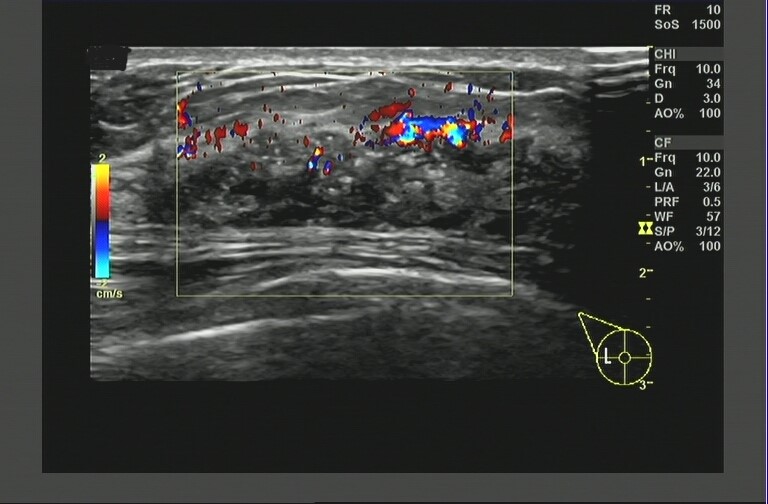

Supplement: S2 Data — Representative sonographic images of breast non-mass lesions. (ZIP) [file pone.0278299.s002.zip › Supplementary data 2/1/c.jpg]

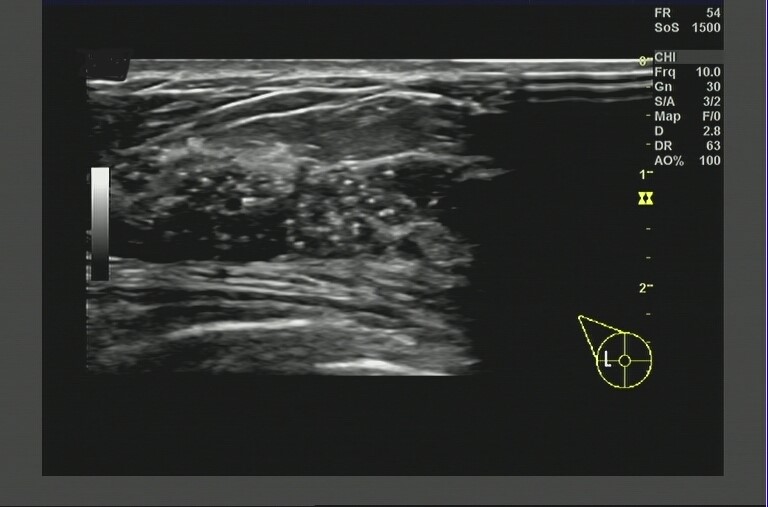

Supplement: S2 Data — Representative sonographic images of breast non-mass lesions. (ZIP) [file pone.0278299.s002.zip › Supplementary data 2/1/d.jpg]

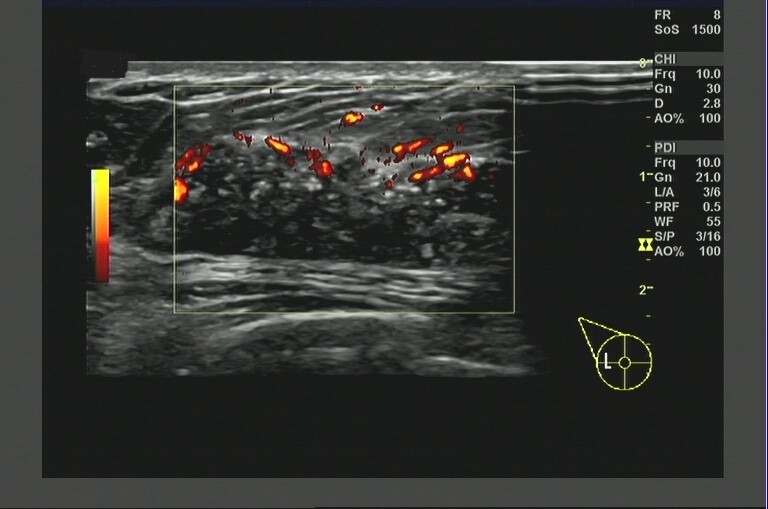

Supplement: S2 Data — Representative sonographic images of breast non-mass lesions. (ZIP) [file pone.0278299.s002.zip › Supplementary data 2/1/e.jpg]

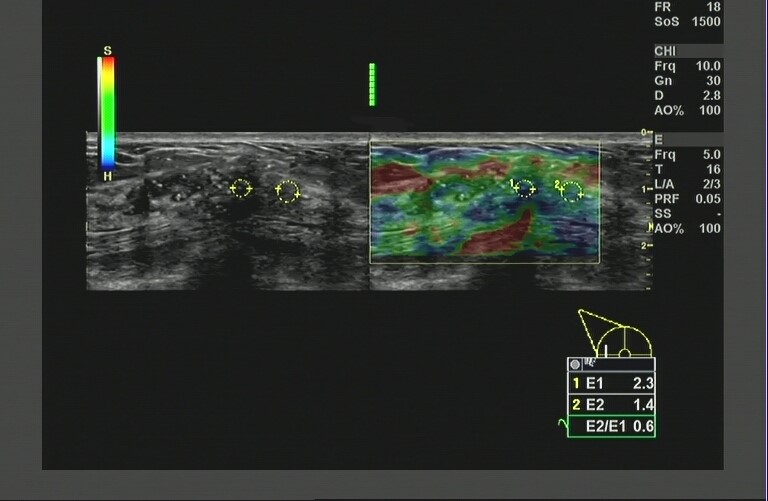

Supplement: S2 Data — Representative sonographic images of breast non-mass lesions. (ZIP) [file pone.0278299.s002.zip › Supplementary data 2/1/f.jpg]

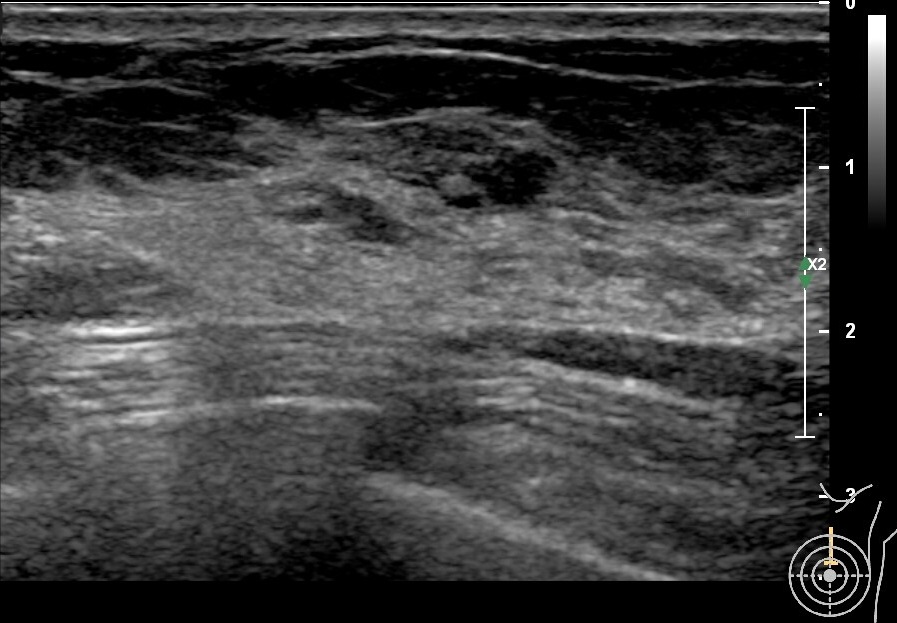

Supplement: S2 Data — Representative sonographic images of breast non-mass lesions. (ZIP) [file pone.0278299.s002.zip › Supplementary data 2/10/a.jpg]

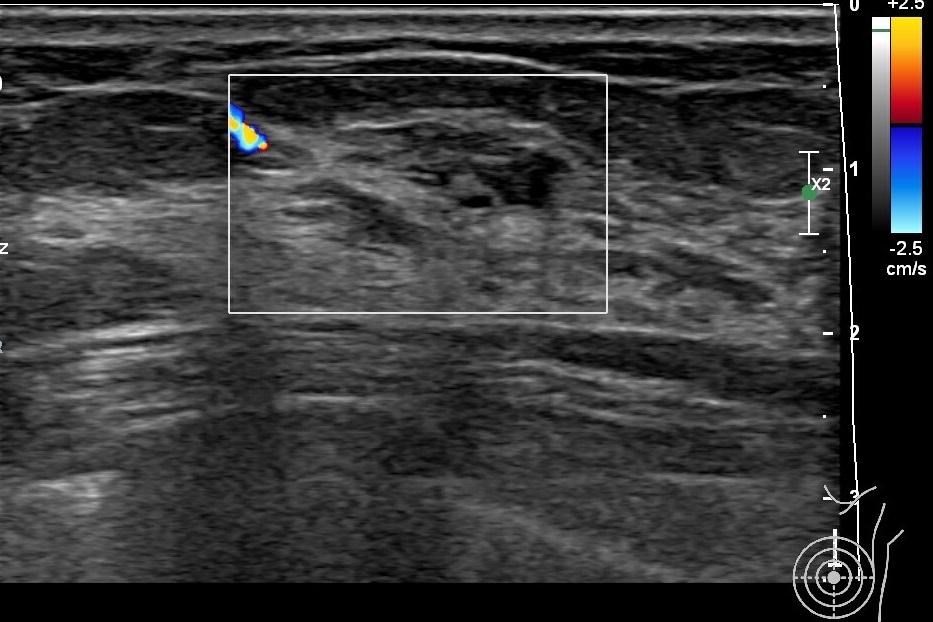

Supplement: S2 Data — Representative sonographic images of breast non-mass lesions. (ZIP) [file pone.0278299.s002.zip › Supplementary data 2/10/b.jpg]

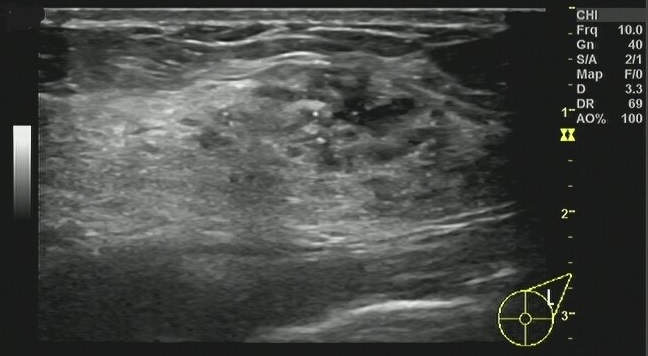

Supplement: S2 Data — Representative sonographic images of breast non-mass lesions. (ZIP) [file pone.0278299.s002.zip › Supplementary data 2/11/a.jpg]

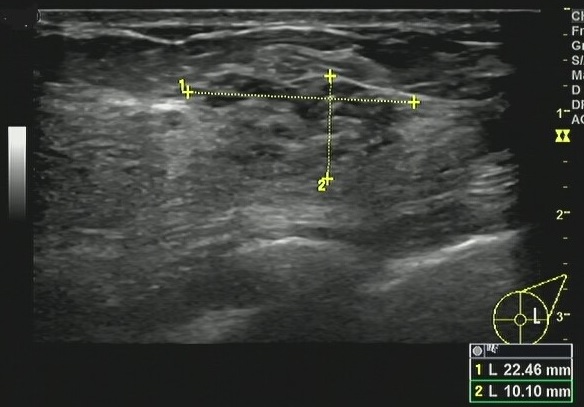

Supplement: S2 Data — Representative sonographic images of breast non-mass lesions. (ZIP) [file pone.0278299.s002.zip › Supplementary data 2/11/b.jpg]

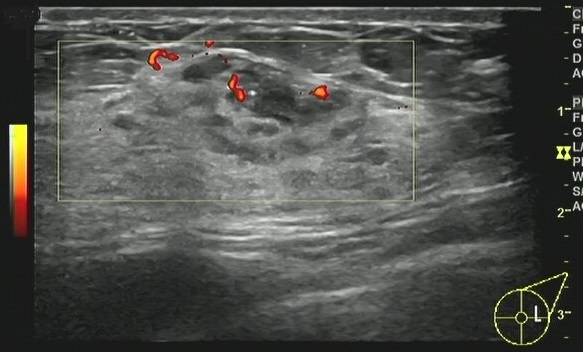

Supplement: S2 Data — Representative sonographic images of breast non-mass lesions. (ZIP) [file pone.0278299.s002.zip › Supplementary data 2/11/c.jpg]

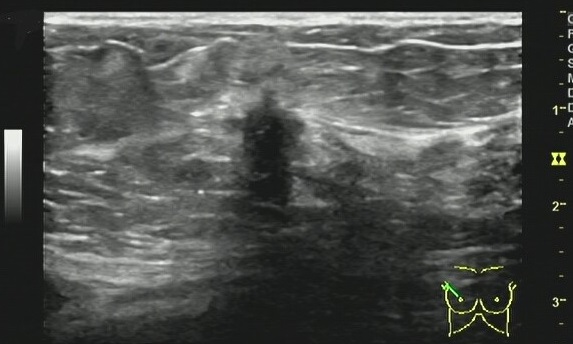

Supplement: S2 Data — Representative sonographic images of breast non-mass lesions. (ZIP) [file pone.0278299.s002.zip › Supplementary data 2/12/a.jpg]

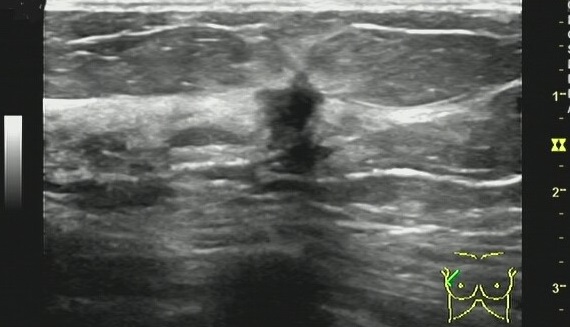

Supplement: S2 Data — Representative sonographic images of breast non-mass lesions. (ZIP) [file pone.0278299.s002.zip › Supplementary data 2/12/b.jpg]

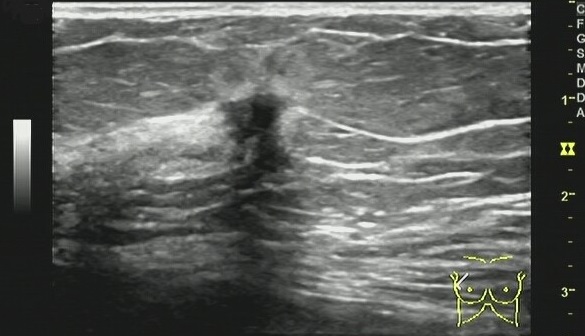

Supplement: S2 Data — Representative sonographic images of breast non-mass lesions. (ZIP) [file pone.0278299.s002.zip › Supplementary data 2/12/c.jpg]

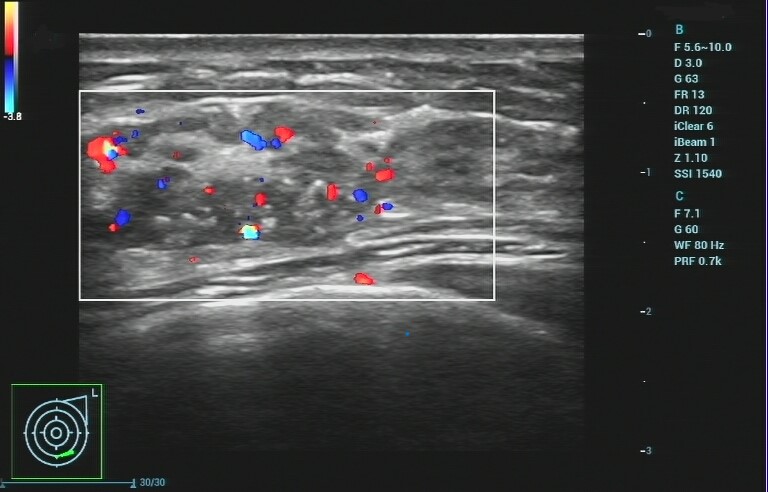

Supplement: S2 Data — Representative sonographic images of breast non-mass lesions. (ZIP) [file pone.0278299.s002.zip › Supplementary data 2/13/a.jpg]

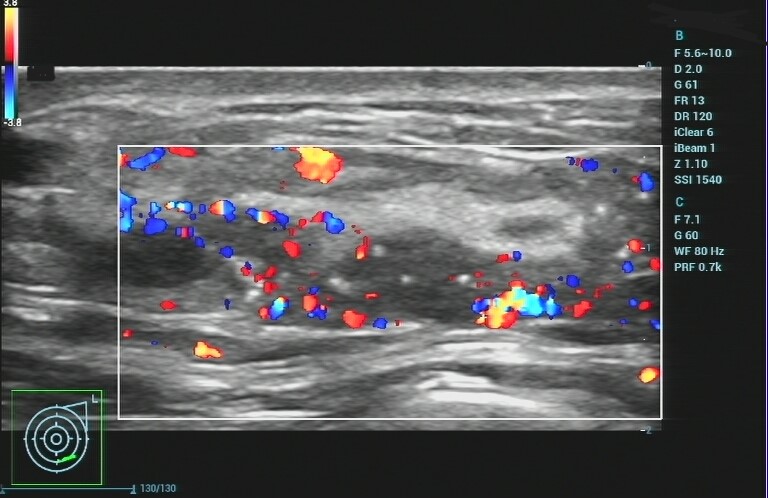

Supplement: S2 Data — Representative sonographic images of breast non-mass lesions. (ZIP) [file pone.0278299.s002.zip › Supplementary data 2/13/b.jpg]

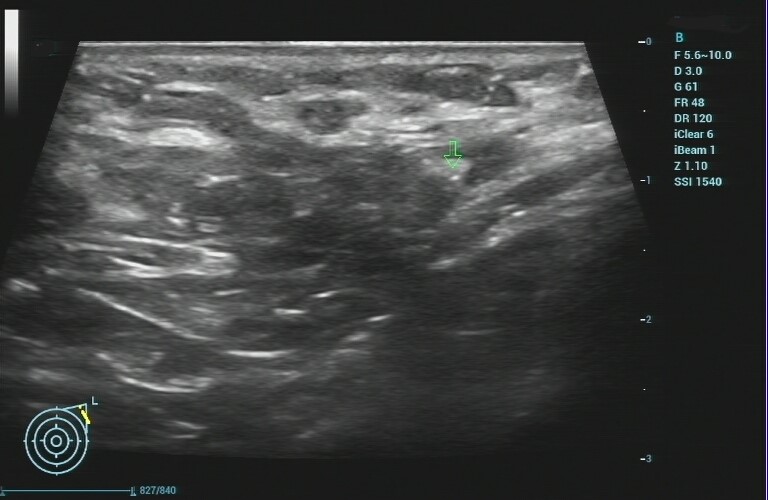

Supplement: S2 Data — Representative sonographic images of breast non-mass lesions. (ZIP) [file pone.0278299.s002.zip › Supplementary data 2/13/c.jpg]

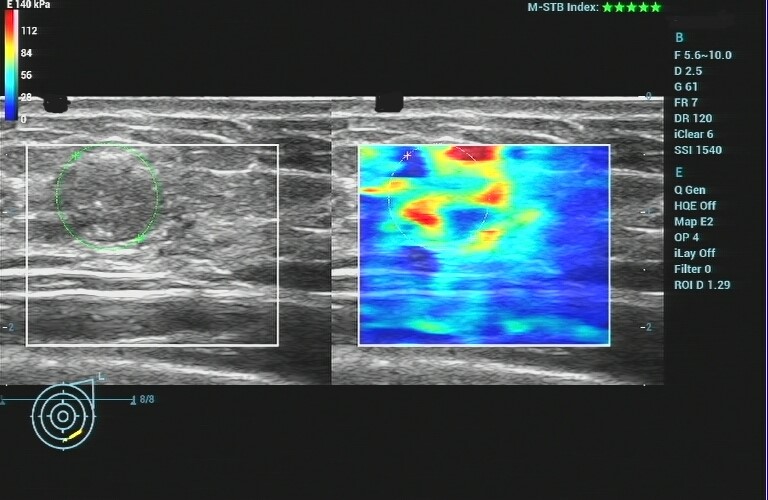

Supplement: S2 Data — Representative sonographic images of breast non-mass lesions. (ZIP) [file pone.0278299.s002.zip › Supplementary data 2/13/d.jpg]

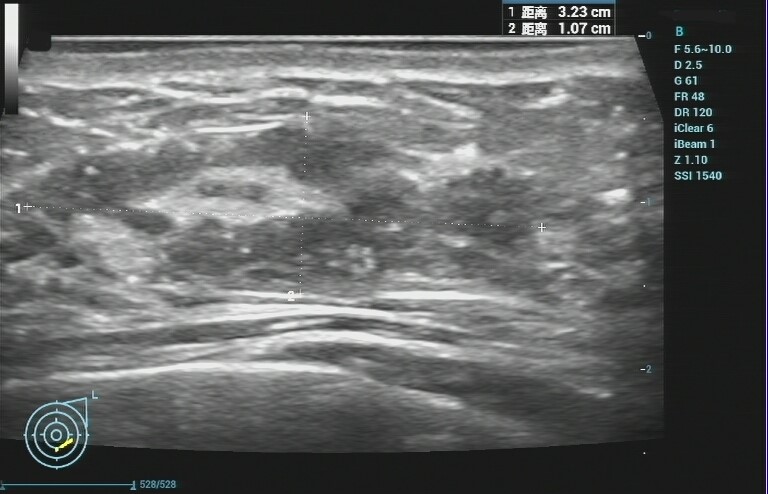

Supplement: S2 Data — Representative sonographic images of breast non-mass lesions. (ZIP) [file pone.0278299.s002.zip › Supplementary data 2/13/e.jpg]

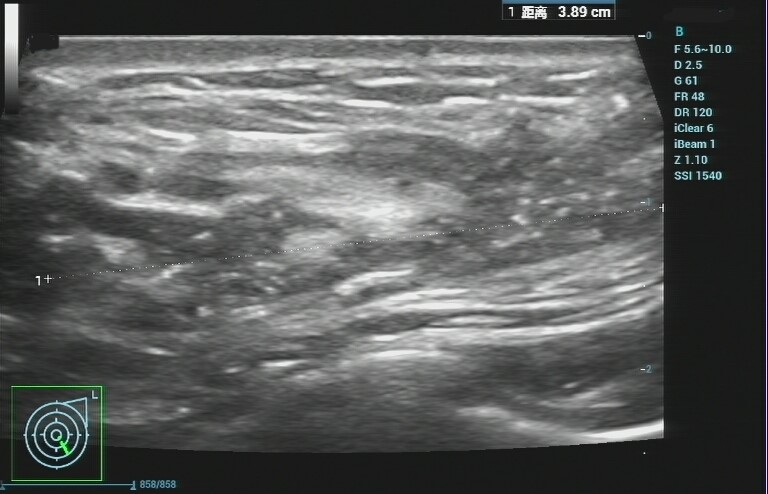

Supplement: S2 Data — Representative sonographic images of breast non-mass lesions. (ZIP) [file pone.0278299.s002.zip › Supplementary data 2/13/f.jpg]

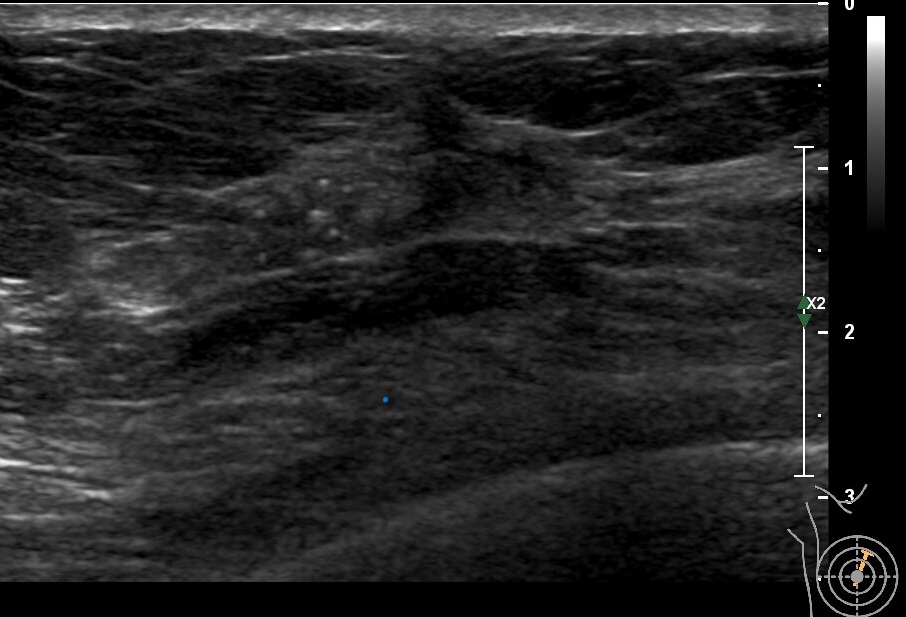

Supplement: S2 Data — Representative sonographic images of breast non-mass lesions. (ZIP) [file pone.0278299.s002.zip › Supplementary data 2/14/a.jpg]

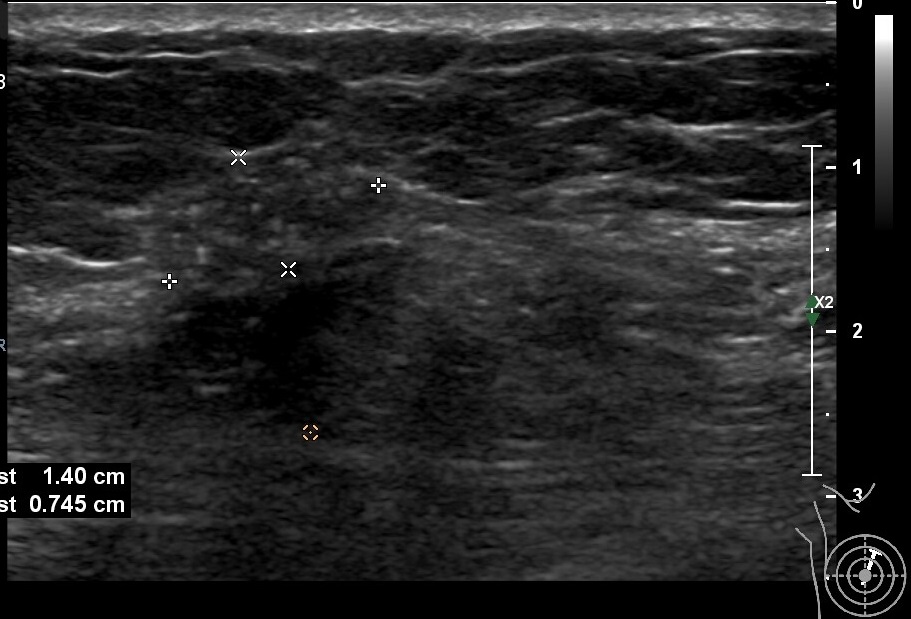

Supplement: S2 Data — Representative sonographic images of breast non-mass lesions. (ZIP) [file pone.0278299.s002.zip › Supplementary data 2/14/b.jpg]

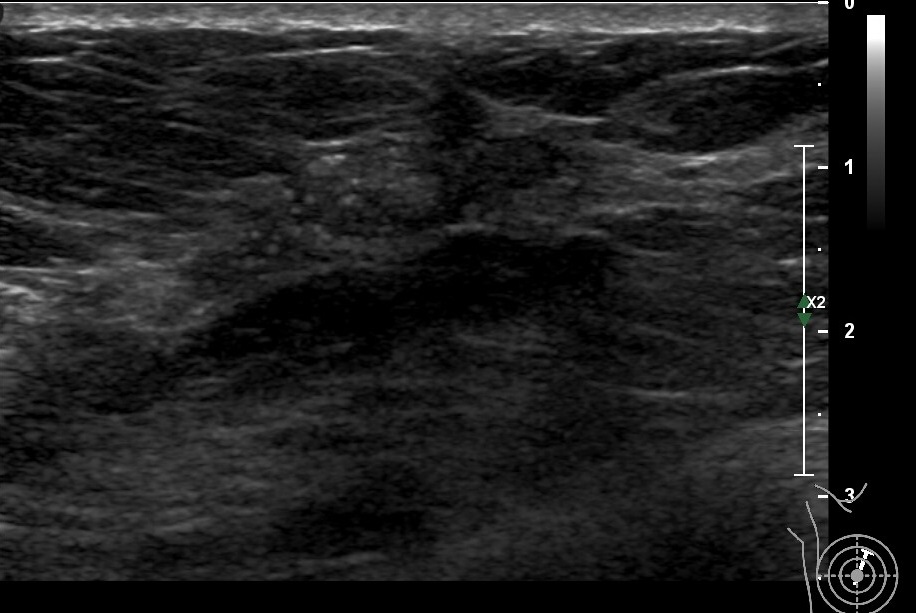

Supplement: S2 Data — Representative sonographic images of breast non-mass lesions. (ZIP) [file pone.0278299.s002.zip › Supplementary data 2/14/c.jpg]

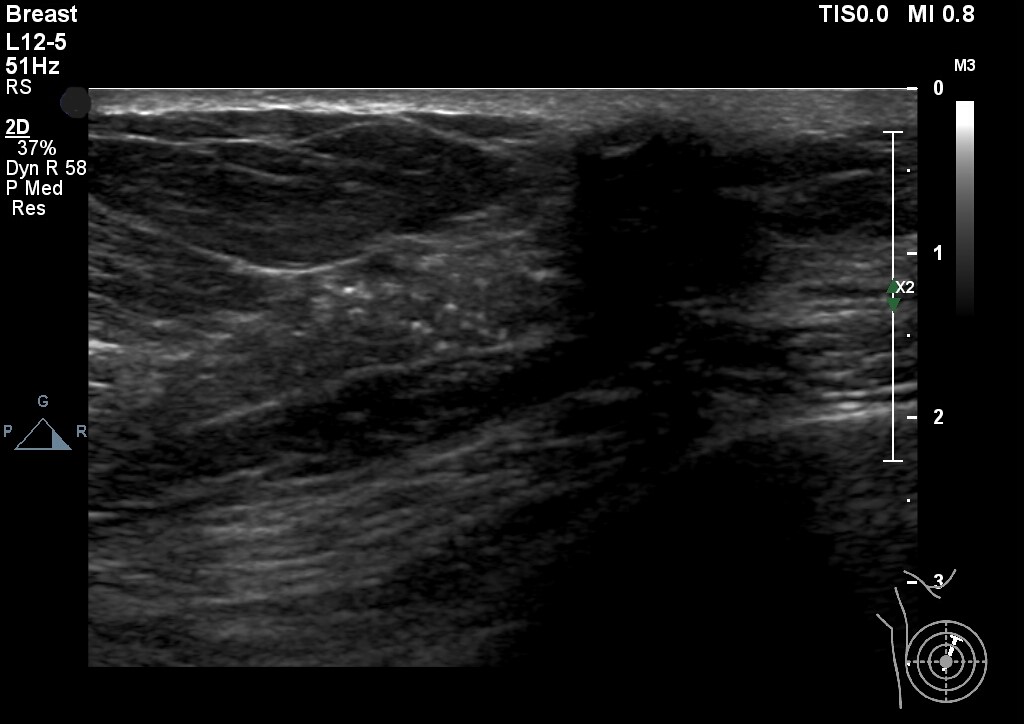

Supplement: S2 Data — Representative sonographic images of breast non-mass lesions. (ZIP) [file pone.0278299.s002.zip › Supplementary data 2/14/d.jpg]

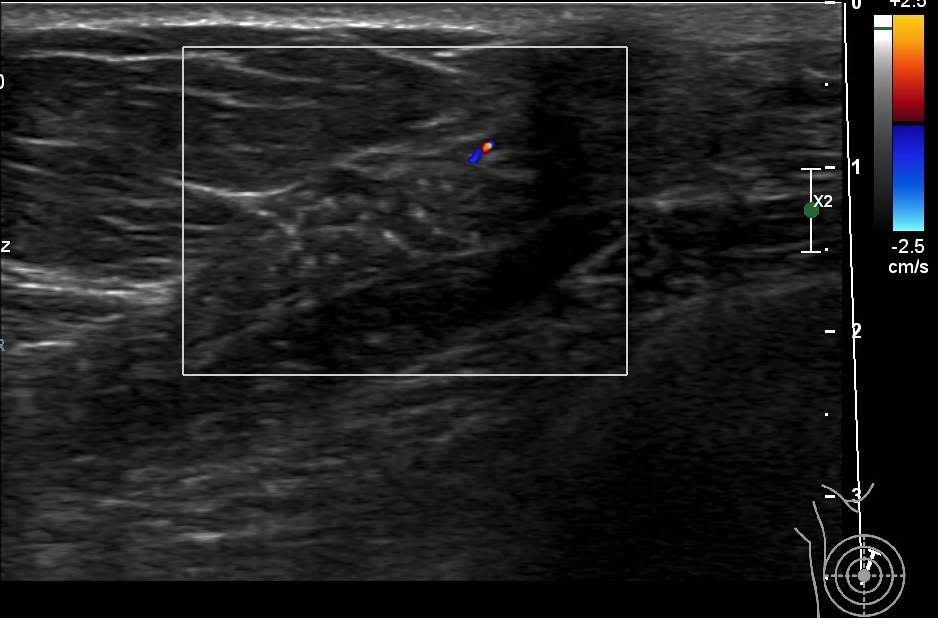

Supplement: S2 Data — Representative sonographic images of breast non-mass lesions. (ZIP) [file pone.0278299.s002.zip › Supplementary data 2/14/e.jpg]

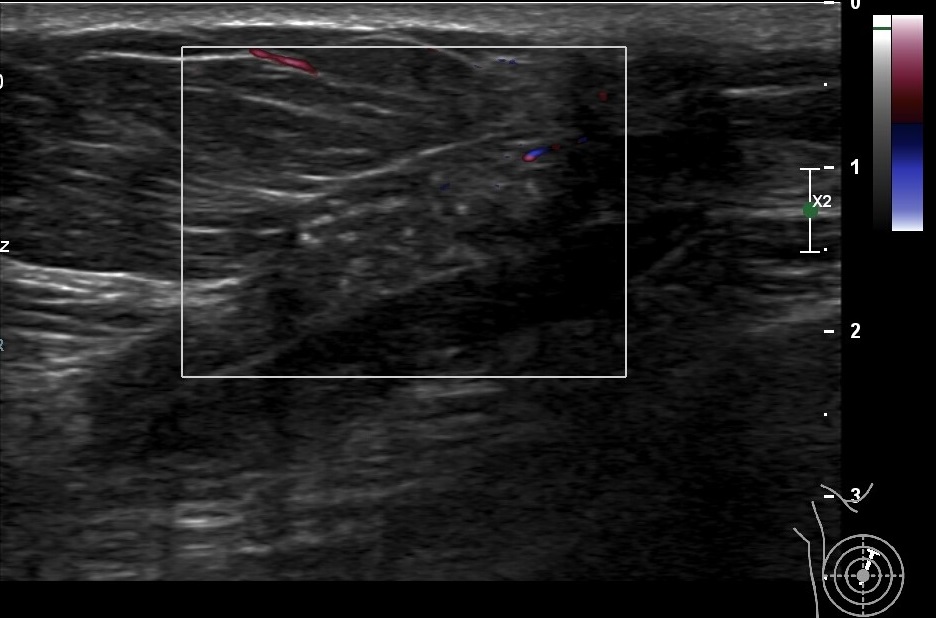

Supplement: S2 Data — Representative sonographic images of breast non-mass lesions. (ZIP) [file pone.0278299.s002.zip › Supplementary data 2/14/f.jpg]

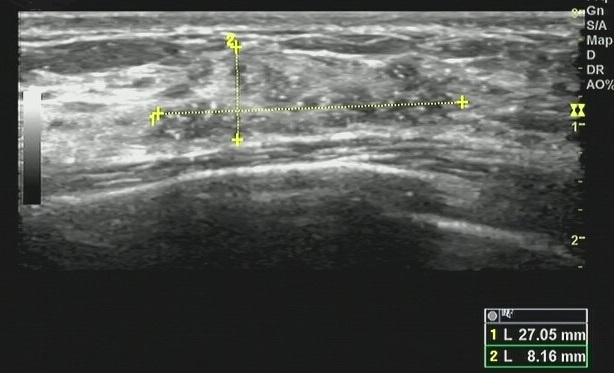

Supplement: S2 Data — Representative sonographic images of breast non-mass lesions. (ZIP) [file pone.0278299.s002.zip › Supplementary data 2/15/a.jpg]

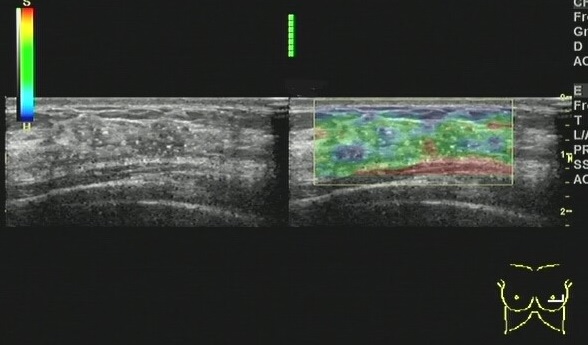

Supplement: S2 Data — Representative sonographic images of breast non-mass lesions. (ZIP) [file pone.0278299.s002.zip › Supplementary data 2/15/b.jpg]

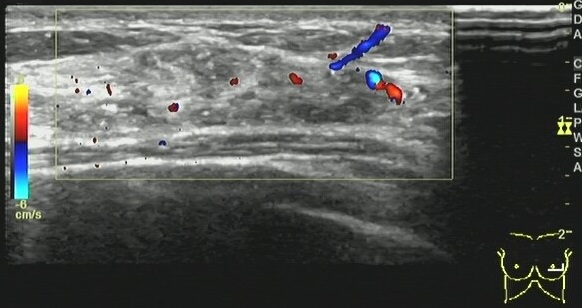

Supplement: S2 Data — Representative sonographic images of breast non-mass lesions. (ZIP) [file pone.0278299.s002.zip › Supplementary data 2/15/c.jpg]

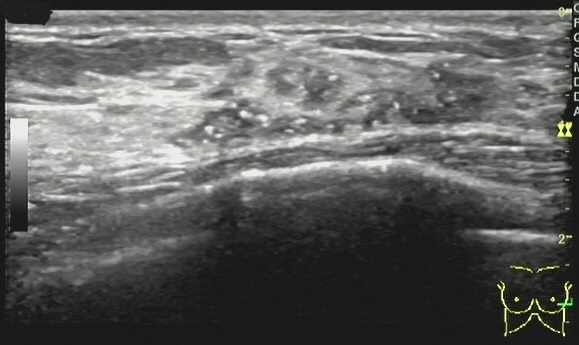

Supplement: S2 Data — Representative sonographic images of breast non-mass lesions. (ZIP) [file pone.0278299.s002.zip › Supplementary data 2/15/d.jpg]

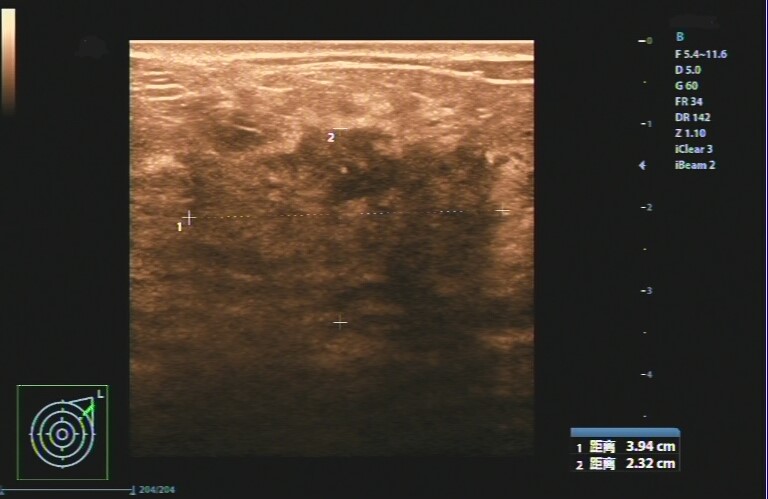

Supplement: S2 Data — Representative sonographic images of breast non-mass lesions. (ZIP) [file pone.0278299.s002.zip › Supplementary data 2/16/a.jpg]

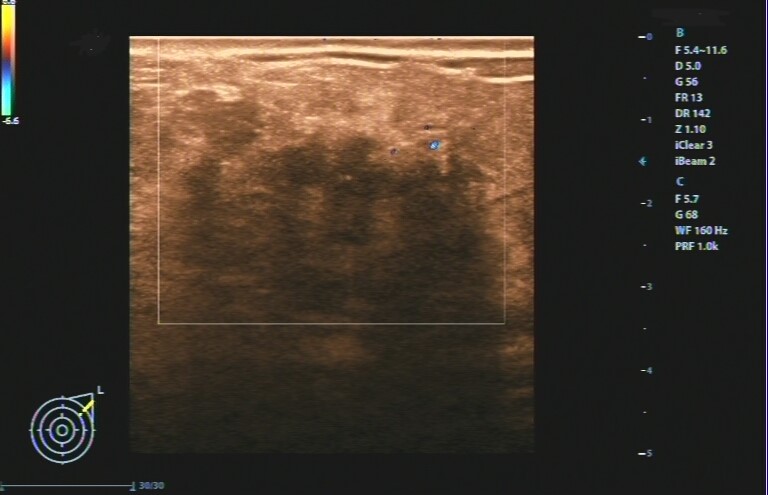

Supplement: S2 Data — Representative sonographic images of breast non-mass lesions. (ZIP) [file pone.0278299.s002.zip › Supplementary data 2/16/b.jpg]

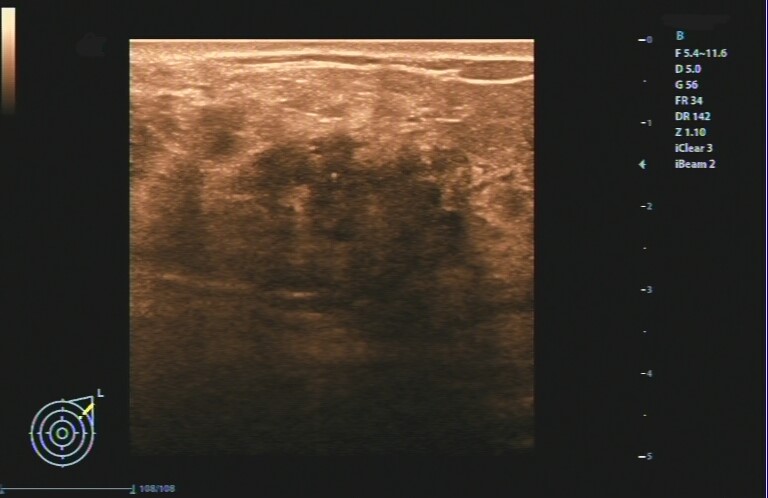

Supplement: S2 Data — Representative sonographic images of breast non-mass lesions. (ZIP) [file pone.0278299.s002.zip › Supplementary data 2/16/c.jpg]

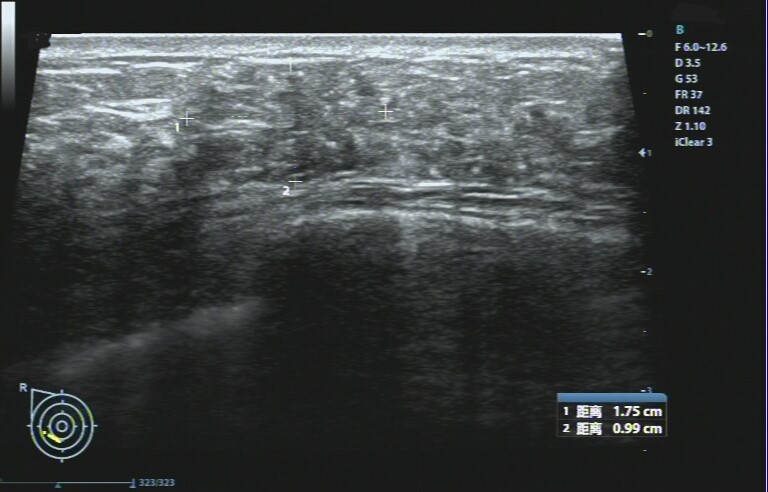

Supplement: S2 Data — Representative sonographic images of breast non-mass lesions. (ZIP) [file pone.0278299.s002.zip › Supplementary data 2/17/a.jpg]

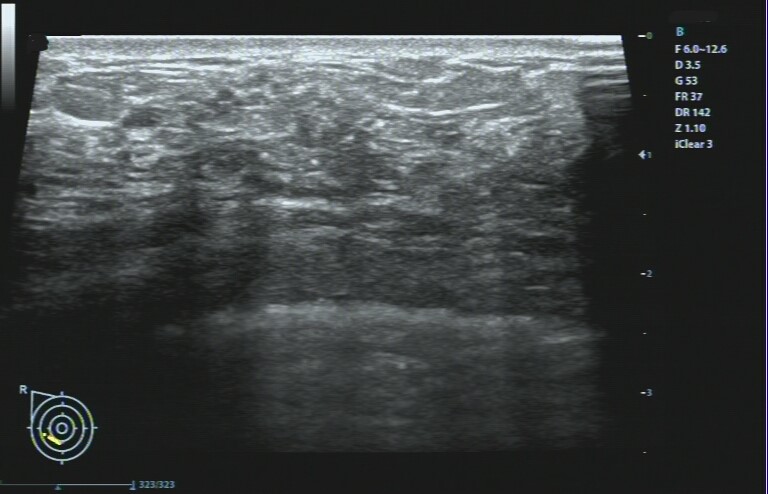

Supplement: S2 Data — Representative sonographic images of breast non-mass lesions. (ZIP) [file pone.0278299.s002.zip › Supplementary data 2/17/b.jpg]

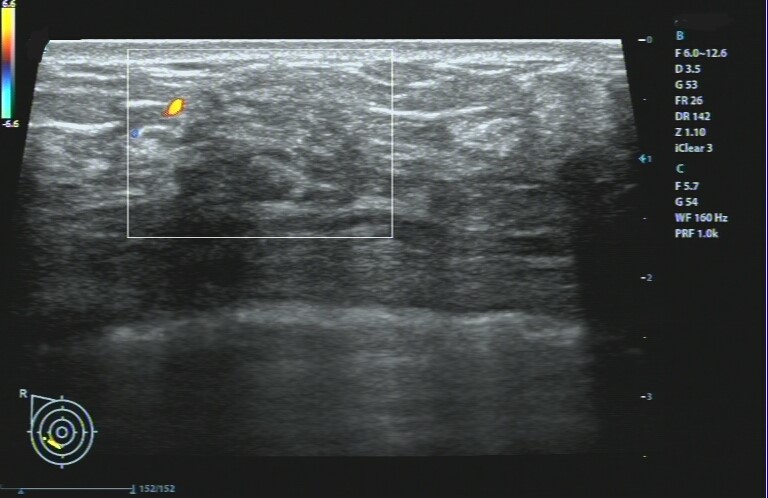

Supplement: S2 Data — Representative sonographic images of breast non-mass lesions. (ZIP) [file pone.0278299.s002.zip › Supplementary data 2/17/c.jpg]

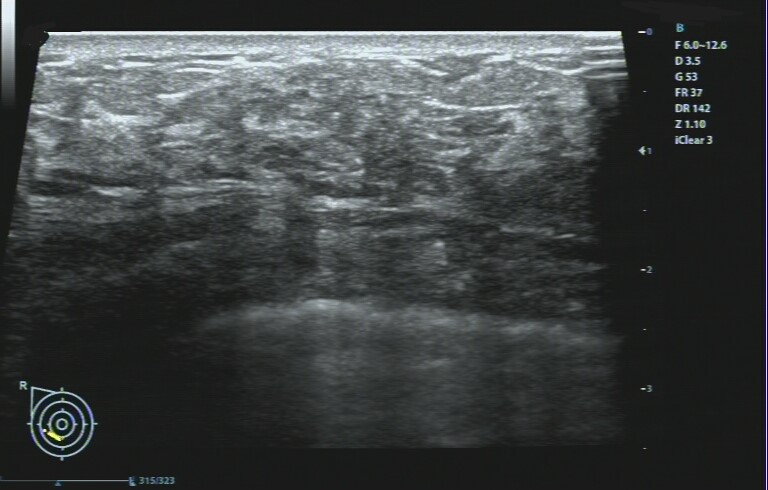

Supplement: S2 Data — Representative sonographic images of breast non-mass lesions. (ZIP) [file pone.0278299.s002.zip › Supplementary data 2/17/d.jpg]

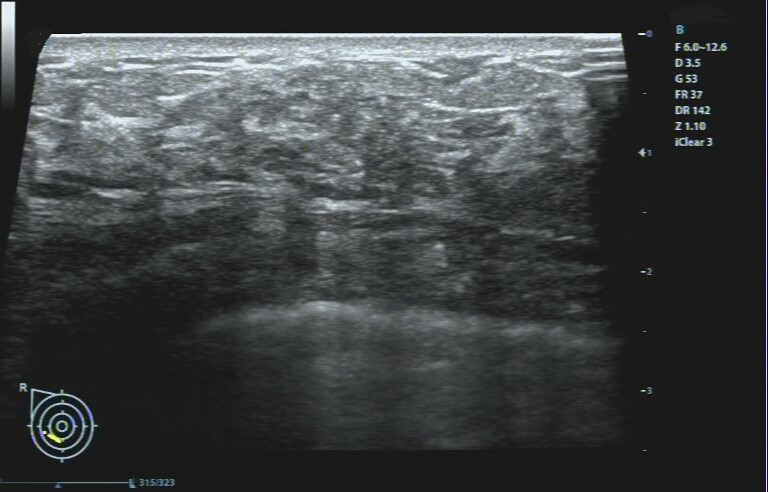

Supplement: S2 Data — Representative sonographic images of breast non-mass lesions. (ZIP) [file pone.0278299.s002.zip › Supplementary data 2/17/e.jpg]

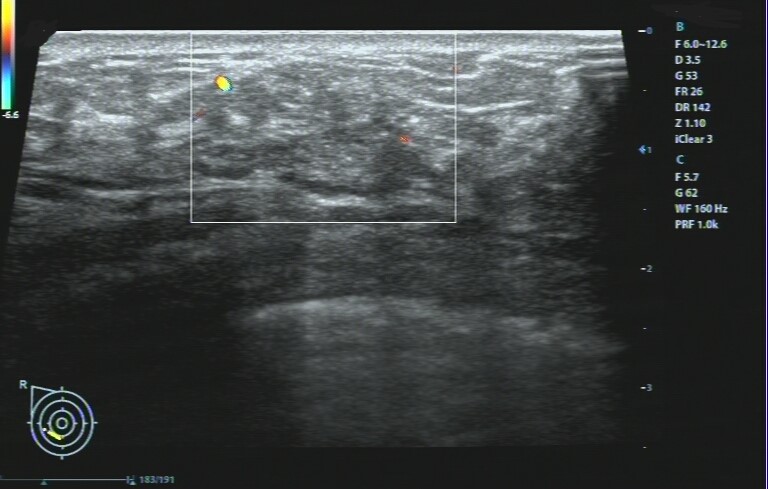

Supplement: S2 Data — Representative sonographic images of breast non-mass lesions. (ZIP) [file pone.0278299.s002.zip › Supplementary data 2/17/f.jpg]

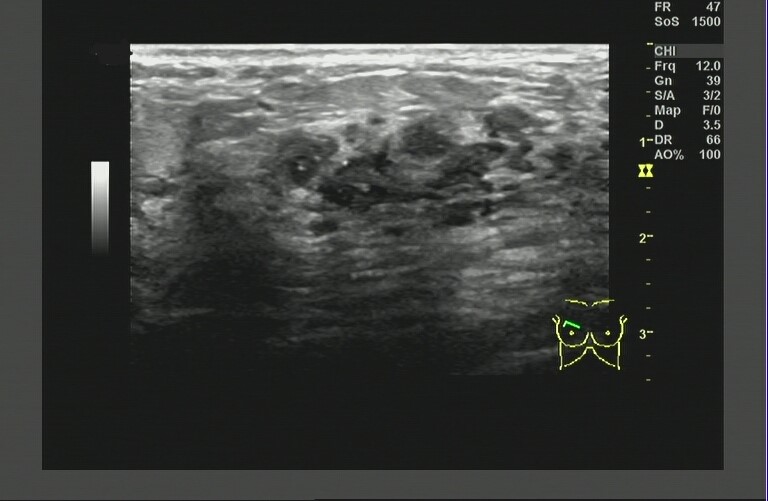

Supplement: S2 Data — Representative sonographic images of breast non-mass lesions. (ZIP) [file pone.0278299.s002.zip › Supplementary data 2/18/a.jpg]

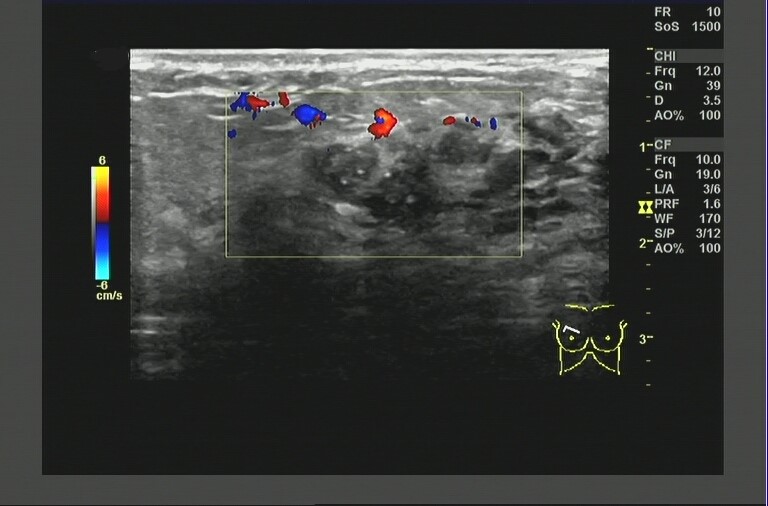

Supplement: S2 Data — Representative sonographic images of breast non-mass lesions. (ZIP) [file pone.0278299.s002.zip › Supplementary data 2/18/b.jpg]

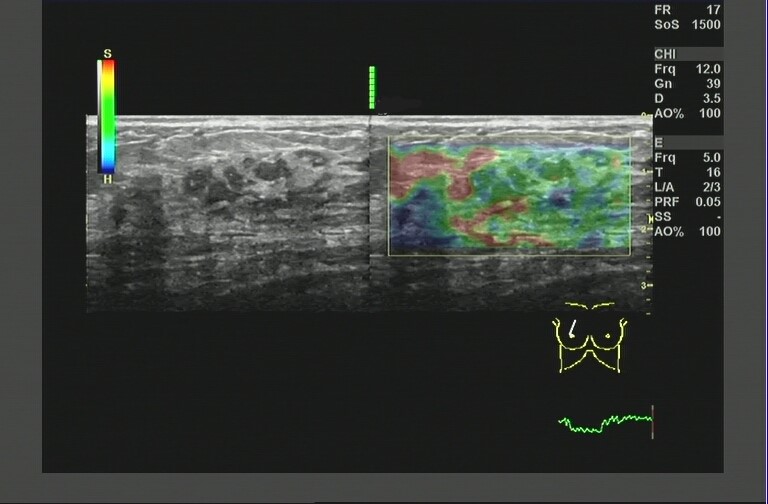

Supplement: S2 Data — Representative sonographic images of breast non-mass lesions. (ZIP) [file pone.0278299.s002.zip › Supplementary data 2/18/c.jpg]

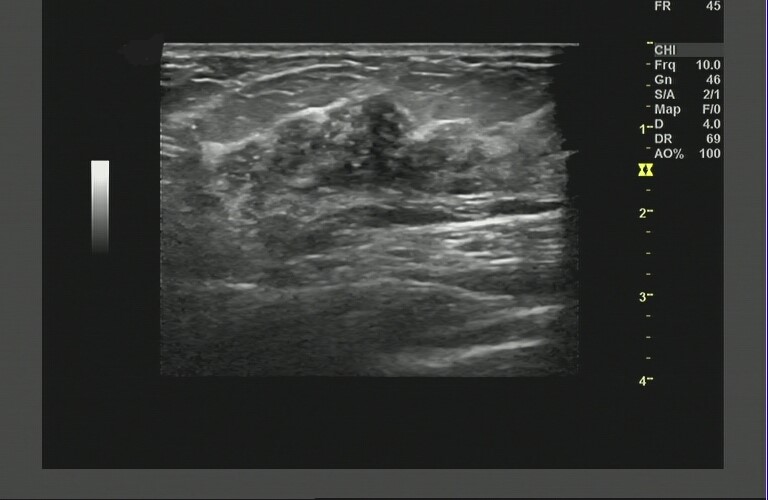

Supplement: S2 Data — Representative sonographic images of breast non-mass lesions. (ZIP) [file pone.0278299.s002.zip › Supplementary data 2/19/a.jpg]

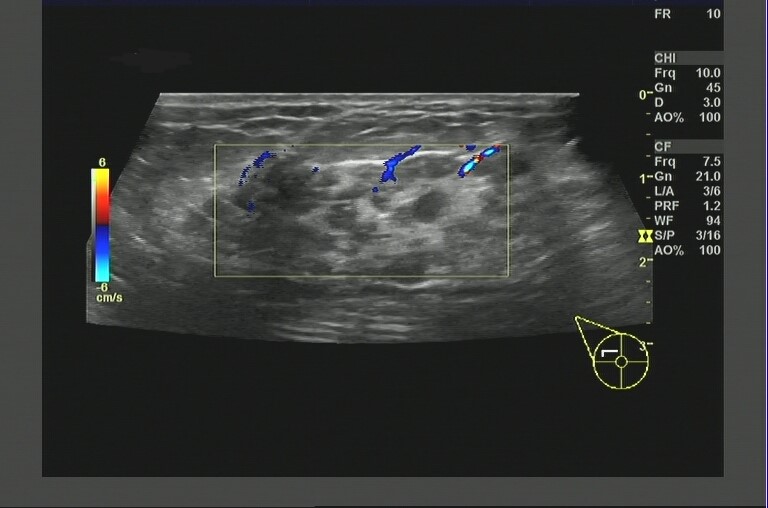

Supplement: S2 Data — Representative sonographic images of breast non-mass lesions. (ZIP) [file pone.0278299.s002.zip › Supplementary data 2/19/b.jpg]

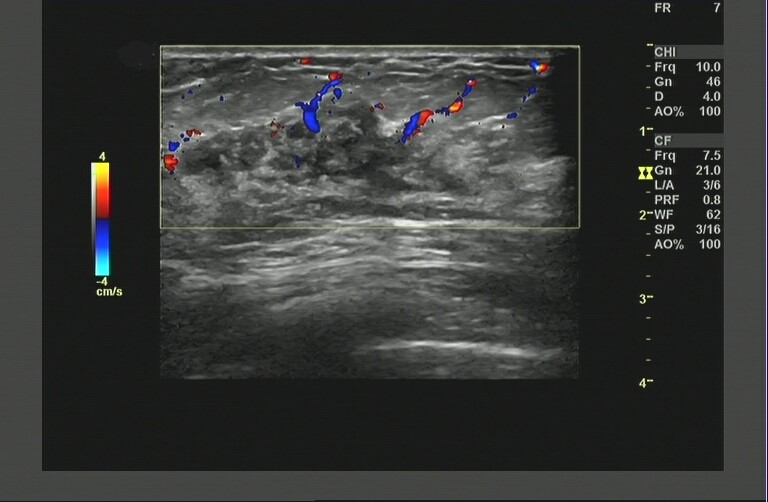

Supplement: S2 Data — Representative sonographic images of breast non-mass lesions. (ZIP) [file pone.0278299.s002.zip › Supplementary data 2/19/e.jpg]

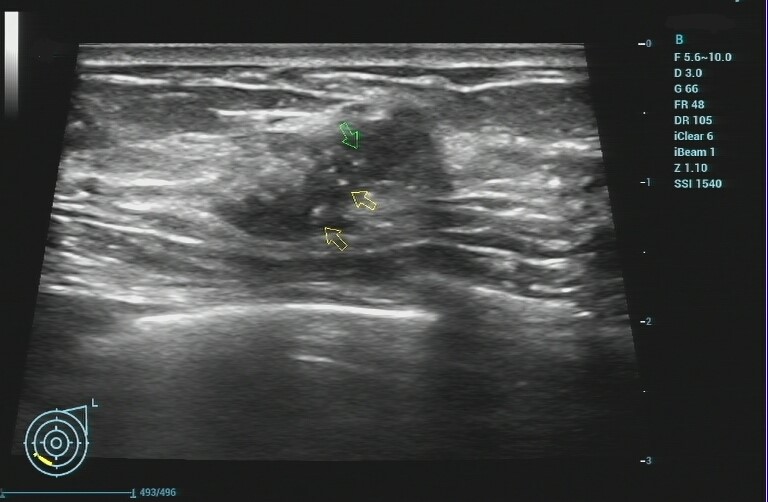

Supplement: S2 Data — Representative sonographic images of breast non-mass lesions. (ZIP) [file pone.0278299.s002.zip › Supplementary data 2/2/a.jpg]

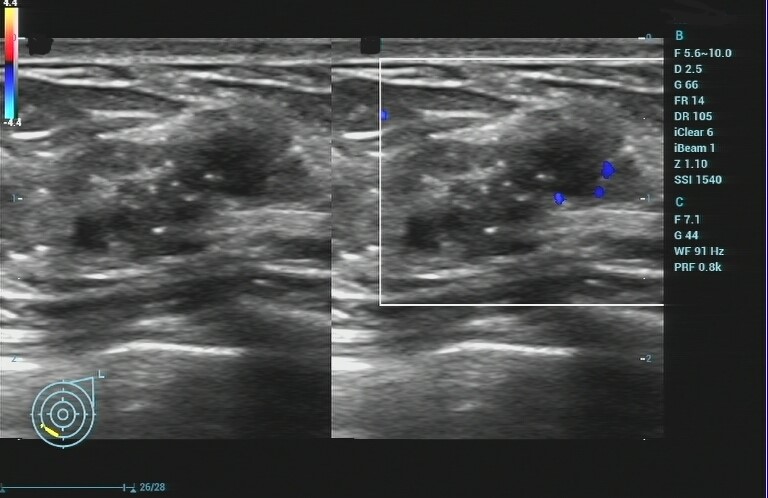

Supplement: S2 Data — Representative sonographic images of breast non-mass lesions. (ZIP) [file pone.0278299.s002.zip › Supplementary data 2/2/b.jpg]

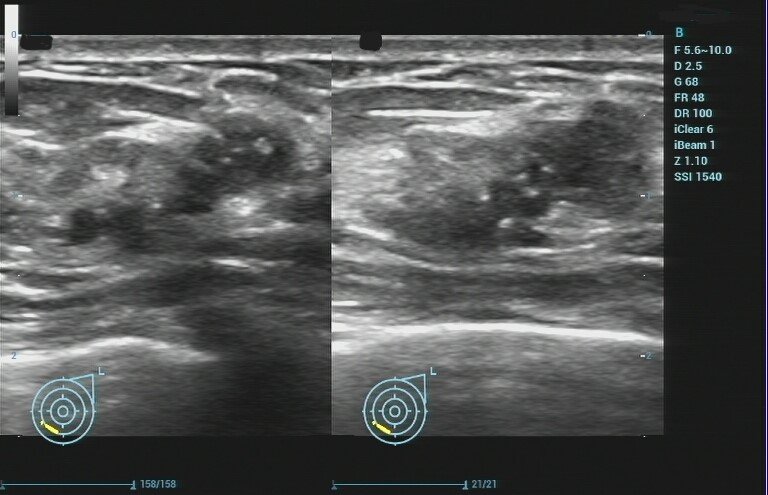

Supplement: S2 Data — Representative sonographic images of breast non-mass lesions. (ZIP) [file pone.0278299.s002.zip › Supplementary data 2/2/c.jpg]

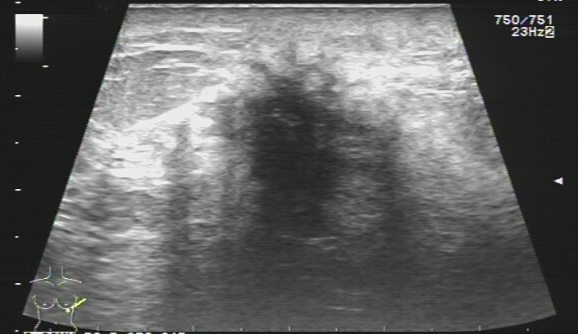

Supplement: S2 Data — Representative sonographic images of breast non-mass lesions. (ZIP) [file pone.0278299.s002.zip › Supplementary data 2/20/a.jpg]

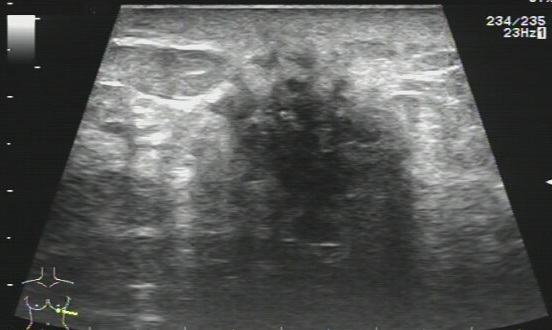

Supplement: S2 Data — Representative sonographic images of breast non-mass lesions. (ZIP) [file pone.0278299.s002.zip › Supplementary data 2/20/b.jpg]

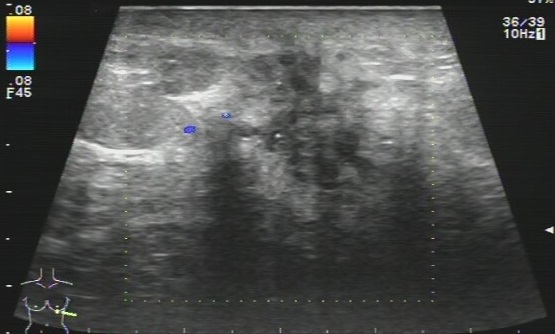

Supplement: S2 Data — Representative sonographic images of breast non-mass lesions. (ZIP) [file pone.0278299.s002.zip › Supplementary data 2/20/c.jpg]

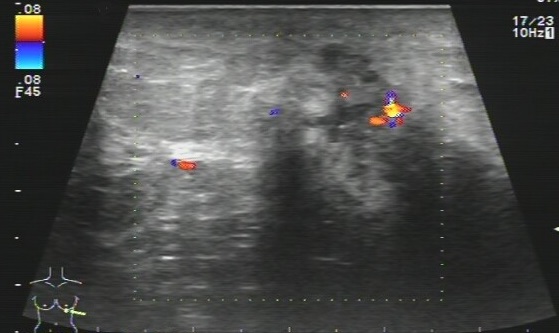

Supplement: S2 Data — Representative sonographic images of breast non-mass lesions. (ZIP) [file pone.0278299.s002.zip › Supplementary data 2/20/d.jpg]

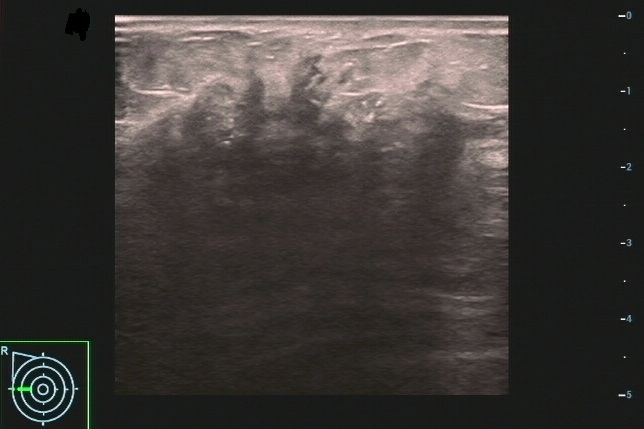

Supplement: S2 Data — Representative sonographic images of breast non-mass lesions. (ZIP) [file pone.0278299.s002.zip › Supplementary data 2/21/a.jpg]

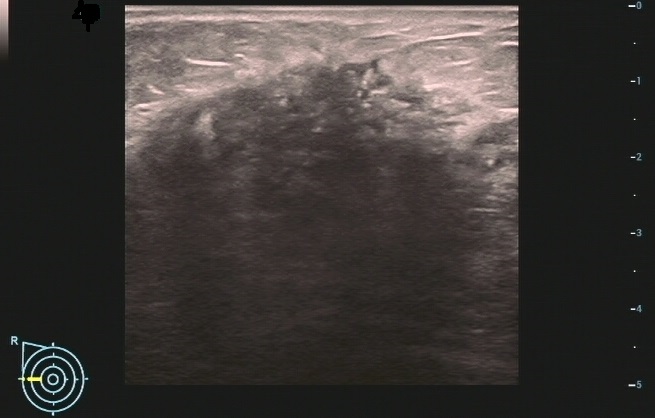

Supplement: S2 Data — Representative sonographic images of breast non-mass lesions. (ZIP) [file pone.0278299.s002.zip › Supplementary data 2/21/b.jpg]

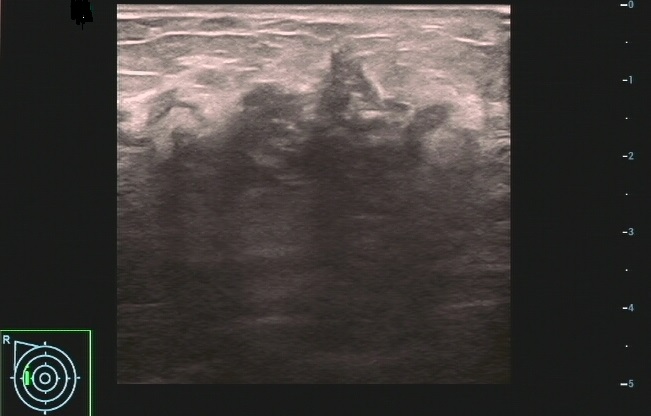

Supplement: S2 Data — Representative sonographic images of breast non-mass lesions. (ZIP) [file pone.0278299.s002.zip › Supplementary data 2/21/c.jpg]

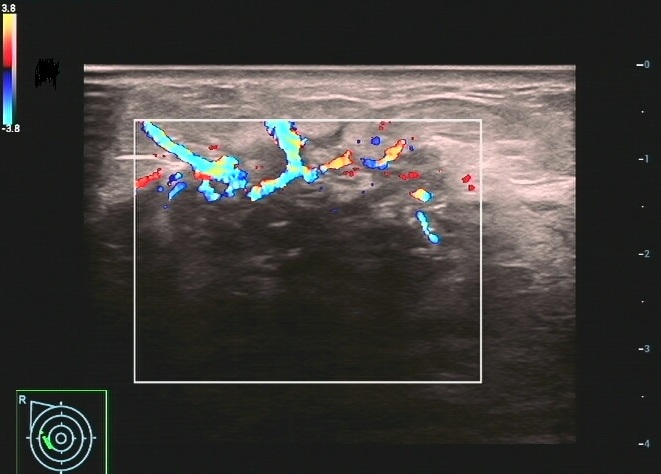

Supplement: S2 Data — Representative sonographic images of breast non-mass lesions. (ZIP) [file pone.0278299.s002.zip › Supplementary data 2/21/d.jpg]

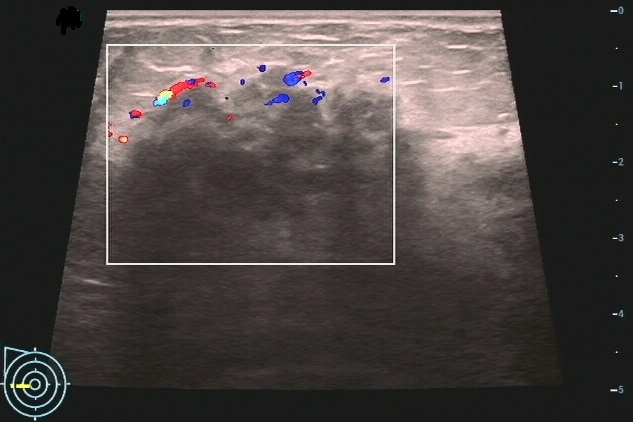

Supplement: S2 Data — Representative sonographic images of breast non-mass lesions. (ZIP) [file pone.0278299.s002.zip › Supplementary data 2/21/e.jpg]

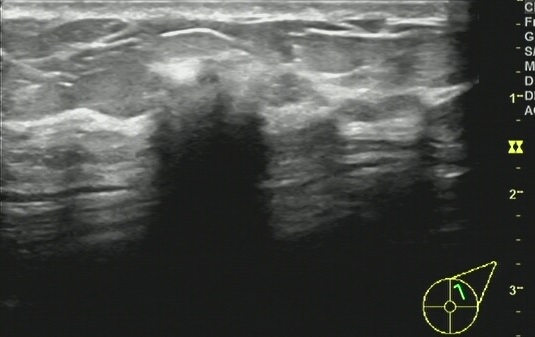

Supplement: S2 Data — Representative sonographic images of breast non-mass lesions. (ZIP) [file pone.0278299.s002.zip › Supplementary data 2/22/a.jpg]

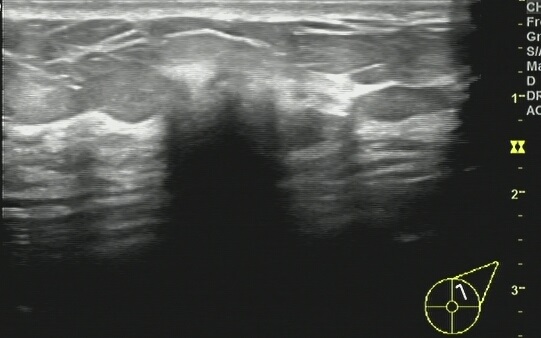

Supplement: S2 Data — Representative sonographic images of breast non-mass lesions. (ZIP) [file pone.0278299.s002.zip › Supplementary data 2/22/b.jpg]

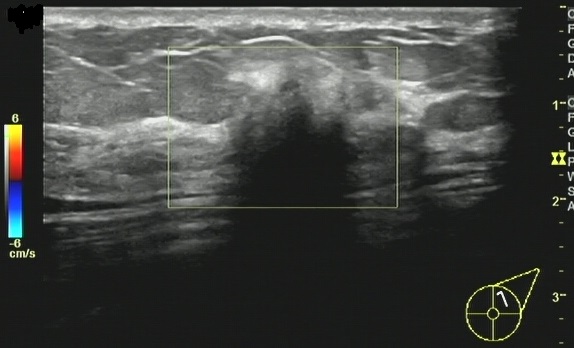

Supplement: S2 Data — Representative sonographic images of breast non-mass lesions. (ZIP) [file pone.0278299.s002.zip › Supplementary data 2/22/c.jpg]

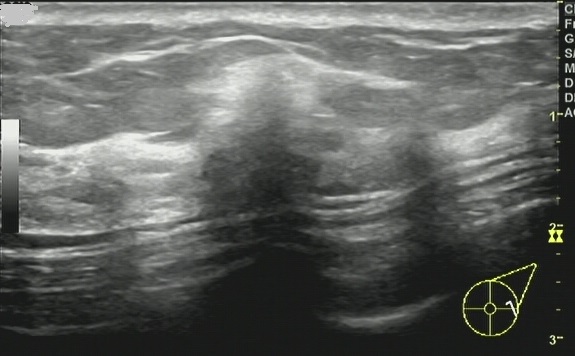

Supplement: S2 Data — Representative sonographic images of breast non-mass lesions. (ZIP) [file pone.0278299.s002.zip › Supplementary data 2/22/d.jpg]

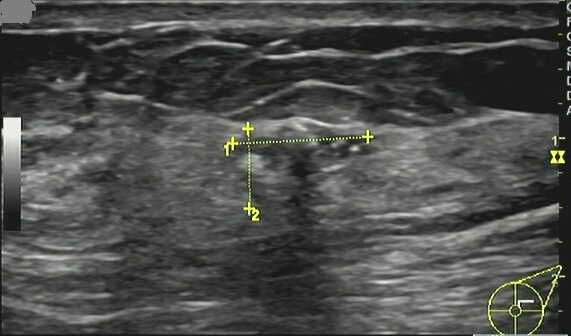

Supplement: S2 Data — Representative sonographic images of breast non-mass lesions. (ZIP) [file pone.0278299.s002.zip › Supplementary data 2/23/a.jpg]

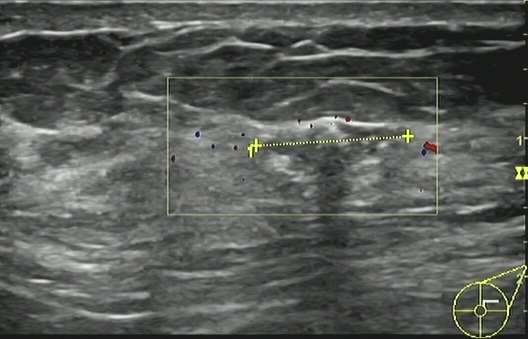

Supplement: S2 Data — Representative sonographic images of breast non-mass lesions. (ZIP) [file pone.0278299.s002.zip › Supplementary data 2/23/b.jpg]

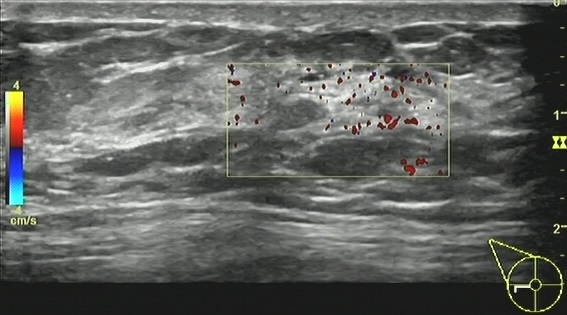

Supplement: S2 Data — Representative sonographic images of breast non-mass lesions. (ZIP) [file pone.0278299.s002.zip › Supplementary data 2/23/c.jpg]

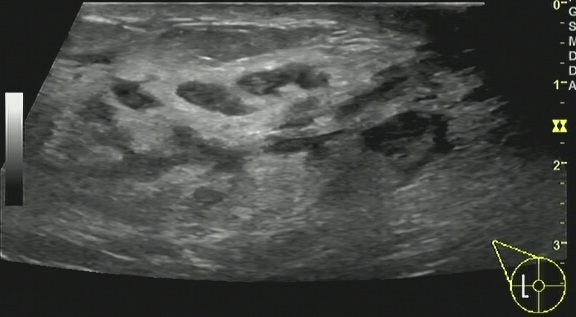

Supplement: S2 Data — Representative sonographic images of breast non-mass lesions. (ZIP) [file pone.0278299.s002.zip › Supplementary data 2/24/a.jpg]

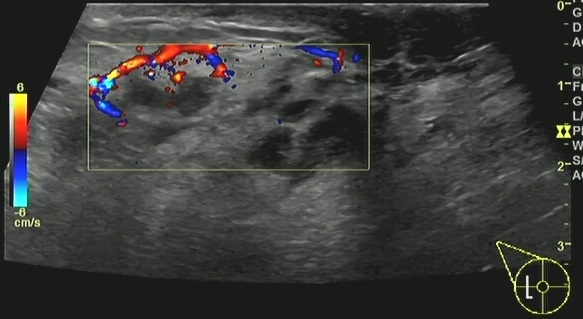

Supplement: S2 Data — Representative sonographic images of breast non-mass lesions. (ZIP) [file pone.0278299.s002.zip › Supplementary data 2/24/b.jpg]

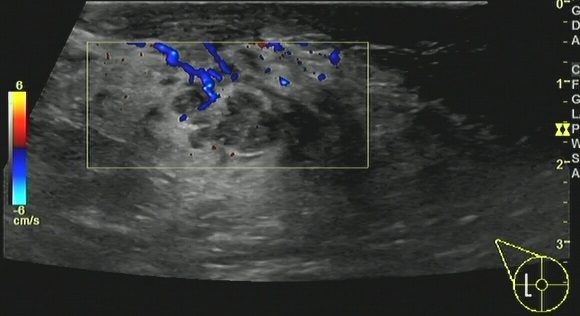

Supplement: S2 Data — Representative sonographic images of breast non-mass lesions. (ZIP) [file pone.0278299.s002.zip › Supplementary data 2/24/c.jpg]

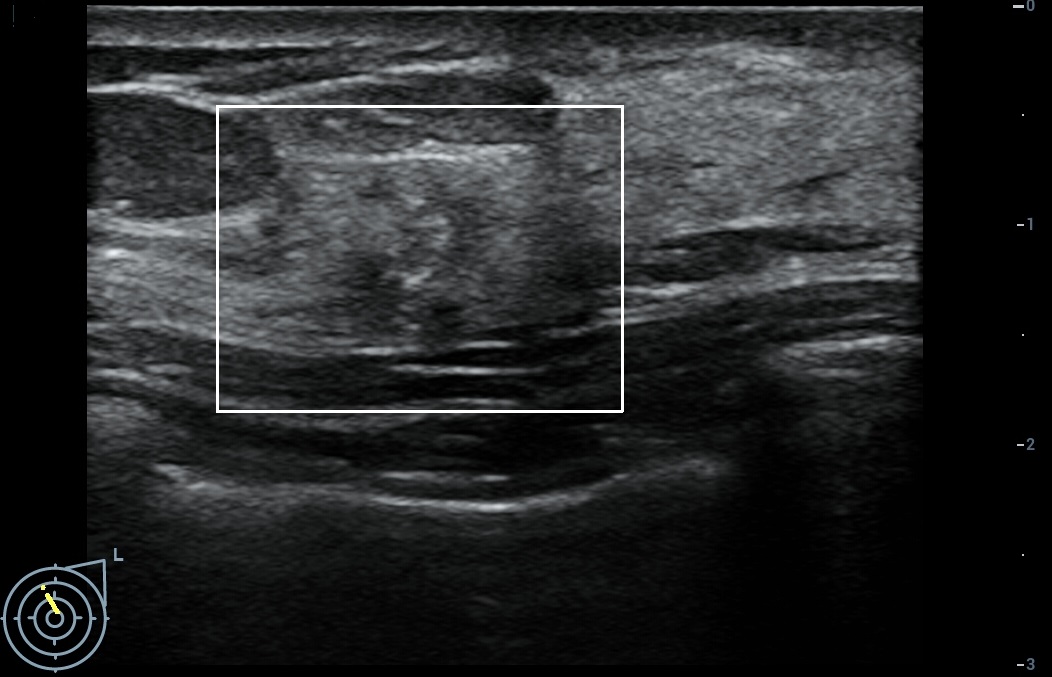

Supplement: S2 Data — Representative sonographic images of breast non-mass lesions. (ZIP) [file pone.0278299.s002.zip › Supplementary data 2/25/a.jpg]

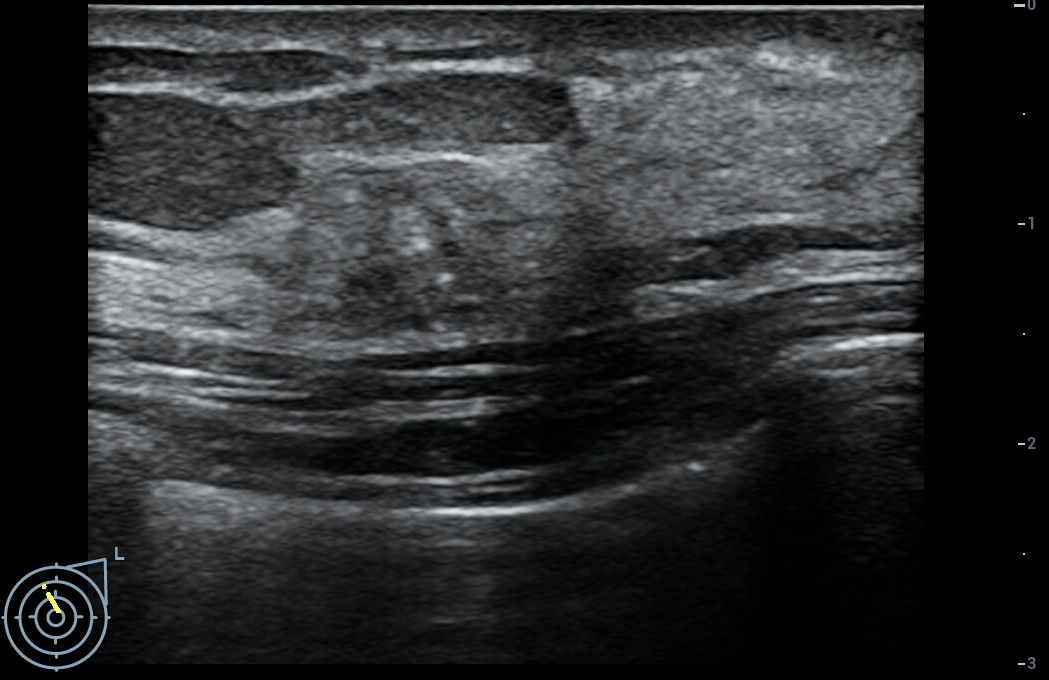

Supplement: S2 Data — Representative sonographic images of breast non-mass lesions. (ZIP) [file pone.0278299.s002.zip › Supplementary data 2/25/b.jpg]

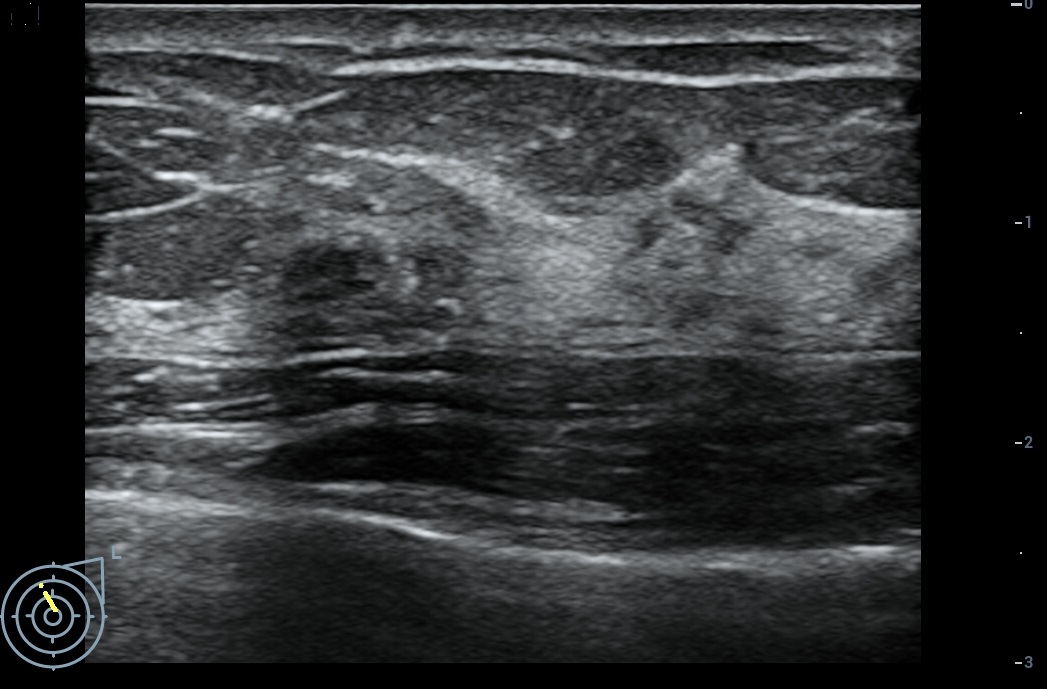

Supplement: S2 Data — Representative sonographic images of breast non-mass lesions. (ZIP) [file pone.0278299.s002.zip › Supplementary data 2/25/c.jpg]

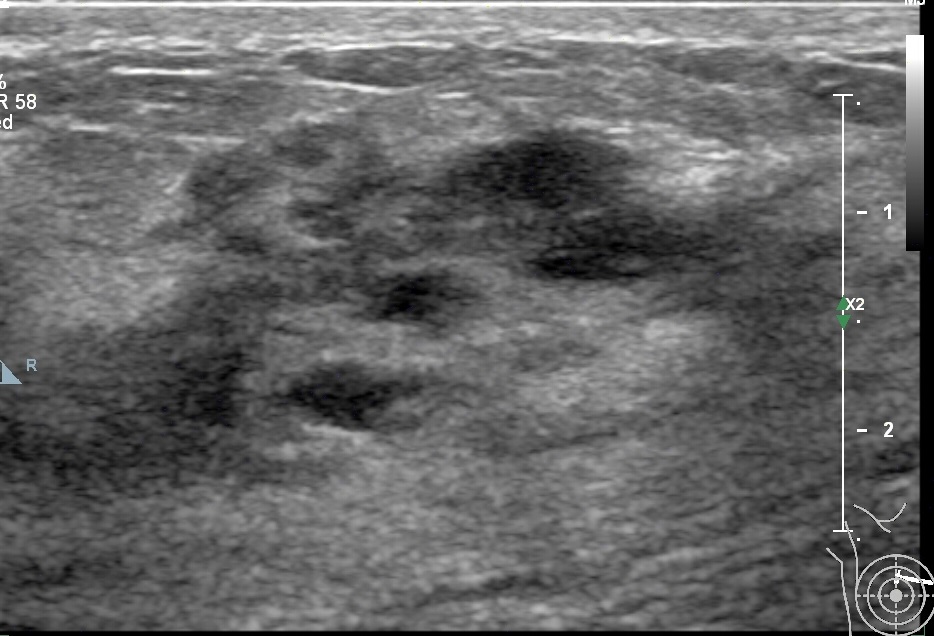

Supplement: S2 Data — Representative sonographic images of breast non-mass lesions. (ZIP) [file pone.0278299.s002.zip › Supplementary data 2/26/a.jpg]

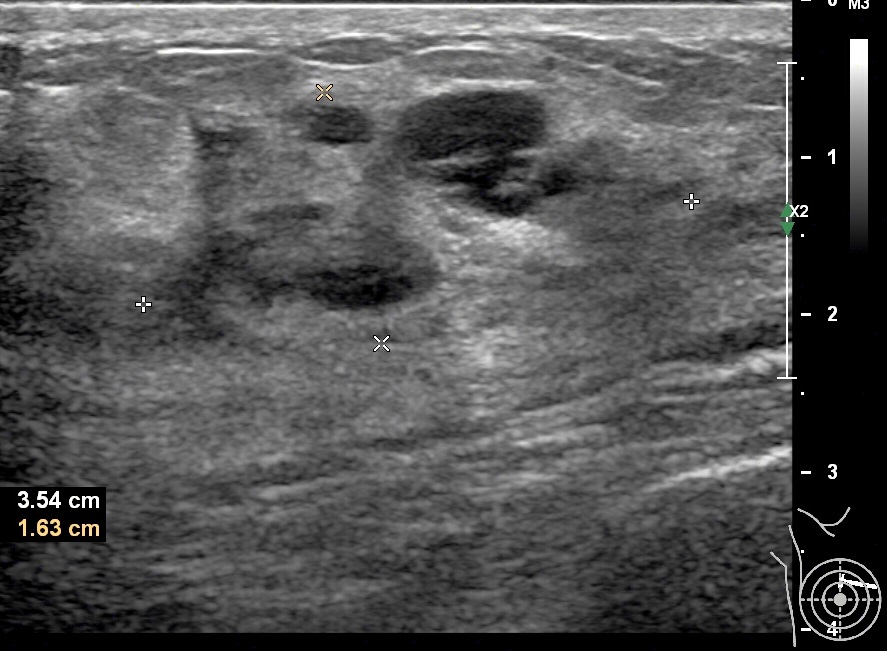

Supplement: S2 Data — Representative sonographic images of breast non-mass lesions. (ZIP) [file pone.0278299.s002.zip › Supplementary data 2/26/b.jpg]

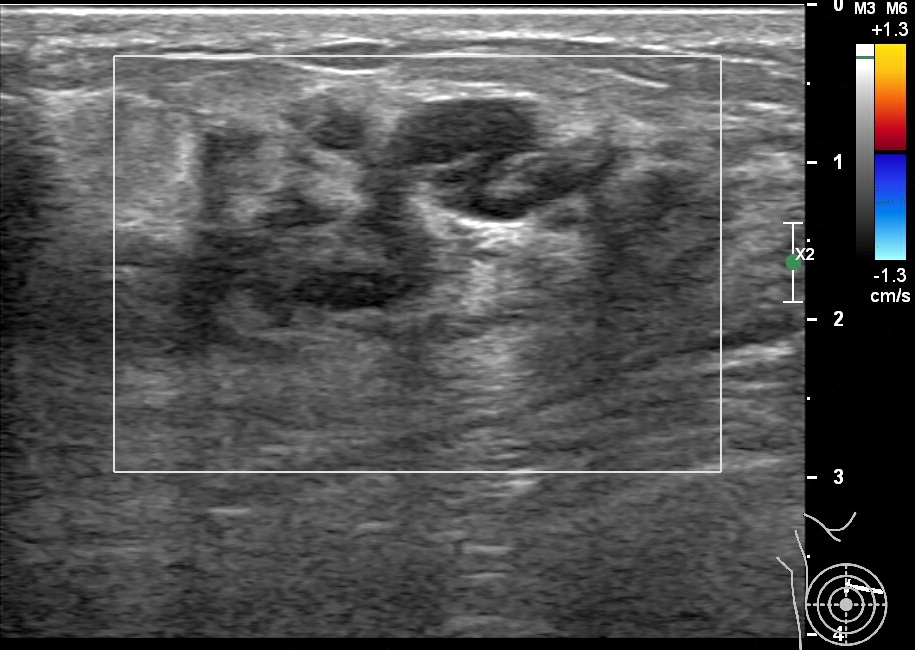

Supplement: S2 Data — Representative sonographic images of breast non-mass lesions. (ZIP) [file pone.0278299.s002.zip › Supplementary data 2/26/c.jpg]

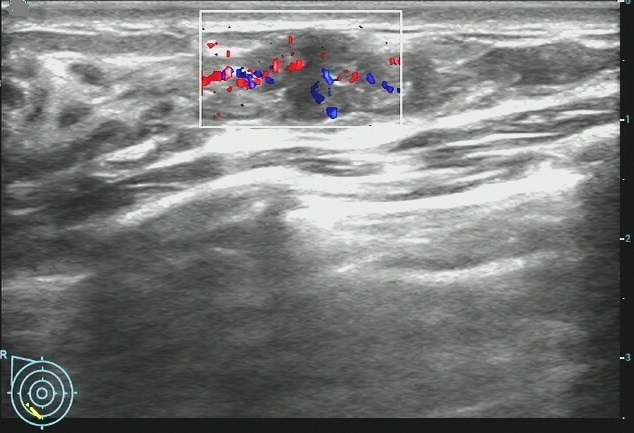

Supplement: S2 Data — Representative sonographic images of breast non-mass lesions. (ZIP) [file pone.0278299.s002.zip › Supplementary data 2/27/a.jpg]

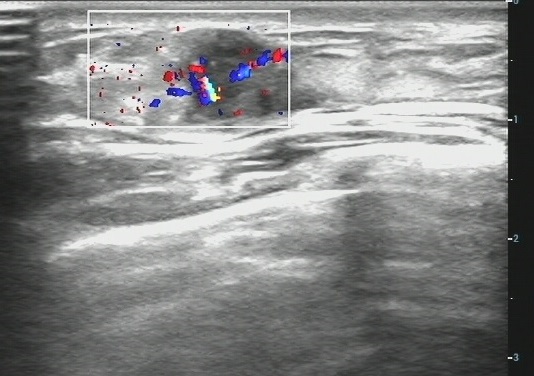

Supplement: S2 Data — Representative sonographic images of breast non-mass lesions. (ZIP) [file pone.0278299.s002.zip › Supplementary data 2/27/b.jpg]

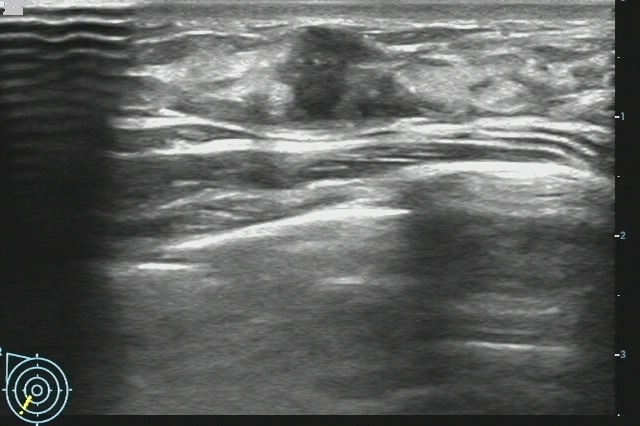

Supplement: S2 Data — Representative sonographic images of breast non-mass lesions. (ZIP) [file pone.0278299.s002.zip › Supplementary data 2/27/c.jpg]

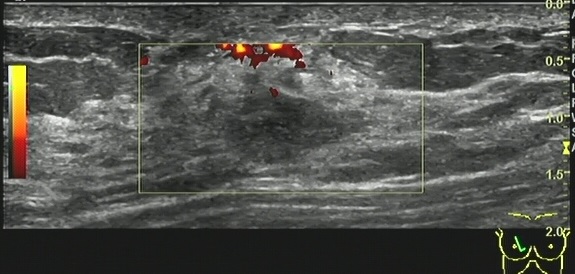

Supplement: S2 Data — Representative sonographic images of breast non-mass lesions. (ZIP) [file pone.0278299.s002.zip › Supplementary data 2/28/a.jpg]

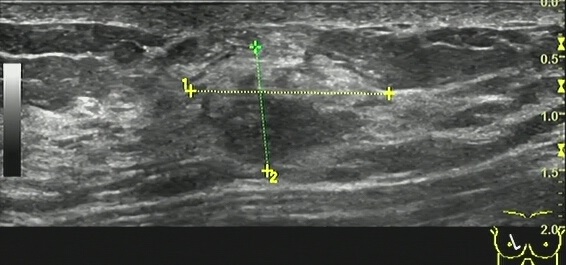

Supplement: S2 Data — Representative sonographic images of breast non-mass lesions. (ZIP) [file pone.0278299.s002.zip › Supplementary data 2/28/b.jpg]

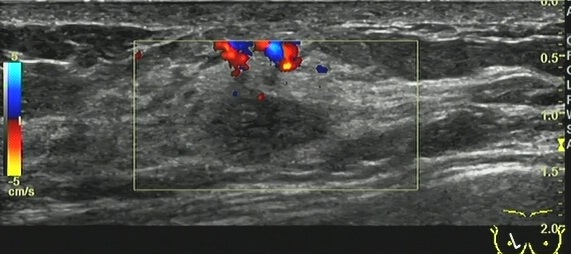

Supplement: S2 Data — Representative sonographic images of breast non-mass lesions. (ZIP) [file pone.0278299.s002.zip › Supplementary data 2/28/c.jpg]

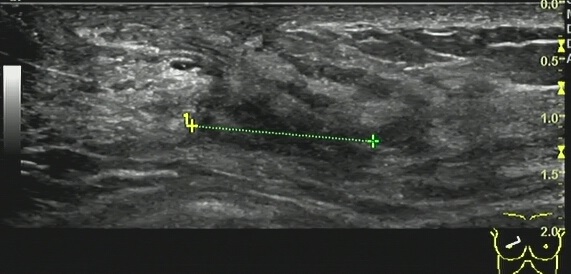

Supplement: S2 Data — Representative sonographic images of breast non-mass lesions. (ZIP) [file pone.0278299.s002.zip › Supplementary data 2/28/d.jpg]

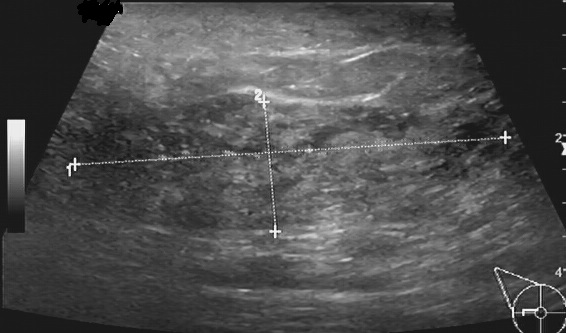

Supplement: S2 Data — Representative sonographic images of breast non-mass lesions. (ZIP) [file pone.0278299.s002.zip › Supplementary data 2/29/a.jpg]

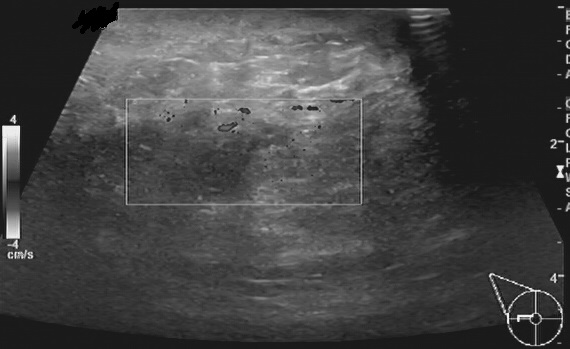

Supplement: S2 Data — Representative sonographic images of breast non-mass lesions. (ZIP) [file pone.0278299.s002.zip › Supplementary data 2/29/b.jpg]

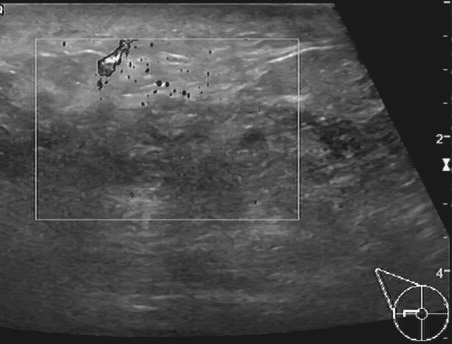

Supplement: S2 Data — Representative sonographic images of breast non-mass lesions. (ZIP) [file pone.0278299.s002.zip › Supplementary data 2/29/c.jpg]

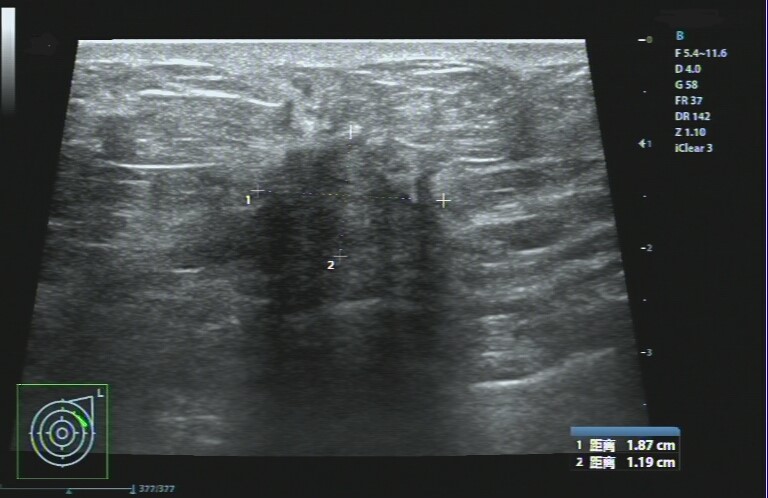

Supplement: S2 Data — Representative sonographic images of breast non-mass lesions. (ZIP) [file pone.0278299.s002.zip › Supplementary data 2/3/a.jpg]

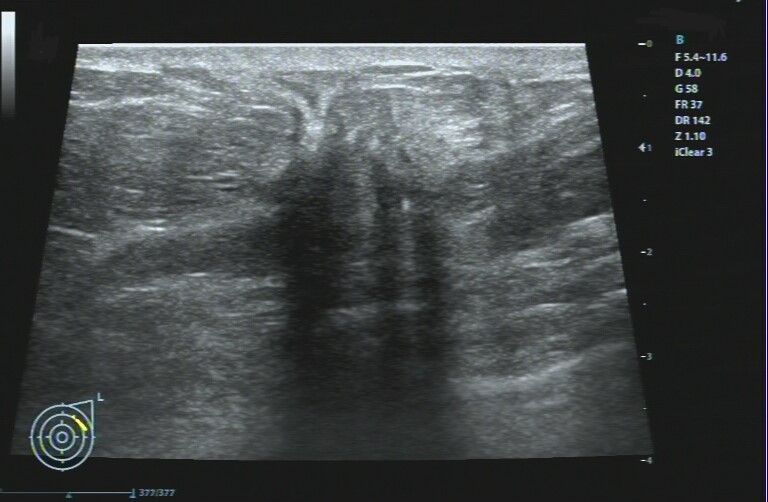

Supplement: S2 Data — Representative sonographic images of breast non-mass lesions. (ZIP) [file pone.0278299.s002.zip › Supplementary data 2/3/b.jpg]

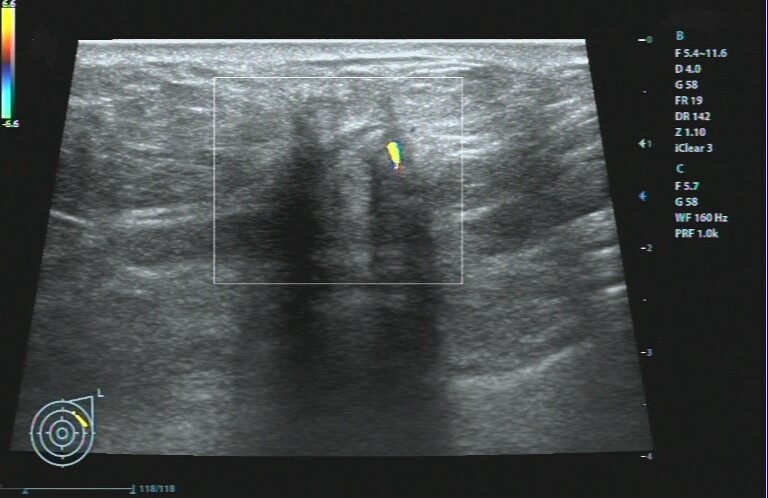

Supplement: S2 Data — Representative sonographic images of breast non-mass lesions. (ZIP) [file pone.0278299.s002.zip › Supplementary data 2/3/c.jpg]

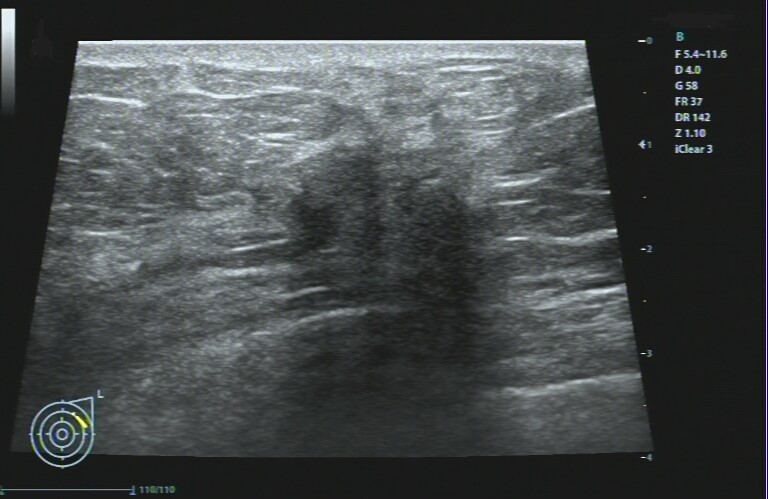

Supplement: S2 Data — Representative sonographic images of breast non-mass lesions. (ZIP) [file pone.0278299.s002.zip › Supplementary data 2/3/d.jpg]

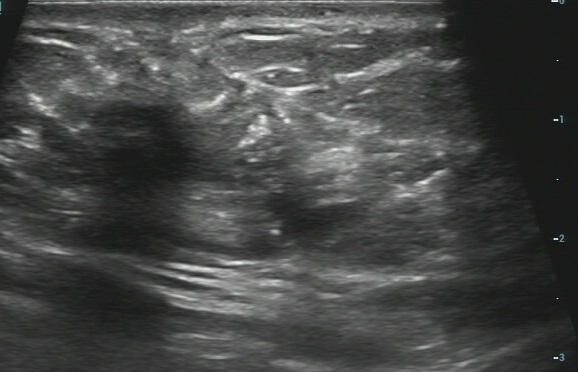

Supplement: S2 Data — Representative sonographic images of breast non-mass lesions. (ZIP) [file pone.0278299.s002.zip › Supplementary data 2/30/a.jpg]

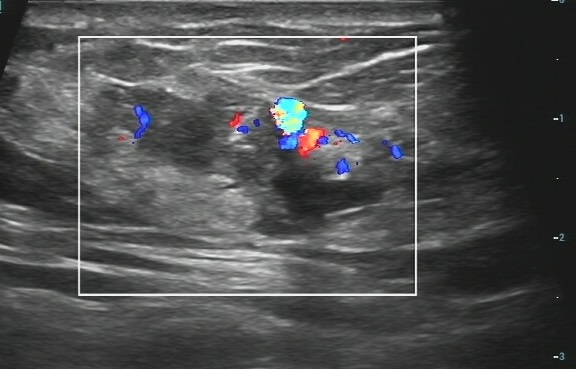

Supplement: S2 Data — Representative sonographic images of breast non-mass lesions. (ZIP) [file pone.0278299.s002.zip › Supplementary data 2/30/b.jpg]

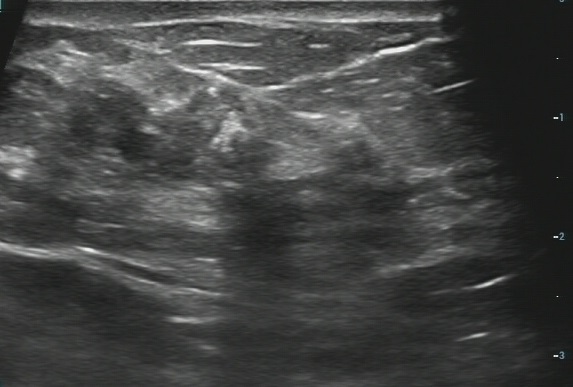

Supplement: S2 Data — Representative sonographic images of breast non-mass lesions. (ZIP) [file pone.0278299.s002.zip › Supplementary data 2/30/c.jpg]

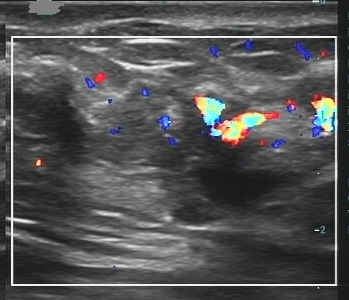

Supplement: S2 Data — Representative sonographic images of breast non-mass lesions. (ZIP) [file pone.0278299.s002.zip › Supplementary data 2/30/d.jpg]

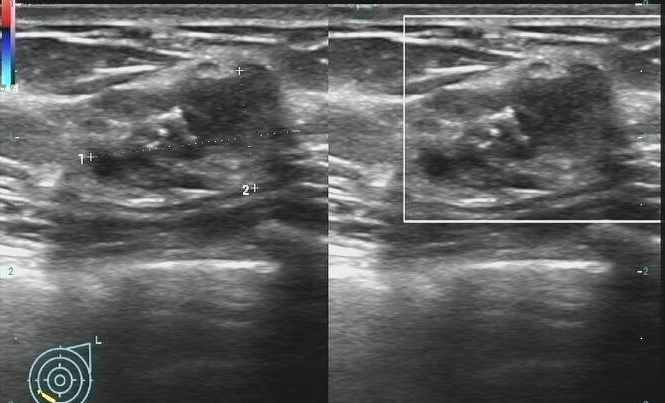

Supplement: S2 Data — Representative sonographic images of breast non-mass lesions. (ZIP) [file pone.0278299.s002.zip › Supplementary data 2/31/a.png]

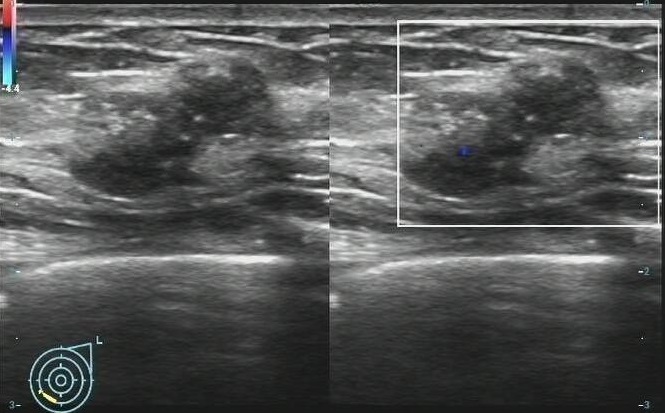

Supplement: S2 Data — Representative sonographic images of breast non-mass lesions. (ZIP) [file pone.0278299.s002.zip › Supplementary data 2/31/b.jpg]

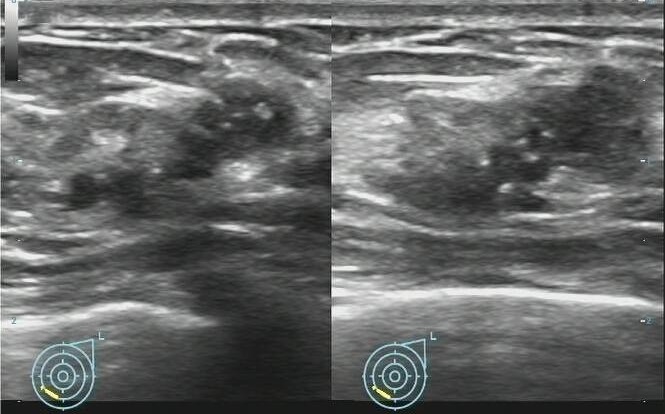

Supplement: S2 Data — Representative sonographic images of breast non-mass lesions. (ZIP) [file pone.0278299.s002.zip › Supplementary data 2/31/c.jpg]

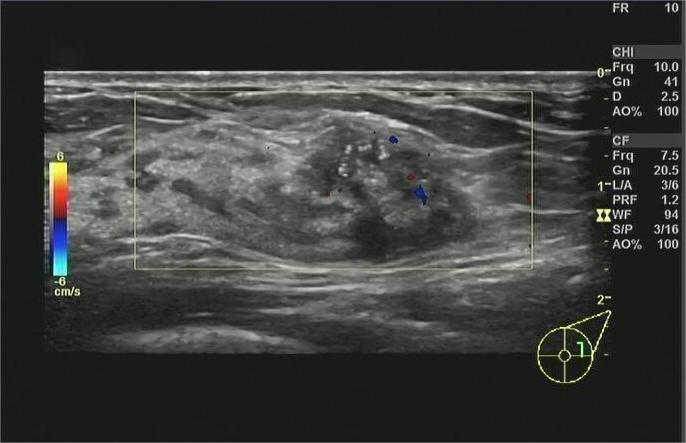

Supplement: S2 Data — Representative sonographic images of breast non-mass lesions. (ZIP) [file pone.0278299.s002.zip › Supplementary data 2/32/a.jpg]

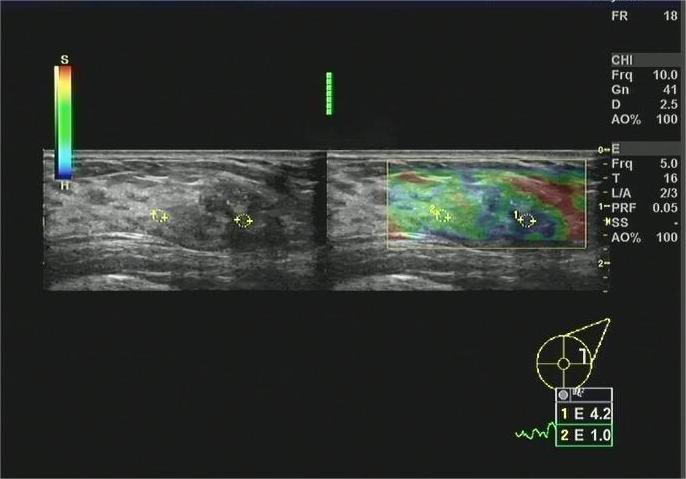

Supplement: S2 Data — Representative sonographic images of breast non-mass lesions. (ZIP) [file pone.0278299.s002.zip › Supplementary data 2/32/b.jpg]

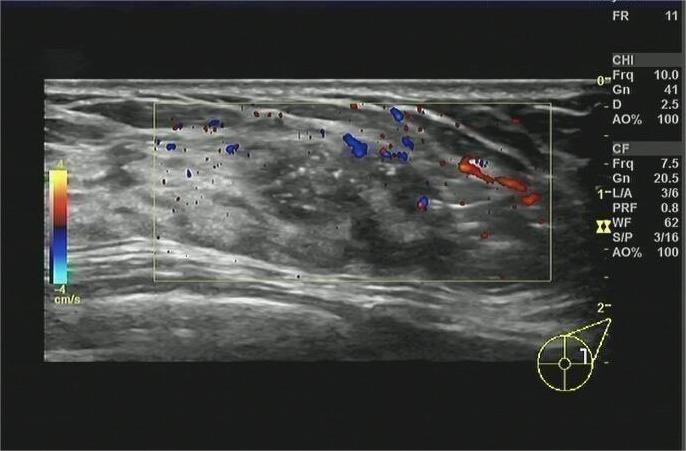

Supplement: S2 Data — Representative sonographic images of breast non-mass lesions. (ZIP) [file pone.0278299.s002.zip › Supplementary data 2/32/c.jpg]

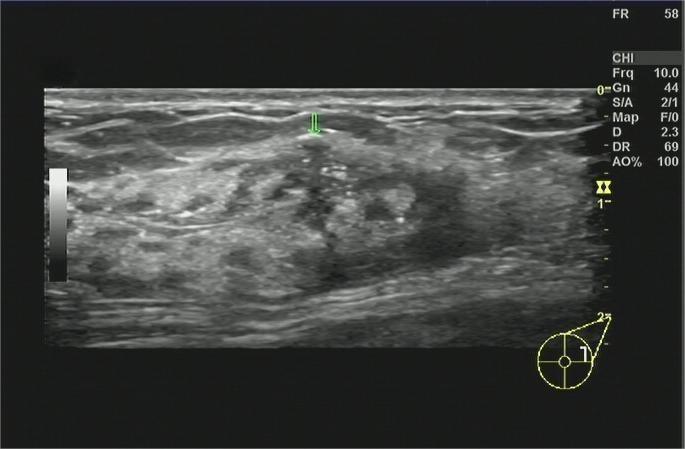

Supplement: S2 Data — Representative sonographic images of breast non-mass lesions. (ZIP) [file pone.0278299.s002.zip › Supplementary data 2/32/d.jpg]

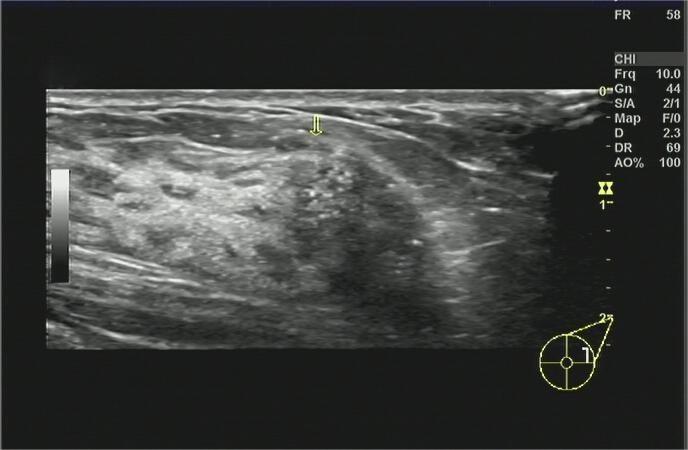

Supplement: S2 Data — Representative sonographic images of breast non-mass lesions. (ZIP) [file pone.0278299.s002.zip › Supplementary data 2/32/e.jpg]

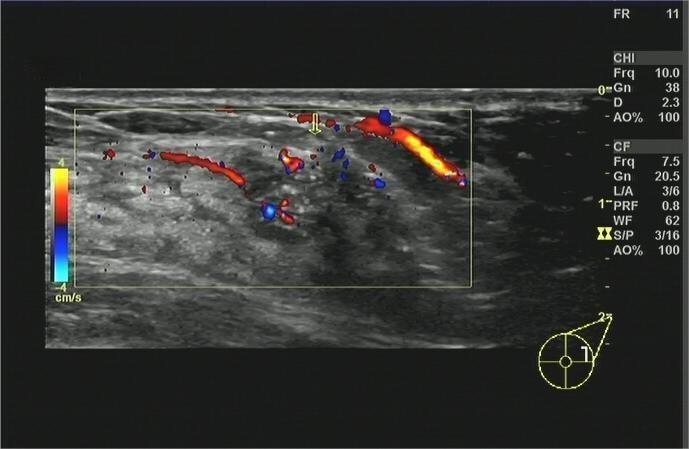

Supplement: S2 Data — Representative sonographic images of breast non-mass lesions. (ZIP) [file pone.0278299.s002.zip › Supplementary data 2/32/f.jpg]
